# Naamines and Naamidines as Novel Agents against a Plant Virus and Phytopathogenic Fungi

**DOI:** 10.3390/md16090311

**Published:** 2018-09-03

**Authors:** Pengbin Guo, Gang Li, Yuxiu Liu, Aidang Lu, Ziwen Wang, Qingmin Wang

**Affiliations:** 1 State Key Laboratory of Elemento-Organic Chemistry, Research Institute of Elemento-Organic Chemistry, College of Chemistry, Nankai University, Tianjin 300071, China; 13212007181@163.com (P.G.); 2120150654@mail.nankai.edu.cn (G.L.); liuyuxiu@nankai.edu.cn (Y.L.); aidang_lu@163.com (A.L.); 2 Tianjin Key Laboratory of Structure and Performance for Functional Molecules, College of Chemistry, Tianjin Normal University, Tianjin 300387, China; 3 Collaborative Innovation Center of Chemical Science and Engineering (Tianjin), Tianjin 300071, China; 4 Key Laboratory of Inorganic-Organic Hybrid Functional Materials Chemistry (Tianjin Normal University), Ministry of Education, Tianjin 300387, China

**Keywords:** marine natural products, naamines, naamidines, anti-tobacco mosaic virus (TMV) activity, fungicidal activity

## Abstract

Naamines, naamidines and various derivatives of these marine natural products were synthesized and characterized by means of nuclear magnetic resonance (NMR) spectroscopy and mass spectrometry. The activities of these alkaloids against a plant virus and phytopathogenic fungi were evaluated for the first time. A benzyloxy naamine derivative **15d** displayed excellent in vivo activity against tobacco mosaic virus at 500 μg/mL (inactivation activity, 46%; curative activity, 49%; and protective activity, 41%); its activities were higher than the corresponding activities of the commercial plant virucide ribavirin (32%, 35%, and 34%, respectively), making it a promising new lead compound for antiviral research. In vitro assays revealed that the test compounds exhibited very good antifungal activity against 14 kinds of phytopathogenic fungi. Again, the benzyloxy naamine derivative **15d** exhibited broad-spectrum fungicidal activity, emerging as a new lead compound for fungicidal research. Additional in vivo assays indicated that many of the compounds displayed inhibitory effects >30%.

## 1. Introduction

Although the population of the world has more than doubled since the 1960s, and global agricultural production has risen to a similar extent, productive arable acreage has increased by only 10%, and this differential has contributed to the demand for increases in food production [1,2]. Plant diseases, including diseases caused by phytopathogenic fungi and viruses, can lead to severe yield losses in agricultural and horticultural crops. Tobacco mosaic virus (TMV), noted for the first time on tobacco, was the earliest plant virus to be discovered and is the most well studied. TMV can infect more than 400 plant species belonging to 36 families [3]. Ribavirin is a widely used antiviral agent against TMV, but its antiviral effect is less than 50% at 500 μg/mL. In fact, there are no agents that can completely inhibit TMV once it has infected the plants. Therefore, the development of more active antiviral agents is urgently needed [4].

**Figure 1:**
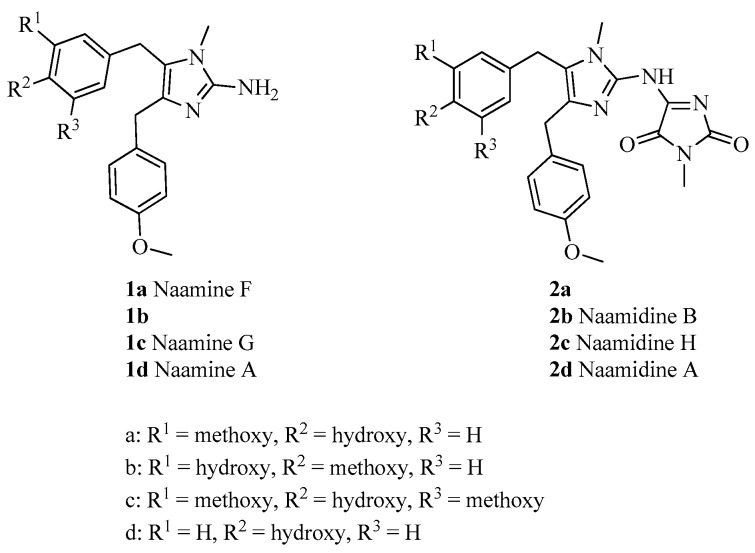
Structures of naamines **1a**–**d** and naamidines **2a**–**d**.

A number of marine natural products have been developed as pesticides and pharmaceuticals. For example, nereistoxin was the first marine natural product to find commercial use as a pesticide [5,6], the marine-derived compound ziconotide has been approved in the United States for the treatment of pain, and trabectedin is a marine-derived anticancer drug approved for use in the European Union [7]. With the development of improved separation methods, the number of bioactive natural products isolated from marine organisms has been increasing steadily. For example, work on the sponge *Leucetta chagosensis* has led to the isolation of interesting 2-amino imidazole alkaloids such as naamines (Figure 1) [8,9,10], isonaamines [8,9], naamidines (Figure 1) [9,11,12], and isonaamidines [13], all of which have a central imidazole ring. These alkaloids exhibit interesting biological activities, including antimicrobial activity [8,9,10], nitric oxide synthase inhibition activity [12], and cytotoxicity [10]. Naamidine A exhibits antitumor activity derived from its ability to regulate the kinases extracellular regulated protein kinase 1 (ERK1) and ERK2, a pathway that is not targeted by any current anticancer drugs [14]. Because these marine alkaloids have been isolated in only small quantities, their activity has not been extensively researched, and there have been no reports on the use of these alkaloids to prevent plant diseases.

In work aimed at developing novel inhibitors of plant viruses from natural products, various natural products with novel structures, such as phenanthroindolizidines [15], harmine [16], topsentins [17], and matrine [18], have been found to have good antiviral activity. As a continuing work to find novel inhibitors of plant viruses from natural products, we synthesized various naamines, naamidines and derivatives, and systematically investigated their antiviral and antifungal activities.

## 2. Results and Discussion

### 2.1. Chemistry

Naamine A and naamidine A were first synthesized in 2000 [19], and naamines B [20], C and E–G [21] and naamidines G and H [22,23] were subsequently prepared by means of sequential metallization of imidazole or by alkyne amination. However, the reported methods are unsuitable for the preparation of analogues for studying structure-activity relationships (SARs) because they are low yielding, involve harsh reaction conditions, or require structurally complex starting materials. Therefore, new routes for the synthesis of naamines and naamidines are needed.

**Figure 2:**
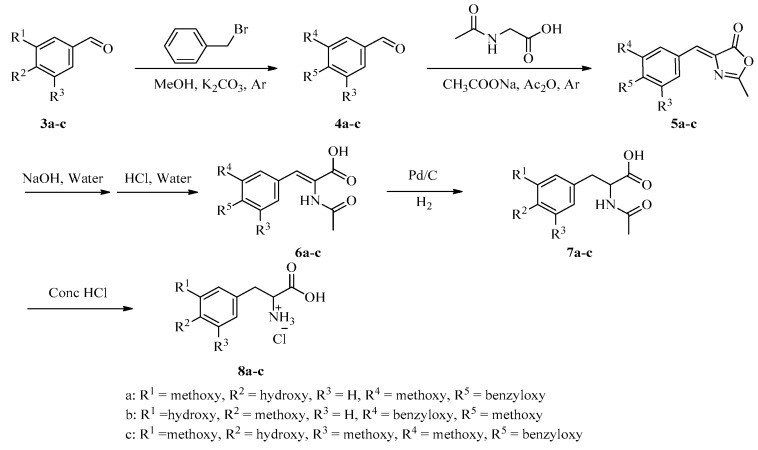
Synthesis of amino acids **8a**–**c**.

**Figure 3:**
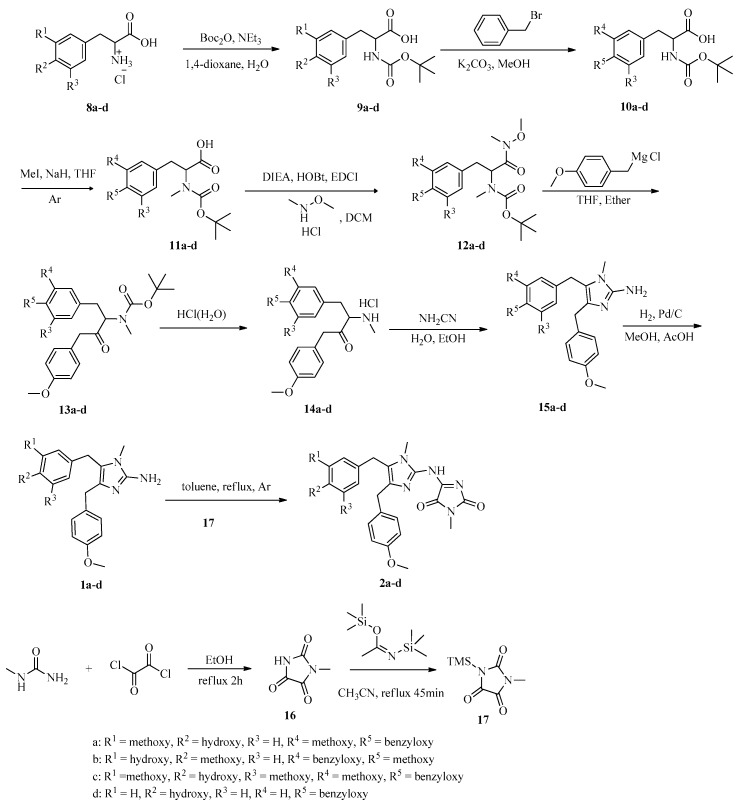
Synthesis of naamines **1a**–**d** and naamidines **2a**–**d**.

Herein, we describe the preparation of various analogues via the route shown in Figure 2 and Figure 3. First, substituted benzaldehydes **3a**–**c** were first prepared. Then, the phenol moiety of **3** was protected with a benzyl group, and condensation reactions of the protected compounds with acetoacetic acid gave oxazoles **5**, which were hydrolyzed with aqueous NaOH and acidified with dilute aqueous HCl to give acids **6**. Hydrogenation over Pd/C reduced the double bond and removed the benzyl protecting group to give phenylalanine **8a**–**c** after treatment with concentrated HCl. Naamines **1a**–**d** and naamidines **2a**–**d** were then prepared from **8a**–**d** by means of a pathway involving cyanamide cyclization as the key step (Figure 3). Boc protection of the amino group and benzyl protection of the phenol group of **8** gave acids **10**, which were methylated with iodomethane to obtain **11**. Condensation of **11** with *N*,*O*-dimethylhydroxylamine hydrochloride gave Weinreb amides **12**. Subsequent Grignard reaction and removal of the Boc protecting group gave aminoketones **14**, which were cyclized with cyanamide to give key intermediates **15**. Hydrogenation of **15** to remove the benzyl protecting group gave naamines **1**. Condensation of naamines **1** with **17**, which was prepared by the procedure depicted in Figure 3 afforded naamidines **2**.

**Figure 4:**
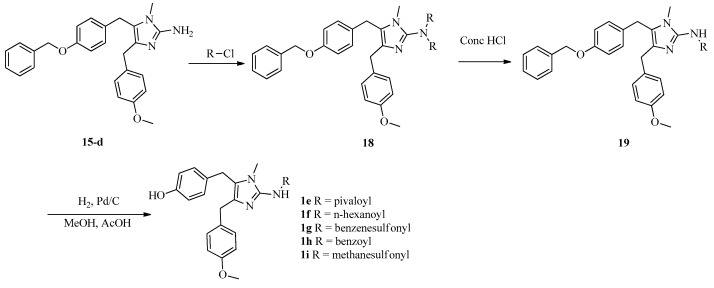
Synthesis of naamines **1e**–**i**.

**Figure 5:**
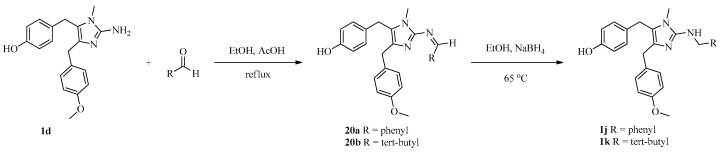
Synthesis of naamines **1j** and **1k**.

**Figure 6:**
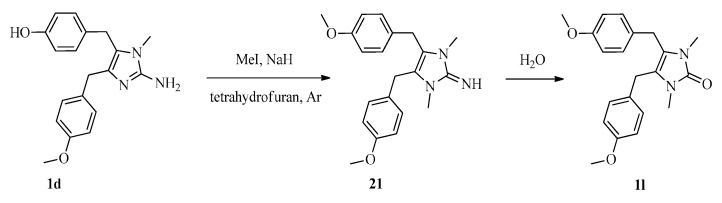
Synthesis of naamine **1l**.

**Figure 7:**
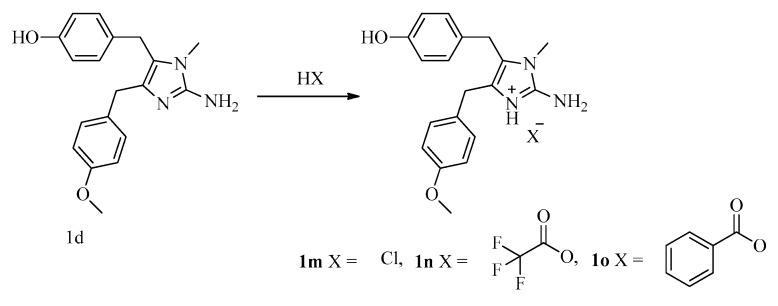
Synthesis of naamines **1m**–**o**.

**Figure 8:**
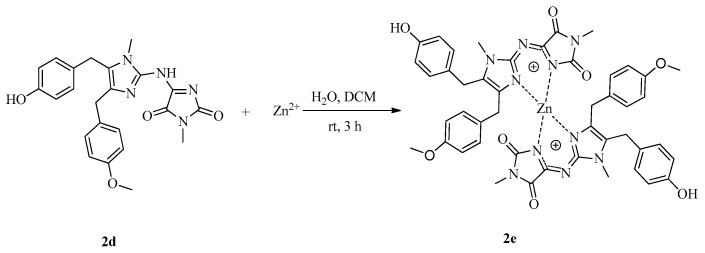
Synthesis of naamidine-metal complex **2e**.

To investigate SARs, we also designed and synthesized derivatives **1e**–**o** and **2e**. As depicted in Figure 4, acetylation of 2-aminoimidazole **15d** with various acyl chlorides gave a mixture of **18** and **19**, regardless of the temperature or the amount of acyl chloride. Fortunately, amides **18** could be converted to **19** by treatment with concentrated HCl. Deprotection of **19** afforded **1e**–**i**. Reductive amination of naamine **1d** gave naamines **1j** and **1k** (Figure 5). As shown in Figure 6, we attempted to prepare dimethyl imidazole amine **2l** by methylation of naamine **1d**, but we obtained only dimethyl ketone **1l** because **2l** was too sensitive to H_2_O. To investigate the impact of salification and metal complexation, we also synthesized naamines **1m**–**o** (Figure 7) and naamidine-metal complex **2e** (Figure 8).

### 2.2. Phytotoxic Activity

Compounds **1**, **2** and **15** were found to show no phytotoxic activity at 500 µg/mL.

### 2.3. Antiviral Activity

The anti-tobacco mosaic virus (TMV) activities of **1**, **2** and **15** were compared with those of the commercial plant virucide ribavirin (positive control) and 0.1% Tween-80 solution (negative control) (Table 1).

#### 2.3.1. In Vitro Anti-tobacco Mosaic Virus (TMV) Activity

Most of the synthesized compounds exhibited moderate antiviral activity in vitro, and **15d**, which showed higher inhibitory effect than ribavirin, emerged as a new lead compound for antiviral research. Among the naamine alkaloids **1a**–**d**, compounds **1a** and **1b** showed good activity, whereas **1c** and **1d** displayed no inhibitory effect, these results indicated that the positions and numbers of methoxy and hydroxyl groups on aromatic ring are critical to maintaining biological activity and this region is very sensitive to electronegativity. Naamine derivatives **1e**–**o** displayed relatively lower antiviral activity, which shows that derivatization of the amino group and salification decreased active. Naamidine alkaloids **2a** and **2c** showed no activity, whereas **2b** and **2d** were moderately active. The main difference between **1a**–**d** and **2a**–**d** lies in the introduction of the imidazolone ring, which leads to obvious changes of activity (inhibitory effect: **2a** < **1a**, **2b** < **1b**). Zinc complex **2e** displayed moderate activity. Interestingly, the introduction of a benzyloxy group on the aromatic ring was favorable for activity (inhibitory effect: **1a** ≈ **15a**, **1b** < **15b**, **1c** < **15c**, **1d** < **15d**). However, further introduction of a methoxy group decreased activity (inhibitory effect: **15c** < **15a** < **15d**). The main difference between **15a** and **15b** lies in the changes in the position of benzoxy group and methoxyl, which leads to obvious changes in activity (inhibitory effect: **15a** < **15b**).

#### 2.3.2. In Vivo Anti-TMV Activity

As shown in Table 1, most of the compounds showed in vivo anti-TMV activity that was similar to or higher than that of ribavirin. As in the in vitro assay, compound **15d** showed the best activity in vivo at 500 μg/mL (inactivation activity: 46%; curative activity: 49%; and protection activity: 41%), which is significantly higher than that of ribavirin (32%, 35%, and 34%, respectively). The in vivo activities of compounds **1g**, **1m**, **2a**, **2e** and **15a** are significantly higher than those in vitro, which reveals that these compounds may display certain inducible activity.

Unlike in the in vitro assay, in the in vivo assay, **1g**, **2e** and **15b** displayed activities that were similar to that of ribavirin. The other SARs in vivo are similar to those of in vitro.

### 2.4. Fungicidal Activity

Compounds **1**, **2** and **15** were also evaluated for their in vitro and in vivo fungicidal activities, which were compared with those of the commercial fungicides chlorothalonil, carbendazim and azoxystrobin (positive controls) and sterile water (negative control).

#### 2.4.1. In Vitro Fungicidal Activity

All the tested compounds displayed very good in vitro activity against 14 kinds of plant fungi at 50 μg/mL (Table 2), and most of the compounds displayed high bioselectivity. The fungicidal activity of naamidine H (**2c**) against *Cercospora arachidicola* Hori was higher than that of carbendazim. Compounds **1b**, **1k**, **15a**, **15c** and **15d** exhibited higher fungicidal activity against *Physalospora piricola* than did carbendazim. The fungicidal activities of naamine A (**1d**), derivatives **1j**, **1m** and naamidine H (**2c**) against *Rhizoctonia cerealis* were higher than that of carbendazim and similar to that of chlorothalonil. Against *Phytophthora capsici*, compounds **15a**−**d** were more active than naamines **1** and naamidines **2**, which indicated that the hydroxyl on the aromatic ring is bad for antifungal activity. Derivative **15d**, with its broad-spectrum fungicidal activity, emerged as a new lead compound for fungicidal research. Among the naamine alkaloids **1a**−**d**, **1b** showed good activity against *Physalospora piricola*, **1c** showed good activity against *Alternaria solani*, **1d** showed good activity against *Rhizoctonia cerealis* and *Fusarium graminearum*, which indicated that different fungus displays different selectivity for compounds. Similar activity rules also can be found from **2a**−**d**. The replacement of the amino group at 2-position of imidazole ring with oxygen and salification with trifluoroacetic acid are favorable for antifungal activity against *Sclerotinia sclerotiorum* and *Botrytis cinerea*. 

#### 2.4.2. In Vivo Fungicidal Activity

The activities of compounds **1**, **2** and **15** were also evaluated in vivo against *Sclerotinia sclerotiorum* on rape, *Rhizoctonia cerealis* on cerealis, *Botrytis cinerea* on cucumber, *Phytophthora capsici* on capsici, *Corynespora cassiicola* on cucumber, and *Blum eria graminis f.* sp. *tritici* on wheat (Table 3). The assays revealed that many of the compounds had an inhibitory effect of >30%. Compounds **15b**−**d** exhibited greater inhibitory effects against *Phytophthora capsici* on capsici than did naamines **1** and naamidines **2**. However, none of the compounds showed an inhibitory effect against *Blumeria graminis f.* sp. *tritici* on wheat.

## 3. Experimental Section

### 3.1. General Experimental Procedures

The melting points were determined on an X-4 binocular microscope (Beijing Tech Instruments Co., Beijing, China). NMR spectra were obtained by using Bruker AV 400 spectrometer (Bruker Co., Fallanden, Switzerland). Chemical shifts (*δ*) were given in parts per million (ppm) and measured downfield from internal tetramethylsilane. High-resolution mass spectra were obtained with an FT-ICR MS spectrometer (Ionspec, 7.0 T, Kuala Lumpur, Malaysia). All reagents were of analytical reagent grade or chemically pure and purified prior to use when necessary.

See Supplementary Materials for all NMR spectra.

General procedure for the preparation of benzaldehydes **4**. The mixture of benzaldehydes **3** (0.06 mol), K_2_CO_3_ (11.0 g, 0.07 mol), benzyl bromide (9.4 g, 0.07 mol) and methanol 250 mL was stirred and refluxed for 4 h under argon. Then the reaction mixture was filtered and evaporated under vacuum. The residue was dissolved in CH_2_Cl_2_ (200 mL) and washed with H_2_O (100 mL), brine (100 mL), and dried with MgSO_4_ anhydrous. The solution was filtered, evaporated under vacuum. Then acetone (10 mL) was added. The mixture was kept at 0 °C for 4 h, filtered to give benzaldehydes **4** as a white powder.

For 4-(benzyloxy)-3-methoxybenzaldehyde (**4a**): Yield 85%; m.p.: 67–68 °C; ^1^H NMR (400 MHz, CDCl_3_) *δ* 9.83 (s, 1H, COH), 7.45–7.31 (m, 7H, Ar-H), 6.99 (d, *J* = 8.2 Hz, 1H, Ar-H), 5.25 (s, 2H, O-CH_2_), 3.95 (s, 3H, O-CH_3_).

For 3-(benzyloxy)-4-methoxybenzaldehyde (**4b**): Yield 95%; m.p.: 63–65 °C; ^1^H NMR (400 MHz, CDCl_3_) *δ* 9.82 (s, 1H, COH), 7.47–7.30 (m, 7H, Ar-H), 6.99 (d, *J* = 8.1 Hz, 1H, Ar-H), 5.19 (s, 2H, O-CH_2_), 3.96 (s, 3H, O-CH_3_).

For 4-(benzyloxy)-3,5-dimethoxybenzaldehyde (**4c**): Yield 88%; m.p.: 63–64 °C; ^1^H NMR (400 MHz, CDCl_3_) *δ* 9.86 (s, 1H, COH), 7.47 (d, *J* = 7.1 Hz, 2H, Ar-H), 7.32 (dd, *J* = 11.2, 7.0 Hz, 3H, Ar-H), 7.11 (s, 2H, Ar-H), 5.13 (s, 2H, O-CH_2_), 3.90 (s, 6H, O-CH_3_).

General procedure for the preparation of **5**. The mixture of benzaldehydes **4** (0.02 mol), acetic anhydride (10 mL), *N*-acetyl-glycine (0.02 mol) and sodium acetate (0.02 mol) was stirred at 115 °C for 3.5 h under argon. Then, the reaction mixture was cooled to room temperature, and ethanol (100 mL) was added. After ultrasonic oscillation, the mixture was filtered to give **5** as a yellow powder.

For (*Z*)-4-(4-(benzyloxy)-3-methoxybenzylidene)-2-methyloxazol-5(4*H*)-one (**5a**): Yield 44%; m.p.: 157–159 °C; ^1^H NMR (400 MHz, CDCl_3_) *δ* 7.92 (d, *J* = 1.8 Hz, 1H, Ar-H), 7.48–7.29 (m, 6H, Ar-H), 7.07 (s, 1H, CH), 6.91 (d, *J* = 8.4 Hz, 1H, Ar-H), 5.23 (s, 2H, O-CH_2_), 3.96 (s, 3H, O-CH_3_), 2.39 (s, 3H, C-CH_3_).

For (*Z*)-4-(3-(benzyloxy)-4-methoxybenzylidene)-2-methyloxazol-5(*4H*)-one (**5b**): Yield 28%; m.p.: 108–110 °C; ^1^H NMR (400 MHz, CDCl_3_) *δ* 7.99 (s, 1H, CH), 7.52–7.28 (m, 6H, Ar-H), 7.03 (s, 1H, Ar-H), 6.92 (d, *J* = 8.4 Hz, 1H, Ar-H), 5.22 (s, 2H, O-CH_2_), 3.94 (s, 3H, O-CH_3_), 2.39 (s, 3H, C-CH_3_); ^13^C NMR (100 MHz, CDCl_3_) *δ* 168.1, 164.8, 152.6, 148.2, 136.7, 131.6, 130.5, 128.6, 128.0, 127.6, 126.3, 116.4, 111.3, 71.0, 56.0, 15.6; HRMS (ESI) calcd. for C_19_H_18_NO_4_^+^ [M + H]^+^ 324.1230, found 324.1232.

For (*Z*)-4-(4-(benzyloxy)-3,5-dimethoxybenzylidene)-2-methyloxazol-5(*4H*)-one (**5c**): Yield 52%; m.p.: 102–104 °C; ^1^H NMR (400 MHz, CDCl_3_) *δ* 7.48 (d, *J* = 7.5 Hz, 2H, Ar-H), 7.41–7.28 (m, 5H, Ar-H), 7.04 (s, 1H, CH), 5.10 (s, 2H, O-CH_2_), 3.88 (s, 6H, O-CH_3_), 2.40 (s, 3H, C-CH_3_); ^13^C NMR (100 MHz, CDCl_3_) *δ* 167.9, 165.6, 153.6, 139.9, 137.4, 131.6, 131.5, 128.7, 128.5, 128.2, 128.0, 109.6, 75.1, 56.2, 15.8; HRMS (ESI) calcd. for C_20_H_20_NO_5_^+^ [M + H]^+^ 354.1336, found 354.1338.

General procedure for the preparation of acids **6**. The mixture of **5** (13.10 mmol), NaOH (1.6 g, 39.20 mmol) and H_2_O (100 mL) was stirred and refluxed for 1 h. Then, the reaction mixture was cooled to room temperature and acidified to pH 5–6 with dilute hydrochloric acid. The mixture was filtered to give acids **6** as a white powder.

For (*Z*)-2-acetamido-3-(4-(benzyloxy)-3-methoxyphenyl)acrylic acid (**6a**): Yield 93%; m.p.: 200–202 °C; ^1^H NMR (400 MHz, DMSO-*d*_6_) *δ* 12.52 (s, 1H, COOH), 9.41 (s, 1H, NH), 7.45–7.35 (m, 5H, Ar-H), 7.33 (s, 1H, Ar-H), 7.21 (s, 1H, CH), 7.18 (d, *J* = 8.4 Hz, 1H, Ar-H), 7.07 (d, *J* = 8.4 Hz, 1H, Ar-H), 5.13 (s, 2H, O-CH_2_), 3.77 (s, 3H, O-CH_3_), 1.99 (s, 3H, C-CH_3_).

For (*Z*)-2-acetamido-3-(3-(benzyloxy)-4-methoxyphenyl)acrylic acid (**6b**): Yield 61%; m.p.: 209–211 °C; ^1^H NMR (400 MHz, DMSO) *δ* 12.53 (s, 1H, COOH), 9.46 (s, 1H, NH), 7.55–7.30 (m, 6H, Ar-H), 7.24 (d, *J* = 8.2 Hz, 1H, Ar-H), 7.19 (s, 1H, CH), 7.02 (d, *J* = 8.2 Hz, 1H, Ar-H), 5.10 (s, 2H, O-CH_2_), 3.80 (s, 3H, O-CH_3_), 1.97 (s, 3H, CO-CH_3_).

For (*Z*)-2-acetamido-3-(4-(benzyloxy)-3,5-dimethoxyphenyl)acrylic acid (**6c**): Yield 53%; m.p.: 155–157 °C; ^1^H NMR (400 MHz, CD_3_OD) *δ* 7.46 (s, 1H, CH), 7.44 (d, *J* = 6.7 Hz, 2H, Ar-H), 7.36–7.24 (m, 3H, Ar-H), 6.93 (s, 2H, Ar-H), 4.99 (s, 2H, O-CH_2_), 3.82 (s, 6H, O-CH_3_), 2.11 (s, 3H, CO-CH_3_); ^13^C NMR (100 MHz, CD_3_OD) *δ* 173.2, 168.3, 154.7, 139.2, 139.0, 136.0, 130.7, 129.6, 129.2, 129.1, 126.2, 108.5, 76.1, 56.6, 22.6; HRMS (ESI) calcd. for C_20_H_22_NO_6_^+^ [M + H]^+^ 372.1442, found 372.1444.

General procedure for the preparation of acids **7**. The mixture of acids **6** (3.28 mmol) and Pd/C (10 wt%) (0.15 g) in ethanol (100 mL) was bubbled with hydrogen and stirred at room temperature for 12 h. Then, the mixture was filtered and concentrated to give acids **7** as a slight yellow powder.

For 2-acetamido-3-(4-(benzyloxy)-3-methoxyphenyl)propanoic acid (**7a**): Yield 98%; m.p.: 73–75 °C; ^1^H NMR (400 MHz, CD_3_OD) *δ* 6.79 (d, *J* = 1.8 Hz, 1H, Ar-H), 6.70 (d, *J* = 8.0 Hz, 1H, Ar-H), 6.64 (dd, *J* = 8.0, 1.8 Hz, 1H, Ar-H), 4.61 (dd, *J* = 8.9, 5.1 Hz, 1H, CH), 3.82 (s, 3H, O-CH_3_), 3.10 (dd, *J* = 14.0, 5.1 Hz, 1H, CH_2_), 2.85 (dd, *J* = 14.0, 8.9 Hz, 1H, CH_2_), 1.91 (s, 3H, C-CH_3_).

For 2-acetamido-3-(3-(benzyloxy)-4-methoxyphenyl)propanoic acid (**7b**): Yield 95%; m.p.: 147–150 °C; ^1^H NMR (400 MHz, CD_3_OD) *δ* 6.82 (d, *J* = 8.0 Hz, 1H, Ar-H), 6.69 (s, 1H, Ar-H), 6.65 (d, *J* = 8.0 Hz, 1H, Ar-H), 4.57 (dd, *J* = 8.7, 5.0 Hz, 1H, CH), 3.81 (s, 3H, O-CH_3_), 3.06 (dd, *J* = 13.9, 5.0 Hz, 1H, CH_2_), 2.82 (dd, *J* = 13.9, 8.7 Hz, 1H, CH_2_), 1.91 (s, 3H, CO-CH_3_); ^13^C NMR (100 MHz, CD_3_OD) *δ* 174.9, 173.2, 148.0, 147.4, 131.2, 121.5, 117.2, 112.7, 56.4, 55.4, 37.8, 22.4; HRMS (ESI) calcd. for C_12_H_16_NO_5_^+^ [M + H]^+^ 254.1023, found 254.1019.

For 2-acetamido-3-(4-(benzyloxy)-3,5-dimethoxyphenyl)propanoic acid (**7c**): Yield 96%; m.p.: 148–151 °C; ^1^H NMR (400 MHz, CD_3_OD) *δ* 6.54 (s, 2H, Ar-H), 4.68 (dd, *J* = 8.8, 5.0 Hz, 1H, CH), 3.86 (s, 6H, O-CH_3_), 3.16 (dd, *J* = 13.9, 5.0 Hz, 1H, CH_2_), 2.90 (dd, *J* = 13.9, 8.8 Hz, 1H, CH_2_), 1.97 (s, 3H, CO-CH_3_); ^13^C NMR (100 MHz, CD_3_OD) *δ* 175.0, 173.2, 149.2, 135.4, 129.0, 107.4, 56.8, 55.4, 38.6, 22.4; HRMS (ESI) calcd. for C_13_H_18_NO_6_^+^ [M + H]^+^ 284.1129, found 284.1133.

General procedure for the preparation of amino acids **8**. The mixture of acids **7** (19.78 mmol) in 4 N HCl solution (500 mL) was refluxed for 24 h and concentrated. Then, methanol (50 mL) was added. The mixture was kept at 0 °C for 4 h, filtered to give amino acids **8**, which were used directly for the next step.

General procedure for the preparation of amino acids **9**. The mixture of acids **8** (4.03 mmol), (Boc)_2_O (4.44 mmol), Et_3_N (12.1 mmol), H_2_O (20 mL) and 1,4-dioxane (20 mL) was stirred at room temperature for 18 h. Then, the mixture was concentrated and dissolved in H_2_O (100 mL), acidified to pH 5–6 with dilute hydrochloric acid, and extracted with ethyl acetate (100 mL × 3). The combined organic layer was washed with brine (100 mL), dried with MgSO_4_ anhydrous. The solution was filtered, evaporated under vacuum. Methanol (10 mL) was added. The mixture was kept at 0 °C for 4 h, filtered to give amino acids **9**, which were used directly for the next step.

General procedure for the preparation of Boc amino acids **10**. The mixture of amino acids **9** (1.89 mmol), K_2_CO_3_ (4.55 mmol), benzyl bromide (2.27 mmol) and methanol (50 mL) was refluxed for 4 h. Then, the mixture was concentrated and dissolved in H_2_O (50 mL), acidified to pH 5–6 with dilute hydrochloric acid, and extracted with ethyl acetate (50 mL × 3). The combined organic layer was washed with brine (100 mL), dried with MgSO_4_ anhydrous. The solution was filtered, evaporated under vacuum to give Boc amino acids **10**.

For 3-(4-(benzyloxy)-3-methoxyphenyl)-2-((tert-butoxycarbonyl)amino)propanoic acid (**10a**): Slight yellow powder; Yield for three steps 68%; m.p.: 128–130 °C; ^1^H NMR (400 MHz, CDCl_3_, exists as a complex mixture of two rotamers at room temperature) *δ* 7.51–7.27 (m, 5H, Ar-H), 6.81 (d, *J* = 8.1 Hz, 1H, Ar-H), 6.72 (s, 1H, Ar-H), 6.65 (d, *J* = 7.8 Hz, 1H, Ar-H), 6.02 and 4.93 (d, *J* = 7.3 Hz, 1H, NH), 5.12 (s, 2H, O-CH_2_), 4.55 and 4.34 (s, 1H, CH), 3.85 (s, 3H, O-CH_3_), 3.13–2.85 (m, 2H, CH_2_), 1.42 and 1.32 (two s, 9H, C-CH_3_).

For 3-(3-(benzyloxy)-4-methoxyphenyl)-2-((tert-butoxycarbonyl)amino)propanoic acid (**10b**): White powder; Yield for three steps 53%; m.p.: 143–145 °C; ^1^H NMR (400 MHz, CDCl_3_, exists as a complex mixture of two rotamers at room temperature) *δ* 7.46–7.28 (m, 5H, Ar-H), 6.81 (d, *J* = 8.6 Hz, 1H, Ar-H), 6.75–6.68 (m, 2H, Ar-H), 5.84 and 4.89 (two d, 1H, NH), 5.11 (s, 2H, O-CH_2_), 4.59–4.46 and 4.35–4.25 (two m, 1H, CHCH_2_), 3.85 (s, 3H, O-CH_3_), 3.12–2.76 (m, 2H, CHCH_2_), 1.42 and 1.35 (two s, 9H, C-CH_3_).

For 3-(4-(benzyloxy)-3,5-dimethoxyphenyl)-2-((tert-butoxycarbonyl)amino)propanoic acid (**10c**): Brown powder; Yield for three steps 23%; m.p.: 123–125 °C; ^1^H NMR (400 MHz, CDCl_3_, exists as a complex mixture of two rotamers at room temperature) *δ* 7.47 (d, *J* = 7.1 Hz, 2H, Ar-H), 7.36–7.27 (m, 3H, Ar-H), 6.38 (s, 2H, Ar-H), 4.98 (s, 2H, O-CH_2_), 4.94 (d, *J* = 6.9 Hz, 1H, NH), 4.59 (s, 1H, CH), 3.79 (s, 6H, O-CH_3_), 3.18–3.08 (m, 1H, CH_2_), 3.05–2.96 (m, 1H, CH_2_), 1.43 and 1.35 (two s, 9H, C-CH_3_).

For 3-(4-(benzyloxy)phenyl)-2-((tert-butoxycarbonyl)amino)propanoic acid (**10d**): White powder; Yield for three steps 93%; m.p.: 110–111 °C; ^1^H NMR (400 MHz, CDCl_3_) *δ* 7.45–7.28 (m, 5H, Ar-H), 7.10 (d, *J* = 8.3 Hz, 2H, Ar-H), 6.91 (d, *J* = 8.4 Hz, 2H, Ar-H), 6.11 and 4.92 (two d, *J* = 6.6 Hz, 1H, NH), 5.03 (s, 2H, O-CH_2_), 4.57 and 4.36 (two d, *J* = 5.4 Hz, 1H, CHCH_2_), 3.06 (m, 2H, CHCH_2_), 1.37 (d, *J* = 38.4 Hz, 9H, CCH_3_).

General procedure for the preparation of acids **11**. To the solution of acids **10** (10.02 mmol) in THF (30 mL) was added 70% NaH (30.06 mmol) and stirred for 30 min at 0 °C. Then CH_3_I (20.04 mmol) was added. The reaction mixture was stirred for 24 h at room temperature, and quenched with H_2_O (20 mL), and extracted with ethyl acetate (100 mL × 3). The combined organic layer was washed with Na_2_S_2_O_3_ solution (100 mL), NaHCO_3_ solution (100 mL), brine (100 mL) and dried with MgSO_4_ anhydrous. The solution was filtered, evaporated under vacuum to give acids **11**.

For 3-(4-(benzyloxy)-3-methoxyphenyl)-2-((tert-butoxycarbonyl)(methyl)amino)propanoic acid (**11a**): Yellow oil; Yield 88%; ^1^H NMR (400 MHz, CDCl_3_, exists as a complex mixture of two rotamers at room temperature) *δ* 7.47–7.27 (m, 5H, Ar-H), 6.83–6.62 (m, 3H, Ar-H), 5.12 (s, 2H, O-CH_2_), 4.73 and 4.52 (two d, *J* = 5.9 Hz, 1H, CH), 3.87 (s, 3H, O-CH_3_), 3.26–3.19 (m, 1H, CH_2_), 3.12–2.92 (m, 1H, CH_2_), 2.74 and 2.37 (two s, 3H, N-CH_3_), 1.41 and 1.33 (two s, 9H, C-CH_3_); ^13^C NMR (100 MHz, CDCl_3_) *δ* 176.3, 156.3, 155.1, 149.6, 146.9, 137.2, 130.6, 130.3, 128.5, 127.8, 127.3, 121.1, 121.0, 114.2, 112.7, 112.5, 80.7, 80.6, 71.1, 61.8, 60.3, 56.0, 34.8, 34.3, 32.8, 28.3, 28.2; HRMS (ESI) calcd. for C_23_H_29_NNaO_6_^+^ [M + Na]^+^ 438.1887, found 438.1880.

For 3-(3-(benzyloxy)-4-methoxyphenyl)-2-((tert-butoxycarbonyl)(methyl)amino)propanoic acid (**11b**): Brown oil; Yield 76%; ^1^H NMR (400 MHz, CDCl_3_, exists as a complex mixture of two rotamers at room temperature) *δ* 7.44–7.27 (m, 5H, Ar-H), 6.83–6.67 (m, 3H, Ar-H), 5.11 (s, 2H, O-CH_2_), 4.72–4.66 and 4.45–4.35 (two m, 1H, CHCH_2_), 3.84 (s, 3H, O-CH_3_), 3.25–3.10 (m, 1H, CHCH_2_), 3.04–2.84 (m, 1H, CHCH_2_), 2.64 and 2.57 (two s, 3H, N-CH_3_), 1.39 and 1.33 (two s, 9H, C-CH_3_); ^13^C NMR (100 MHz, CDCl_3_) *δ* 176.4, 156.4, 155.0, 148.6, 148.5, 148.0, 137.2, 137.1, 130.0, 129.6, 127.9, 127.4, 127.4, 121.8, 121.7, 115.1, 114.8, 112.1, 111.9, 80.7, 77.31, 71.2, 71.0, 61.8, 60.6, 60.5, 56.1, 34.8, 34.2, 33.0, 14.2; HRMS (ESI) calcd. for C_23_H_29_NNaO_6_^+^ [M + Na]^+^ 438.1887, found 438.1883.

For 3-(4-(benzyloxy)-3,5-dimethoxyphenyl)-2-((tert-butoxycarbonyl)(methyl)amino)propanoic acid (**11c**): Brown oil; Yield 79%; ^1^H NMR (400 MHz, CDCl_3_, exists as a complex mixture of two rotamers at room temperature) *δ* 7.47 (d, *J* = 7.0 Hz, 2H, Ar-H), 7.36–7.27 (m, 3H, Ar-H), 6.42 and 6.37 (two s, 2H, Ar-H), 4.98 (s, 2H, O-CH_2_), 4.80–4.70 and 4.55–4.45 (two m, 1H, CH), 3.80 (s, 6H, O-CH_3_), 3.33–3.17 (m, 1H, CH_2_), 3.15–2.95 (m, 1H, CH_2_), 2.73 and 2.67 (two s, 3H, N-CH_3_), 1.43 and 1.36 (two s, 9H, C-CH_3_); ^13^C NMR (100 MHz, CDCl_3_) *δ* 176.0, 156.2, 154.9, 153.5, 153.4, 137.8, 137.7, 135.7, 135.5, 133.3, 132.9, 130.2, 128.5, 128.1, 127.8, 106.0, 105.8, 80.7, 75.0, 61.8, 60.3, 56.1, 35.5, 34.9, 33.1, 33.0, 28.3, 28.2; HRMS (ESI) calcd. for C_24_H_31_NNaO_7_^+^ [M + Na]^+^ 468.1993, found 468.1989.

For 3-(4-(benzyloxy)phenyl)-2-((tert-butoxycarbonyl)(methyl)amino)propanoic acid (**11d**): White powder; m.p.: 126–127 °C Yield 79%; ^1^H NMR (400 MHz, CDCl_3_, exists as a 1:1 mixture of two rotamers) *δ* 7.47–7.29 (m, 5H, Ar-H), 7.14–7.08 (m, 2H, Ar-H), 6.91 (d, *J* = 8.0 Hz, 2H, Ar-H), 5.04 (s, 2H, O-CH_2_), 4.71–4.66 and 4.60–4.53 (two m, 1H, CHCH_2_), 3.33–3.17 (m, 1H, CHCH_2_), 3.16–2.92 (m, 1H, CHCH_2_), 2.75 and 2.67 (two s, 3H, N-CH_3_), 1.41 and 1.35 (two s, 9H, C-CH_3_).

General procedure for the preparation of carbamates **12**. The mixture of acids **11** (0.75 mol), *N*,*N*-diisopropylethylamine (232.87 mmol), 1-hydroxybenzotriazole (82.63 mmol), 1-ethyl-3-(3-dimethyllaminopropyl)carbodiimide hydrochloride (EDC·HCl) (82.63 mmol) and *N*,*O*-dimethylhydroxylamine hydrochloride (82.63 mmol) in dichloromethane (200 mL) was stirred at room temperature for 12 h. Then, the reaction mixture was acidified to pH 5–6 with dilute hydrochloric acid and filtered. The filtrate was extracted with dichloromethane (100 mL × 3). The combined organic layer was washed with brine (100 mL) and dried with MgSO_4_ anhydrous. The solution was filtered and evaporated under vacuum. The residue was purified by column chromatography on silica gel to give carbamates **12**.

For *tert*-butyl (3-(4-(benzyloxy)-3-methoxyphenyl)-1-(methoxy(methyl)amino)-1-oxopropan-2-yl)(methyl)carbamate (**12a**): Yellow oil; Yield 73%; ^1^H NMR (400 MHz, CDCl_3_, exists as a complex mixture of two rotamers at room temperature) *δ* 7.45–7.28 (m, 5H, Ar-H), 6.83–6.61 (m, 3H, Ar-H), 5.52 and 5.09 (two s, 1H, CH), 5.12 (s, 2H, O-CH_2_), 3.87 (s, 3H, O-CH_3_), 3.62 and 3.59 (two s, 3H, O-CH_3_), 3.18 and 3.15 (two s, 3H, N-CH_3_), 3.15–2.86 (m, 2H, CH_2_), 2.84 (s, 3H, N-CH_3_), 1.37 and 1.24 (two s, 9H, C-CH_3_); ^13^C NMR (100 MHz, CDCl_3_) *δ* 155.6, 154.9, 149.5, 149.4, 146.8, 146.7, 137.3, 131.2, 130.6, 128.5, 127.7, 127.2, 121.3, 114.2, 114.1, 113.1, 112.9, 79.7, 71.1, 61.3, 60.4, 57.3, 55.9, 54.4, 34.5, 32.3, 30.2, 29.9, 28.3, 28.1, 21.0, 14.2; HRMS (ESI) calcd. for C_25_H_35_N_2_O_6_^+^ [M + H]^+^ 459.2490, found 459.2498.

For *tert*-butyl (3-(3-(benzyloxy)-4-methoxyphenyl)-1-(methoxy(methyl)amino)-1-oxopropan-2-yl)(methyl)carbamate (**12b**): Yellow oil; Yield 80%; ^1^H NMR (400 MHz, CDCl_3_, exists as a complex mixture of two rotamers at room temperature) *δ* 7.50–7.28 (m, 5H, Ar-H), 6.87–6.70 (m, 3H, Ar-H), 5.49 and 5.06 (two s, 1H, CHCH_2_), 5.12 (s, 2H, O-CH_2_), 3.85 (s, 3H, O-CH_3_), 3.62 and 3.57 (two s, 3H, O-CH_3_), 3.17 and 3.14 (two s, 3H, N-CH_3_), 3.11–2.81 (m, 2H, CHCH_2_), 2.79 and 2.78 (two s, 3H, N-CH_3_), 1.38 and 1.28 (two s, 9H, C-CH_3_); ^13^C NMR (100 MHz, CDCl_3_) *δ* 155.7, 155.0, 148.5, 148.3, 148.2, 148.0, 137.3, 137.2, 130.6, 129.9, 128.6, 127.8, 127.4, 127.3, 122.1, 122.0, 115.4, 115.1, 112.1, 111.9, 79.8, 79.7, 77.3, 71.1, 71.0, 61.6, 61.3, 57.4, 56.2, 56.1, 54.5, 34.5, 32.4, 32.1, 30.2, 30.0, 28.3, 28.2; HRMS (ESI) calcd. for C_25_H_35_N_2_O_6_^+^ [M + H]^+^ 459.2490, found 459.2490.

For *tert*-butyl (3-(4-(benzyloxy)-3,5-dimethoxyphenyl)-1-(methoxy(methyl)amino)-1-oxopropan-2-yl)(methyl)carbamate (**12c**): Brown oil; Yield 67%; ^1^H NMR (400 MHz, CDCl_3_) *δ* 7.53–7.45 (m, 2H, Ar-H), 7.38–7.28 (m, 3H, Ar-H), 6.46 and 6.37 (two s, 2H, Ar-H), 5.57 and 5.12 (two s, 1H, CH), 4.96 (s, 2H, O-CH_2_), 3.80 (s, 6H, O-CH_3_), 3.63 and 3.60 (two s, 3H, O-CH_3_), 3.20 and 3.16 (two s, 3H, N-CH_3_), 3.14–3.07 (m, 1H, CH_2_), 2.96–2.88 (m, 1H, CH_2_), 2.85 and 2.84 (two s, 3H, N-CH_3_), 1.39 and 1.27 (two s, 9H, C-CH_3_); ^13^C NMR (100 MHz, CDCl_3_) *δ* 155.6, 154.9, 153.4, 153.2, 137.9, 137.8, 135.6, 135.4, 134.0, 133.1, 128.5, 128.1, 128.1, 127.8, 127.7, 106.3, 106.1, 79.8, 75.0, 75.0, 61.5, 61.3, 60.8, 60.4, 57.4, 56.1, 56.0, 53.9, 35.3, 32.3, 32.0, 30.2, 30.0, 28.3, 28.15, 21.0, 14.2; HRMS (ESI) calcd. for C_26_H_37_N_2_O_7_^+^ [M + H]^+^ 489.2595, found 489.2603.

For *tert*-butyl (3-(4-(benzyloxy)phenyl)-1-(methoxy(methyl)amino)-1-oxopropan-2-yl)(methyl)carbamate (**12d**): Yellow oil; Yield 85%; ^1^H NMR (400 MHz, CDCl_3_, exists as a complex mixture of two rotamers at room temperature) *δ* 7.43–7.30 (m, 5H, Ar-H), 7.16 and 7.08 (two d, *J* = 8.2 Hz, 2H, Ar-H), 6.93–6.85 (m, 2H, Ar-H), 5.50 and 5.12 (two s, 1H, CHCH_2_), 5.03 (s, 2H, O-CH_2_), 3.63 and 3.60 (two s, 3H, O-CH_3_), 3.19 and 3.16 (two s, 3H, N-CH_3_), 3.10–2.89 (m, 2H, CHCH_2_), 2.84 (s, 3H, N-CH_3_), 1.36 and 1.25 (two s, 9H, CCH_3_).

General procedure for the preparation of carbamates **13**. To the stirring mixture of magnesium ribbon (22.50 mmol) in THF (20 mL) was added dropwise the solution of 4- methoxy benzyl chloride (13.58 mmol) in THF (10 mL) and absolute ether (5 mL) at 0 °C under argon atmosphere. The mixture was stirred at room temperature for 1 h. Then, to the reaction mixture was added the solution of **12** (6.79 mmol) in THF (50 mL) at 0 °C. The mixture was stirred at room temperature for 3 h. Then, saturated ammonium chloride solution (30 mL) and H_2_O (20 mL) were added. The reaction solution was extracted with absolute ether (50 mL × 3). The combined organic layer was washed with brine (100 mL) and dried with MgSO_4_ anhydrous. The solution was filtered and evaporated under vacuum. The residue was purified by column chromatography on silica gel to give carbamates **13**.

For *tert*-butyl (1-(4-(benzyloxy)-3-methoxyphenyl)-4-(4-methoxyphenyl)-3-oxobutan-2-yl)(methyl)carbamate (**13a**): Yellow oil; Yield 85%; ^1^H NMR (400 MHz, CDCl_3_, exists as a complex mixture of two rotamers at room temperature) *δ* 7.45–7.28 (m, 5H, Ar-H), 7.12–7.04 (m, 2H, Ar-H), 6.88–6.80 (m, 2H, Ar-H), 6.77–6.74 (m, 1H, Ar-H), 6.68–6.52 (m, 2H, Ar-H), 5.11 (s, 2H, O-CH_2_), 4.78–4.70 and 4.30–4.20 (two m, 1H, CH), 3.83 (s, 3H, O-CH_3_), 3.79 and 3.78 (two s, 3H, O-CH_3_), 3.74–3.61 (m, 2H, C-CH_2_), 3.19–3.01 (m, 1H, CH_2_), 2.85–2.76 (m, 1H, CH_2_), 2.58 and 2.51 (two s, 3H, N-CH_3_), 1.43 and 1.34 (two s, 9H, C-CH_3_); ^13^C NMR (100 MHz, CDCl_3_) *δ* 206.0, 205.9, 158.7, 158.6, 155.8, 154.9, 149.6, 149.5, 146.8, 146.6, 137.3, 137.2, 131.2, 130.8, 130.5, 130.5, 128.5, 127.8, 127.3, 127.3, 125.8, 125.7, 121.2, 121.1, 114.3, 114.2, 114.1, 114.0, 112.8, 112.7, 80.8, 80.3, 71.1, 66.8, 65.9, 64.4, 56.0, 55.9, 55.3, 55.2, 46.2, 45.7, 33.4, 32.9, 32.3, 28.4, 28.2; HRMS (ESI) calcd. for C_31_H_37_NNaO_6_^+^ [M + Na]^+^ 542.2513, found 542.2515.

For *tert*-butyl (1-(3-(benzyloxy)-4-methoxyphenyl)-4-(4-methoxyphenyl)-3-oxobutan-2-yl)(methyl)carbamate (**13b**): Yellow oil; Yield 91%; ^1^H NMR (400 MHz, CDCl_3_, exists as a complex mixture of two rotamers at room temperature) *δ* 7.48–7.27 (m, 5H, Ar-H), 7.07 (dd, *J* = 8.5, 3.1 Hz, 2H, Ar-H), 6.91–6.73 (m, 3H, Ar-H), 6.71–6.58 (m, 2H, Ar-H), 5.15–5.01 (m, 2H, O-CH_2_), 4.70–4.60 and 4.19–4.09 (two m, 1H, CHCH_2_), 3.84 (s, 3H, O-CH_3_), 3.79–3.76 (m, 3H, O-CH_3_), 3.73–3.58 (m, 2H, CO-CH_2_), 3.14–3.00 (m, 1H, CHCH_2_), 2.83–2.70 (m, 1H, CHCH_2_), 2.48 and 2.43 (two s, 3H, N-CH_3_), 1.44 and 1.36 (two s, 9H, C-CH_3_); ^13^C NMR (100 MHz, CDCl_3_) *δ* 205.9, 158.7, 154.8, 148.5, 148.1, 137.1, 130.5, 130.5, 130.1, 128.6, 127.8, 127.4, 127.3, 125.8, 125.7, 121.9, 115.2, 115.0, 114.2, 114.0, 112.0, 111.9, 80.9, 80.3, 71.0, 70.9, 67.0, 64.5, 56.1, 55.3, 46.2, 45.7, 33.4, 32.8, 32.3, 28.4, 28.3; HRMS (ESI) calcd. for C_31_H_37_NNaO_6_^+^ [M + Na]^+^ 542.2513, found 542.2517.

For *tert*-butyl (1-(4-(benzyloxy)-3,5-dimethoxyphenyl)-4-(4-methoxyphenyl)-3-oxobutan-2-yl)(methyl)carbamate (**13c**): Yellow oil; Yield 85%; ^1^H NMR (400 MHz, CDCl_3_) *δ* 7.46 (d, *J* = 7.4 Hz, 2H, Ar-H), 7.35–7.26 (m, 3H, Ar-H), 7.16–7.05 (m, 2H, Ar-H), 6.92–6.81 (m, 2H, Ar-H), 6.33 and 6.26 (two s, 2H, Ar-H), 4.96 (s, 2H, O-CH_2_), 4.76–4.70 and 4.25–4.15 (two m, 1H, CH), 3.79 and 3.78 (two s, 3H, O-CH_3_), 3.76 (s, 6H, O-CH_3_), 3.74–3.61 (m, 2H, CO-CH_2_), 3.17–3.05 (m, 1H, CHCH_2_), 2.89–2.72 (m, 1H, CHCH_2_), 2.56 and 2.51 (two s, 3H, N-CH_3_), 1.46 and 1.37 (two s, 9H, C-CH_3_); ^13^C NMR (100 MHz, CDCl_3_) *δ* 205.9, 205.8, 158.8, 158.6, 155.8, 154.8, 153.6, 153.4, 138.0, 137.8, 135.5, 135.4, 134.0, 133.5, 130.6, 130.5, 128.6, 128.6, 128.2, 127.9, 127.8, 125.8, 125.7, 114.2, 114.1, 113.9, 113.6, 106.1, 106.1, 80.9, 80.4, 75.0, 67.0, 64.9, 64.3, 56.2, 56.1, 56.0, 55.3, 55.3, 55.2, 46.2, 45.7, 34.2, 33.7, 28.4, 28.3; HRMS (ESI) calcd. for C_32_H_39_NNaO_7_^+^ [M + Na]^+^ 572.2619, found 572.2614.

For *tert*-butyl (1-(4-(benzyloxy)phenyl)-4-(4-methoxyphenyl)-3-oxobutan-2-yl)(methyl)carbamate (**13d**): Light liquid; Yield 92%; ^1^H NMR (400 MHz, CDCl_3_, exists as a 1:1 mixture of two rotamers) *δ* 7.45–7.27 (m, 5H, Ar-H), 7.10–6.98 (m, 4H, Ar-H), 6.89–6.79 (m, 4H, Ar-H), 5.02 (s, 2H, O-CH_2_), 4.76–4.70 and 4.35–4.25 (two m, 1H, CH), 3.78 and 3.79 (two s, 3H, O-CH_3_), 3.73–3.61 (m, 2H, CH_2_CO), 3.15–3.07 (m, 1H, CHCH_2_), 2.87–2.75 (m, 1H, CHCH_2_), 2.58 and 2.52 (two s, 3H, N-CH_3_), 1.43 and 1.34 (two s, 9H, CCH_3_).

General procedure for the preparation of amines **15**. To the solution of absolute ether (11.4 mL) and ethanol (8.7 mL) was added dropwise acetyl chloride (7.9 mL) at -30 °C, and stirred at room temperature for 10 min. Then, carbamates **13** (1.43 mmol) was added. The reaction solution was stirred at room temperature for 1 h and concentrated. Then, absolute ether (20 mL) was added. The mixture was filtrated to give hydrochlorides **14**, which was used for the next step directly. The mixture of hydrochlorides **14** and NH_2_CN (1.32 mmol) in H_2_O (30 mL) was stirred at 90 °C for 1 h, cooled to room temperature, filtrated to give amines **15**.

For 5-(4-(benzyloxy)-3-methoxybenzyl)-4-(4-methoxybenzyl)-1-methyl-1*H*-imidazol-2-amine (**15a**): Brown powder; Yield 73%; m.p.: 95 °C (dec.); ^1^H NMR (400 MHz, CDCl_3_) *δ* 7.44–7.29 (m, 5H, Ar-H), 7.18 (d, *J* = 8.4 Hz, 2H, Ar-H), 6.79–6.76 (m, 3H, Ar-H), 6.55–6.48 (m, 2H, Ar-H), 5.64 (s, 2H, NH_2_), 5.12 (s, 2H, O-CH_2_), 3.80 (s, 2H, CH_2_), 3.77 (s, 2H, CH_2_), 3.75 (s, 3H, O-CH_3_), 3.71 (s, 3H, O-CH_3_), 3.08 (s, 3H, N-CH_3_); ^13^C NMR (100 MHz, CDCl_3_) *δ* 157.8, 149.9, 147.0, 146.8, 137.2, 132.9, 132.1, 131.8, 129.4, 128.5, 127.8, 127.3, 120.8, 119.8, 114.1, 113.8, 111.6, 71.1, 55.9, 55.2, 32.1, 29.2, 29.1; HRMS (ESI) calcd. for C_27_H_30_N_3_O_3_^+^ [M + H]^+^ 444.2282, found 444.2289.

For 5-(3-(benzyloxy)-4-methoxybenzyl)-4-(4-methoxybenzyl)-1-methyl-1*H*-imidazol-2-amine (**15b**): Brown powder; Yield 88%; m.p.: 56–58 °C; ^1^H NMR (400 MHz, CDCl_3_) *δ* 7.35–7.24 (m, 5H, Ar-H), 7.19 (d, *J* = 8.4 Hz, 2H, Ar-H), 6.85–6.75 (m, 3H, Ar-H), 6.64 (d, *J* = 7.8 Hz, 1H, Ar-H), 6.40 (s, 1H, Ar-H), 5.11 (brs, 2H, NH_2_), 4.93 (s, 2H, O-CH_2_), 3.86 (s, 3H, O-CH_3_), 3.74 (s, 2H, CH_2_), 3.73 (s, 2H, CH_2_), 3.71 (s, 3H, O-CH_3_), 2.87 (s, 3H, N-CH_3_); ^13^C NMR (100 MHz, CDCl_3_) *δ* 158.0, 148.3, 148.1, 146.9, 137.1, 132.8, 131.6, 130. 9, 129.6, 129.6, 128.6, 127.8, 127.4, 120.8, 120.4, 113.9, 111.9, 70.6, 56.1, 55.2, 32.0, 29.1, 28.9; HRMS (ESI) calcd. for C_27_H_30_N_3_O_3_^+^ [M + H]^+^ 444.2282, found 444.2291.

For 5-(4-(benzyloxy)-3,5-dimethoxybenzyl)-4-(4-methoxybenzyl)-1-methyl-1*H*-imidazol-2-amine (**15c**): Brown powder; Yield 77%; m.p.: 90–93 °C; ^1^H NMR (400 MHz, CDCl_3_) *δ* 7.46 (d, *J* = 7.0 Hz, 2H, Ar-H), 7.36–7.27 (m, 3H, Ar-H), 7.19 (d, *J* = 8.3 Hz, 2H, Ar-H), 6.78 (d, *J* = 8.3 Hz, 2H, Ar-H), 6.22 (s, 2H, Ar-H), 4.96 (s, 2H, O-CH_2_), 4.69 (brs, 2H, NH_2_), 3.81 (s, 2H, CH_2_), 3.76 (s, 2H, CH_2_), 3.75 (s, 3H, O-CH_3_), 3.67 (s, 6H, O-CH_3_), 3.07 (s, 3H, N-CH_3_); ^13^C NMR (100 MHz, CDCl_3_) *δ* 158.0, 153.7, 147.0, 137.8, 135.5, 134.2, 132.5, 129.5, 128.5, 128.1, 127.8, 120.6, 113.9, 104.9, 75.0, 56.1, 55.3, 31.9, 29.8, 29.3; HRMS (ESI) calcd. for C_28_H_32_N_3_O_4_^+^ [M + H]^+^ 474.2387, found 474.2389.

For 5-(4-(benzyloxy)benzyl)-4-(4-methoxybenzyl)-1-methyl-1*H*-imidazol-2-amine (**15d**): Brown powder; Yield 70%; m.p.: 120–123 °C; ^1^H NMR (400 MHz, CDCl_3_) *δ* 7.43–7.30 (m, 5H, Ar-H), 7.16 (d, *J* = 8.3 Hz, 2H, Ar-H), 6.98 (d, *J* = 8.3 Hz, 2H, Ar-H), 6.87 (d, *J* = 8.4 Hz, 2H, Ar-H), 6.79 (d, *J* = 8.4 Hz, 2H, Ar-H), 5.03 (s, 2H, O-CH_2_), 3.85 (s, 2H, NH_2_), 3.81 (s, 2H, CH_2_), 3.75 (s, 3H, O-CH_3_), 3.74 (s, 2H, CH_2_), 3.07 (s, 3H, N-CH_3_); HRMS (ESI) calcd. for C_26_H_28_N_3_O_2_^+^ [M + H]^+^ 414.2176, found 414.2179.

General procedure for the preparation of naamines **1a**–**d**. The mixture of amines **15** (10.45 mmol), Pd/C (10 wt%) (0.56 g), methanol (400 mL) and acetic acid (4 mL) was bubbled with hydrogen and stirred at room temperature for 24 h. Then, the mixture was filtered and concentrated. Then acetone (15 mL) was added, and filtered to give naamines **1**.

For naamine F (**1a**): Brick red powder; Yield 95%; m.p.: 163–165 °C; ^1^H NMR (400 MHz, CD_3_OD) *δ* 7.14 (d, *J* = 8.5 Hz, 2H, Ar-H), 6.77 (d, *J* = 8.5 Hz, 2H, Ar-H), 6.66 (d, *J* = 8.0 Hz, 1H, Ar-H), 6.53 (dd, *J* = 8.0, 1.4 Hz, 1H, Ar-H), 6.45 (d, *J* = 1.4 Hz, 1H, Ar-H), 3.80 (s, 2H, CH_2_), 3.73 (s, 3H, O-CH_3_), 3.72 (s, 2H, CH_2_), 3.58 (s, 3H, O-CH_3_), 3.09 (s, 3H, N-CH_3_); ^13^C NMR (100 MHz, CD_3_OD) *δ* 159.4, 149.7, 149.2, 146.1, 134.7, 132.7, 131.8, 130.5, 122.6, 121.6, 116.1, 114.7, 112.6, 56.2, 55.7, 32.8, 29.7, 29.5; HRMS (ESI) calcd. for C_20_H_24_N_3_O_3_^+^ [M + H]^+^ 354.1812, found 354.1818.

For **1b**: Brown powder; Yield 96%; m.p.: 80–83 °C; ^1^H NMR (400 MHz, CD_3_OD) *δ* 7.13 (d, *J* = 8.5 Hz, 2H, Ar-H), 6.88–6.83 (m, 3H, Ar-H), 6.61–6.58 (m, 2H, Ar-H), 3.88 (s, 2H, CH_2_), 3.84 (s, 2H, CH_2_), 3.82 (s, 3H, O-CH_3_), 3.75 (s, 3H, O-CH_3_), 3.20 (s, 3H, N-CH_3_); ^13^C NMR (100 MHz, CD_3_OD) *δ* 160.2, 148.2, 148.1, 147.8, 130.8, 130.8, 130.6, 124.0, 123.8, 120.3, 116.1, 115.3, 113.1, 56.5, 55.8, 30.1, 29.8, 28.5; HRMS (ESI) calcd. for C_20_H_24_N_3_O_3_^+^ [M + H]^+^ 354.1812, found 354.1818.

For naamine G (**1c**): Brick red powder; Yield 97%; m.p.: 172–174 °C; ^1^H NMR (400 MHz, CD_3_OD) *δ* 7.05 (d, *J* = 8.3 Hz, 2H, Ar-H), 6.67 (d, *J* = 8.3 Hz, 2H, Ar-H), 6.16 (s, 2H, Ar-H), 3.71 (s, 2H, CH_2_), 3.62 (s, 5H, CH_2_ and O-CH_3_), 3.54 (s, 6H, O-CH_3_), 3.01 (s, 3H, N-CH_3_); ^13^C NMR (100 MHz, CD_3_OD) *δ* 159.4, 149.7, 149.3, 134.9, 134.7, 132.8, 131.1, 130.5, 122.4, 114.7, 106.2, 56.6, 55.7, 32.8, 30.1, 29.5; HRMS (ESI) calcd. for C_21_H_26_N_3_O_4_^+^ [M + H]^+^ 384.1918, found 384.1919.

For naamine A (**1d**): Grey powder; Yield 87%; m.p.: 202–205 °C; ^1^H NMR (400 MHz, CD_3_OD) *δ* 7.09 (d, *J* = 8.6 Hz, 2H, Ar-H), 6.85 (d, *J* = 8.4 Hz, 2H, Ar-H), 6.77 (d, *J* = 8.6 Hz, 2H, Ar-H), 6.65 (d, *J* = 8.4 Hz, 2H, Ar-H), 3.78 (s, 2H, CH_2_), 3.73 (s, 3H, O-CH_3_), 3.71 (s, 2H, CH_2_), 3.09 (s, 3H, N-CH_3_); ^13^C NMR (100 MHz, CD_3_OD) *δ* 160.0, 157.5, 148.4, 131.5, 130.5, 130.2, 129.0, 125.2, 123.8, 116.6, 115.1, 55.7, 30.2, 29.8, 28.5; HRMS (ESI) calcd. for C_19_H_22_N_3_O_2_^+^ [M + H]^+^ 324.1707, found 324.1707.

Synthesis of 1-methylimidazolidine-2,4,5-trione (**16**). The mixture of *N*-monomethylurea (2.96 g, 0.04 mol), (COCl)_2_ (3.3 mL, 0.04 mol) in absolute ether (100 mL) was refluxed under argon for 2 h and concentrated. Then, dichloromethane (10 mL) was added, filtered to give **16** (2.87 g, 59%) as a white powder. m.p.: 146–149 °C, ^1^H NMR (400 MHz, DMSO*-d*_6_) *δ* 11.99 (s, 1H, NH), 2.92 (s, 3H, N-CH_3_).

General procedure for the preparation of naamidines **2a**–**d**. The solution of **16** (1.28 g, 10 mmol) and *N*,*O*-bis(trimethylsilyl)acetamide (2.52 g, 12.4 mmol) in acetonitrile was refluxed under argon for 45 min and concentrated to give **17**. The mixture of naamines **1** (2 mmol) and 17 in toluene (16 mL) was refluxed for 18 h. The mixture was diluted with ethyl acetate (180 mL), washed with dilute hydrochloric acid (100 mL), H_2_O (100 mL), brine (100 mL), dried with MgSO_4_ anhydrous, and evaporated under vacuum. The residue was purified by column chromatography on silica gel to give naamidines **2**.

For **2a**: Yellow powder; Yield 54%; m.p.: 162–164 °C; ^1^H NMR (400 MHz, CDCl_3_) *δ* 7.14 (d, *J* = 8.6 Hz, 2H, Ar-H), 6.84–6.77 (m, 3H, Ar-H), 6.54 (dd, *J* = 8.1, 1.8 Hz, 1H, Ar-H), 6.34 (d, *J* = 1.8 Hz, 1H, Ar-H), 3.90 (s, 4H, CH_2_), 3.77 (s, 3H, O-CH_3_), 3.65 (s, 3H, O-CH_3_), 3.49 (s, 3H, N-CH_3_), 3.18 (s, 3H, N-CH_3_); ^13^C NMR (100 MHz, CDCl_3_) *δ* 162.2, 158.2, 155.4, 146.8, 146.4, 144.6, 144.5, 135.8, 131.6, 129.3, 128.8, 126.9, 120.7, 114.5, 114.0, 110.2, 55.8, 55.3, 32.2, 30.0, 29.2, 24.7; HRMS (ESI) calcd. for C_24_H_26_N_5_O_5_^+^ [M + H]^+^ 464.1928, found 464.1932.

For naamidine B (**2b**): Yellow powder; Yield 37%; m.p.: 157–159 °C; ^1^H NMR (400 MHz, CDCl_3_) *δ* 7.12 (d, *J* = 8.5 Hz, 2H, Ar-H), 6.82 (d, *J* = 8.5 Hz, 2H, Ar-H), 6.72 (d, *J* = 8.1 Hz, 1H, Ar-H), 6.58 (s, 1H, Ar-H), 6.46 (d, *J* = 8.1 Hz, 1H, Ar-H), 3.89 (s, 2H, CH_2_), 3.86 (s, 5H, CH_2_ and O-CH_3_), 3.78 (s, 3H, O-CH_3_), 3.49 (s, 3H, N-CH_3_), 3.17 (s, 3H, N-CH_3_); ^13^C NMR (100 MHz, CDCl_3_) *δ* 162.4, 158.2, 155.8, 146.4, 145.9, 145.4, 145.2, 135.5, 131.4, 130.2, 129.3, 126.7, 119.2, 114.2, 114.0, 110.8, 56.0, 55.3, 32.2, 29.9, 28.8, 24.7; HRMS (ESI) calcd. for C_24_H_26_N_5_O_5_^+^ [M + H]^+^ 464.1928, found 464.1934.

For naamidine H (**2c**): Brown powder; Yield 81%; m.p.: 144–146 °C; ^1^H NMR (400 MHz, CDCl_3_) *δ* 7.15 (d, *J* = 7.9 Hz, 2H, Ar-H), 6.80 (d, *J* = 7.9 Hz, 2H, Ar-H), 6.15 (s, 2H, Ar-H), 3.91 (s, 2H, CH_2_), 3.89 (s, 2H, CH_2_), 3.76 (s, 3H, O-CH_3_), 3.70 (s, 6H, O-CH_3_), 3.50 (s, 3H, N-CH_3_), 3.18 (s, 3H, N-CH_3_); ^13^C NMR (100 MHz, CDCl_3_) *δ* 162.1, 158.2, 155.2, 147.2, 146.5, 144.2, 136.2, 133.5, 131.7, 129.3, 128.1, 126.7, 114.0, 104.6, 56.2, 55.3, 32.2, 30.0, 29.7, 24.7; HRMS (ESI) calcd. for C_25_H_28_N_5_O_6_^+^ [M + H]^+^ 494.2034, found 494.2030.

For naamidine A (**2d**): Yellow powder; Yield 85%; m.p.: 186–190 °C; ^1^H NMR (400 MHz, CDCl_3_) *δ* 7.11 (d, *J* = 8.5 Hz, 2H, Ar-H), 6.85–6.79 (m, 4H, Ar-H), 6.74 (d, *J* = 8.4 Hz, 2H, Ar-H), 3.88 (s, 2H, CH_2_), 3.87 (s, 2H, CH_2_), 3.77 (s, 3H, O-CH_3_), 3.37 (s, 3H, N-CH_3_), 3.17 (s, 3H, N-CH_3_); ^13^C NMR (100 MHz, CDCl_3_) *δ* 163.5, 158.3, 157.7, 155.2, 148.5, 146.4, 133.7, 130.9, 129.3, 129.0, 128.1, 126.8, 115.8, 114.1, 55.3, 31.7, 29.7, 28.6, 24.8; HRMS (ESI) calcd. for C_23_H_24_N_5_O_4_^+^ [M + H]^+^ 434.1823, found 434.1826.

General procedure for the preparation of naamines **1e**–**i**. To the solution of **15d** (4.84 mmol) and Et_3_N (9.68 mmol) in dichloromethane (120 mL) was added dropwise the solution of corresponding acyl chlorides (9.68 mmol) in dichloromethane (10 mL), and stirred at room temperature for 20 min. Then, con. HCl solution (50 mL) was added and stirred for further 20 min. The layers were separated. The organic layer was washed with saturated NaHCO_3_ (100 mL), dried over MgSO_4_ anhydrous, and evaporated to give **19**. The solution of **19** and Pd/C (10 wt%) (0.3 g) in methanol (100 mL) was bubbled H_2_ and stirred at room temperature for 24 h. The mixture was filtered and concentrated to give naamines **1e**–**i**.

For **1e**: White powder; Yield 92% for three steps; m.p.: 161–163 °C; ^1^H NMR (400 MHz, CDCl_3_) *δ* 13.41 (s, 1H), 11.03 (s, 1H), 7.13 (d, *J* = 8.1 Hz, 2H, Ar-H), 6.85–6.77 (m, 6H, Ar-H), 3.90 (s, 2H, CH_2_), 3.86 (s, 2H, CH_2_), 3.76 (s, 3H, O-CH_3_), 3.38 (s, 3H, N-CH_3_), 1.37 (s, 9H, CCH_3_); ^13^C NMR (100 MHz, DMSO*-d*_6_) *δ* 178.2, 158.1, 156.2, 136.3, 129.7, 129.5, 129.0, 127.4, 127.0, 126.3, 115.5, 114.0, 55.1, 31.5, 28.0, 26.8, 26.6; HRMS (ESI) calcd. for C_24_H_30_N_3_O_3_^+^ [M + H]^+^ 408.2282, found 408.2282.

For **1f**: White powder; Yield 87% for three steps; m.p.: 162–164 °C; ^1^H NMR (400 MHz, CDCl_3_) *δ* 7.09 (d, *J* = 8.5 Hz, 2H, Ar-H), 6.85–6.77 (m, 4H, Ar-H), 6.63 (d, *J* = 8.3 Hz, 2H, Ar-H), 3.85 (s, 2H, CH_2_), 3.79 (s, 2H, CH_2_), 3.76 (s, 3H, O-CH_3_), 3.13 (s, 3H, N-CH_3_), 2.39 (t, *J* = 7.5 Hz, 2H, COCH_2_), 1.68–1.59 (m, 2H, CH_2_), 1.30–1.25 (m, 4H, CH_2_ CH_2_), 0.84 (t, *J* = 6.6 Hz, 3H, CH_3_); ^13^C NMR (100 MHz, CDCl_3_) *δ* 158.3, 156.0, 130.5, 129.4, 128.7, 127.7, 123.1, 115.9, 114.1, 55.2, 37.7, 31.5, 30.8, 30.0, 28.2, 25.6, 22.5, 14.0; HRMS (ESI) calcd. for C_25_H_32_N_3_O_3_^+^ [M + H]^+^ 422.2438, found 422.2440.

For **1g**: White powder; Yield 68% for three steps; m.p.: 172–174 °C; ^1^H NMR (400 MHz, CDCl_3_) *δ* 9.57 (s, 1H, NH), 7.84 (d, *J* = 7.3 Hz, 2H, Ar-H), 7.48–7.35 (m, 3H, Ar-H), 7.03 (d, *J* = 8.6 Hz, 2H, Ar-H), 6.91 (d, *J* = 8.5 Hz, 2H, Ar-H), 6.85 (d, *J* = 8.6 Hz, 2H, Ar-H), 6.78 (d, *J* = 8.6 Hz, 2H, Ar-H), 3.81 (s, 3H, O-CH_3_), 3.76 (s, 4H, CH_2_), 3.07 (s, 3H, N-CH_3_); ^13^C NMR (100 MHz, DMSO*-d*_6_) *δ* 157.7, 156.0, 145.2, 144.8, 131.3, 130.9, 129.1, 128.9, 128.5, 127.3, 125.5, 121.5, 121.4, 115.3, 113.7, 55.0, 28.7, 27.8, 27.0; HRMS (ESI) calcd. for C_25_H_26_N_3_O_4_S^+^ [M + H]^+^ 464.1639, found 464.1632.

For **1h**: White powder; Yield 57% for three steps; m.p.: 207–209 °C; ^1^H NMR (400 MHz, DMSO*-d*_6_) *δ* 12.16 (s, 1H), 9.28 (s, 1H), 8.09 (s, 2H, Ar-H), 7.43 (s, 3H, Ar-H), 7.20 (d, *J* = 7.6 Hz, 2H, Ar-H), 6.91 (d, *J* = 7.8 Hz, 2H, Ar-H), 6.85 (d, *J* = 7.7 Hz, 2H, Ar-H), 6.68 (d, *J* = 7.3 Hz, 2H, Ar-H), 3.93 (s, 4H, CH_2_), 3.71 (s, 3H, O-CH_3_), 3.25 (s, 3H, N-CH_3_); ^13^C NMR (100 MHz, DMSO*-d*_6_) *δ* 157.7, 155.9, 131.4, 130.6, 129.4, 129.0, 128.4, 128.2, 127.8, 127.6, 115.4, 113.8, 55.0, 28.9, 27.2; HRMS (ESI) calcd. for C_26_H_26_N_3_O_3_^+^ [M + H]^+^ 428.1969, found 428.1971.

For **1i**: White powder; Yield 59% for three steps; m.p.: 153–154 °C; ^1^H NMR (400 MHz, CDCl_3_) *δ* 10.47 (s, 1H), 7.08 (d, *J* = 8.0 Hz, 2H, Ar-H), 6.91 (d, *J* = 7.9 Hz, 2H, Ar-H), 6.84 (d, *J* = 8.0 Hz, 2H, Ar-H), 6.80 (d, *J* = 7.9 Hz, 2H, Ar-H), 3.80 (s, 4H, CH_2_), 3.79 (s, 3H, O-CH_3_), 3.15 (s, 3H, N-CH_3_), 3.02 (s, 3H, S-CH_3_); ^13^C NMR (100 MHz, CDCl_3_) *δ* 158.7, 155.4, 145.5, 129.3, 128.9, 128.8, 127.5, 121.7, 121.2, 116.0, 114.4, 100.0, 55.4, 42.4, 29.3, 28.0; HRMS (ESI) calcd. for C_20_H_24_N_3_O_4_S^+^ [M + H]^+^ 402.1482, found 402.1481.

General procedure for the preparation of naamines **1j** and **k**. The solution of naamine A (**1d**, 3.00 mmol), benzaldehyde (12.36 mmol) and acetic acid (0.5 mL) in ethanol (120 mL) was refluxed for 12 h, evaporated part of ethanol, filtered to give **20**. The mixture of **20** and NaBH_4_ (6.5 mmol) in ethanol (100 mL) was stirred at 65 °C for 2 h, quenched with H_2_O (10 mL), and then concentrated. The residue was purified by column chromatography on silica gel to give naamines **1j** and **1k**.

For **1j**: White powder; Yield 16%; m.p.: 226 °C; ^1^H NMR (400 MHz, DMSO*-d*_6_) *δ* 9.18 (s, 1H, OH), 7.37 (d, *J* = 7.6 Hz, 2H, Ar-H), 7.33–7.19 (m, 3H, Ar-H), 7.12 (d, *J* = 8.2 Hz, 2H, Ar-H), 6.84 (d, *J* = 7.9 Hz, 2H, Ar-H), 6.77 (d, *J* = 8.2 Hz, 2H, Ar-H), 6.62 (d, *J* = 7.9 Hz, 2H, Ar-H), 5.88 (t, *J* = 5.8 Hz, 1H, NHCH_2_), 4.32 (d, *J* = 5.8 Hz, 2H, NHCH_2_), 3.71 (s, 2H, CH_2_), 3.69 (s, 3H, O-CH_3_), 3.63 (s, 2H, CH_2_), 3.03 (s, 3H, N-CH_3_); ^13^C NMR (101 MHz, DMSO*-d*_6_) *δ* 157.1, 155.5, 148.8, 140.7, 133.8, 131.5, 129.7, 129.3, 128.8, 128.0, 127.7, 126.5, 120.6, 115.1, 113.3, 54.9, 46.5, 31.9, 28.6, 27.8; HRMS (ESI) calcd. for C_26_H_28_N_3_O_2_^+^ [M + H]^+^ 414.2176, found 414.2167.

For **1k**: White powder; Yield 16%; m.p.: 226–228 °C; ^1^H NMR (400 MHz, DMSO*-d*_6_) *δ* 9.20 (s, 1H), 7.12 (d, *J* = 7.9 Hz, 2H, Ar-H), 6.83 (d, *J* = 7.8 Hz, 2H, Ar-H), 6.77 (d, *J* = 7.9 Hz, 2H, Ar-H), 6.61 (d, *J* = 7.8 Hz, 2H, Ar-H), 5.02 (t, *J* = 6.1 Hz, 1H, NH), 3.69 (s, 5H, O-CH_3_ and CH_2_), 3.62 (s, 2H, CH_2_), 3.02 (s, 3H, N-CH_3_), 2.96 (d, *J* = 6.1 Hz, 2H, NHCH_2_), 0.88 (s, 9H, CCH_3_); ^13^C NMR (100 MHz, DMSO*-d*_6_) *δ* 157.1, 155.4, 149.5, 133.8, 131.2, 129.8, 129.2, 128.8, 120.4, 115.0, 113.3, 54.9, 54.3, 31.8, 31.8, 28.6, 27.8, 27.4; HRMS (ESI) calcd. for C_24_H_32_N_3_O_2_^+^ [M + H]^+^ 394.2489, found 394.2483.

Synthesis of 4,5-bis(4-methoxybenzyl)-1,3-dimethyl-1*H*-imidazol-2(3*H*)-one (**1l**). To the solution of **1d** (0.50 g, 1.54 mmol) in THF (100 mL) was added 70% NaH (12.36 mmol) and methyliodide (12.36 mmol) successively at 0 °C under argon. Then the mixture was stirred at 70 °C for 24 h, quenched with H_2_O (10 mL), concentrated, acidified to pH 4–5 with dilute hydrochloric acid and extracted with dichloromethane. The combined organic layer was washed successively with saturated aqueous NaHCO_3_ solution (100 mL), H_2_O (100 mL), and brine (100 mL), then dried over MgSO_4_ anhydrous, filtered and concentrated. The residue was purified by column chromatography on silica gel to give **1l** (0.37 g, 68%) as a white powder. m.p.: 157–159 °C; ^1^H NMR (400 MHz, DMSO-*d_6_*) *δ* 7.08 (d, *J* = 8.6 Hz, 4H, Ar-H), 6.86 (d, *J* = 8.6 Hz, 4H, Ar-H), 3.84 (s, 4H, CH_2_), 3.72 (s, 6H, O-CH_3_), 2.89 (s, 6H, N-CH_3_); ^13^C NMR (100 MHz, CDCl_3_) *δ* 158.4, 153.8, 129.9, 128.8, 117.5, 114.1, 55.3, 28.3, 27.8; HRMS (ESI) calcd. for C_21_H_25_N_2_O_3_^+^ [M + H]^+^ 353.1860, found 353.1879.

General procedure for the preparation of naamines **1m**–**o**. The solution of **1d** (0.35 g, 1.08 mmol) and corresponding acids (2.16 mmol) in methanol (100 mL) was stirred at 50 °C for 2 h and concentrated. Then, acetone (5 mL) and petroleum ether (5 mL) were added, filtered to give **1m**–**o**.

For **1m**: White powder; Yield 98%; m.p.: 45–47 °C; ^1^H NMR (400 MHz, DMSO) *δ* 12.14 (s, 1H, OH), 9.37 (s, 1H, NH), 7.47 (s, 2H, NH_2_), 7.15 (d, *J* = 8.7 Hz, 2H, Ar-H), 6.91 (d, *J* = 8.5 Hz, 2H, Ar-H), 6.86 (d, *J* = 8.7 Hz, 2H, Ar-H), 6.69 (d, *J* = 8.5 Hz, 2H, Ar-H), 3.86 (s, 2H, CH_2_), 3.81 (s, 2H, CH_2_), 3.72 (s, 3H, O-CH_3_), 3.15 (s, 3H, N-CH_3_); ^13^C NMR (101 MHz, DMSO) *δ* 157.9, 156.1, 146.1, 130.2, 129.4, 128.9, 127.1, 122.2, 121.7, 115.4, 113.9, 55.1, 29.5, 27.9, 26.6.

For **1n**: White powder; Yield 98%; m.p.: 123–125 °C; ^1^H NMR (400 MHz, DMSO) *δ* 12.21 (s, 1H, OH), 9.36 (s, 1H, NH), 7.47 (s, 2H, NH_2_), 7.14 (d, *J* = 8.5 Hz, 2H, Ar-H), 6.92 (d, *J* = 8.3 Hz, 2H, Ar-H), 6.86 (d, *J* = 8.5 Hz, 2H, Ar-H), 6.68 (d, *J* = 8.3 Hz, 2H, Ar-H), 3.87 (s, 2H, CH_2_), 3.81 (s, 2H, CH_2_), 3.72 (s, 3H, O-CH_3_), 3.15 (s, 3H, N-CH_3_); ^13^C NMR (100 MHz, DMSO) *δ* 158.8 (q, *J* = 32.7 Hz), 158.0, 156.1, 146.2, 130.1, 129.3, 129.0, 127.1, 122.2, 121.7, 118.2, 115.4, 113.9, 55.0, 29.3, 28.0, 26.6.

For **1o**: White powder; Yield 58%; m.p.: 172–174 °C; ^1^H NMR (400 MHz, DMSO) *δ* 7.94 (d, *J* = 7.4 Hz, 2H, Ar-H), 7.54–7.40 (m, 3H, Ar-H), 7.20 (d, *J* = 8.3 Hz, 2H, Ar-H), 6.95–6.84 (m, 4H, Ar-H and NH_2_), 6.80 (d, *J* = 8.3 Hz, 2H, Ar-H), 6.66 (d, *J* = 8.2 Hz, 2H, Ar-H), 3.81 (s, 2H, CH_2_), 3.70 (s, 2H, CH_2_), 3.69 (s, 3H, O-CH_3_), 3.06 (s, 3H, N-CH_3_); ^13^C NMR (100 MHz, DMSO) *δ* 170.5, 158.1, 156.3, 148.3, 132.0, 131.4, 130.0, 129.6, 129.4, 128.7, 128.4, 121.1, 115.8, 114.1, 55.5, 30.0, 29.4, 27.5.

Synthesis of naamidine-metal complex **2e**. The mixture of **2d** (0.50 g, 1.15 mmol), ZnSO_4_·7H_2_O (69 mmol) in H_2_O (200 mL) and dichloromethane (100 mL) was stirred at room temperature for 3 h, filtered to give **2e**. Yellow powder; Yield 38%; m.p.: 185–187 °C; ^1^H NMR (400 MHz, DMSO*-d*_6_) *δ* 9.30 (s, 2H, OH), 6.95 (d, *J* = 8.2 Hz, 4H, Ar-H), 6.69 (d, *J* = 8.2 Hz, 4H, Ar-H), 6.50 (s, 8H, Ar-H), 4.04–3.90 (m, 4H, CH_2_), 3.82 (d, *J* = 16.7 Hz, 2H, CH_2_), 3.62 (s, 6H, O-CH_3_), 3.57 (s, 6H, N-CH_3_), 3.26 (d, *J* = 16.7 Hz, 2H, CH_2_), 2.80 (s, 6H, N-CH_3_); ^13^C NMR (100 MHz, DMSO*-d*_6_) *δ* 163.7, 160.5, 157.3, 155.9, 153.2, 146.8, 132.0, 129.9, 129.0, 128.4, 128.3, 127.7, 115.4, 113.0, 54.7, 30.2, 29.9, 27.4, 24.0; HRMS (ESI) calcd. for C_46_H_45_N_10_O_8_Zn^+^ [M + H]^+^ 929.2708, found 929.2709.

### 3.2. Biological Assay

Each bioassay was repeated three times at 25 ± 1 °C. Activity results were estimated according to a percentage scale of 0–100 (0: no activity; 100: total kill).

Detailed bioassay procedures for the anti-TMV [15] and fungicidal [24] activity were described in our published literature.

## 4. Conclusions

Marine natural products naamines A, F and G, naamidines A, B and H, and various derivatives were synthesized, and their activities against a plant virus and phytopathogenic fungi were evaluated for the first time. The introduction of a benzyl group on the aromatic ring was favorable for activity. Derivative **15d**, which had higher antiviral activity than ribavirin in all the assays, emerged as a new lead compound for antiviral research. Salification and derivatization of amino-group attenuated activity. Assays on 14 kinds of phytopathogenic fungi revealed that these compounds displayed very good fungicidal activity at 50 μg/mL. Again, **15d** emerged as a new lead compound for fungicidal research, owing to its broad-spectrum fungicidal activity. We expect that the results of our study will provide a basis for the development of these alkaloids as antiviral and fungicidal agents.

## Figures and Tables

**Table 1:** Activities of **1a**–**o**, **2a**–**e** and **15a**–**d** against tobacco mosaic virus (TMV) at 500 µg/mL.

Compound	In Vitro Inhibition Rate (%) ^a^	In Vivo		
		Inactivation Effect (%) ^a^	Curative Effect (%) ^a^	Protection Effect (%) ^a^
**1a**	26 ± 2	32 ± 2	35 ± 1	28 ± 2
**1b**	32 ± 1	31 ± 1	27 ± 2	35 ± 1
**1c**	0	7 ± 2	0	0
**1d**	0	14 ± 2	0	6 ± 2
**1e**	9 ± 2	22 ± 3	12 ± 2	16 ± 2
**1f**	0	14 ± 2	0	18 ± 2
**1g**	18 ± 1	34 ± 2	29 ± 1	37 ± 2
**1h**	0	18 ± 1	10 ± 2	0
**1i**	17 ± 2	15 ± 2	0	12 ± 2
**1j**	22 ± 1	26 ± 2	29 ± 3	17 ± 2
**1k**	10 ± 2	17 ± 2	22 ± 1	12 ± 2
**1l**	0	0	10 ± 2	0
**1m**	12 ± 1	32 ± 2	24 ± 1	22 ± 2
**1n**	18 ± 3	23 ± 2	12 ± 1	20 ± 2
**1o**	16 ± 2	14 ± 2	9 ± 2	0
**2a**	0	31 ± 2	20 ± 1	22 ± 2
**2b**	21 ± 1	0	12 ± 2	16 ± 2
**2c**	0	8 ± 2	10 ± 1	0
**2d**	12 ± 2	0	0	10 ± 2
**2e**	25 ± 2	34 ± 2	37 ± 1	29 ± 2
**15a**	23 ± 1	35 ± 2	29 ± 2	32 ± 2
**15b**	36 ± 2	30 ± 2	33 ± 2	27 ± 2
**15c**	12 ± 2	21 ± 2	16 ± 1	22 ± 2
**15d**	43 ± 2	46 ± 2	49 ± 2	41 ± 2
Tween-80	0	0	0	0
**Ribavirin**	39 ± 1	32 ± 1	35 ± 2	34 ± 1

^a^ Average of three replicates; all results are expressed as means ± SD.

**Table 2:** In vitro activities of **1a**–**o**, **2a**–**e** and **15a**–**d** against 14 kinds of fungi.

Compound	Fungicidal Activity ^a^ (%)/50 μg/mL													
	F.C ^c^	C.H	P.P	R.C	B.M	W.A	F.M	A.S	F.G	P.I	P.C	S.S	R.S	B.C
**1a**	12 ± 1	30 ± 2	11 ± 1	21 ± 2	20 ± 2	28 ± 1	22 ± 2	25 ± 3	28 ± 2	17 ± 2	9 ± 1	38 ± 2	40 ± 1	38 ± 2
**1b**	12 ± 2	30 ± 3	67 ± 2	21 ± 1	28 ± 2	28 ± 1	19 ± 2	38 ± 2	36 ± 1	25 ± 2	17 ± 2	30 ± 3	47 ± 2	24 ± 1
**1c**	20 ± 1	27 ± 2	0	18 ± 2	23 ± 1	33 ± 2	26 ± 1	50 ± 2	24 ± 2	17 ± 1	9 ± 2	27 ± 1	20 ± 2	24 ± 1
**1d**	32 ± 2	47 ± 1	44 ± 2	70 ± 1	35 ± 2	47 ± 3	37 ± 2	44 ± 1	56 ± 2	25 ± 2	22 ± 1	38 ± 2	27 ± 1	27 ± 2
**1e**	7 ± 2	27 ± 2	30 ± 2	11 ± 2	20 ± 2	39 ± 2	11 ± 2	13 ± 2	56 ± 2	8 ± 2	35 ± 2	33 ± 1	33 ± 2	29 ± 2
**1f**	29 ± 1	17 ± 2	9 ± 1	34 ± 2	28 ± 1	28 ± 2	30 ± 1	25 ± 2	44 ± 1	4 ± 2	22 ± 3	30 ± 2	40 ± 2	29 ± 3
**1g**	37 ± 2	27 ± 1	41 ± 2	47 ± 1	45 ± 2	33 ± 3	22 ± 2	19 ± 1	28 ± 2	8 ± 2	8 ± 1	27 ± 2	40 ± 2	38 ± 1
**1h**	17 ± 1	30 ± 2	20 ± 1	20 ± 2	18 ± 2	28 ± 1	19 ± 2	25 ± 2	44 ± 1	8 ± 2	13 ± 2	23 ± 2	20 ± 3	21 ± 2
**1i**	24 ± 2	20 ± 3	4 ± 2	28 ± 2	23 ± 3	28 ± 2	22 ± 2	13 ± 1	52 ± 2	4 ± 1	9 ± 2	8 ± 1	13 ± 2	38 ± 3
**1j**	27 ± 1	40 ± 2	22 ± 1	70 ± 2	30 ± 2	33 ± 1	48 ± 2	19 ± 2	56 ± 1	8 ± 2	9 ± 2	33 ± 3	27 ± 2	29 ± 1
**1k**	20 ± 2	33 ± 1	54 ± 2	48 ± 2	33 ± 1	33 ± 2	41 ± 1	19 ± 2	44 ± 2	8 ± 1	30 ± 2	33 ± 1	27 ± 2	24 ± 2
**1l**	15 ± 1	40 ± 2	0	21 ± 3	23 ± 2	25 ± 1	15 ± 2	38 ± 1	32 ± 2	8 ± 2	4 ± 1	56 ± 2	47 ± 1	53 ± 2
**1m**	22 ± 2	47 ± 1	26 ± 2	72 ± 2	33 ± 3	44 ± 2	30 ± 2	38 ± 1	36 ± 2	25 ± 1	9 ± 2	33 ± 1	20 ± 2	12 ± 1
**1n**	22 ± 1	23 ± 2	22 ± 1	42 ± 2	33 ± 3	47 ± 2	30 ± 1	38 ± 2	32 ± 1	8 ± 2	4 ± 2	67 ± 1	27 ± 2	56 ± 1
**1o**	22 ± 2	30 ± 1	22 ± 2	25 ± 1	30 ± 2	42 ± 2	22 ± 1	31 ± 2	40 ± 2	17 ± 1	4 ± 2	35 ± 2	20 ± 3	21 ± 2
**2a**	7 ± 1	27 ± 2	28 ± 3	16 ± 2	15 ± 1	25 ± 2	15 ± 1	50 ± 2	68 ± 1	17 ± 2	17 ± 2	11 ± 1	13 ± 2	47 ± 3
**2b**	12 ± 2	23 ± 3	7 ± 2	20 ± 1	15 ± 2	28 ± 1	11 ± 2	19 ± 2	24 ± 1	13 ± 2	13 ± 1	18 ± 2	17 ± 1	24 ± 2
**2c**	7 ± 1	67 ± 2	22 ± 2	62 ± 3	35 ± 2	25 ± 1	15 ± 2	38 ± 2	56 ± 1	8 ± 2	30 ± 3	21 ± 2	20 ± 2	47 ± 3
**2d**	10 ± 2	23 ± 3	33 ± 2	14 ± 2	15 ± 2	14 ± 2	15 ± 1	38 ± 1	68 ± 2	50 ± 1	44 ± 2	23 ± 1	47 ± 2	53 ± 2
**2e**	12 ± 1	27 ± 2	4 ± 1	14 ± 2	10 ± 1	25 ± 2	22 ± 2	6 ± 2	20 ± 1	4 ± 2	9 ± 1	8 ± 2	40 ± 1	12 ± 2
**15a**	17 ± 1	40 ± 2	57 ± 1	28 ± 2	23 ± 1	28 ± 2	26 ± 1	19 ± 2	28 ± 1	25 ± 2	57 ± 1	24 ± 2	10 ± 1	24 ± 2
**15b**	12 ± 2	30 ± 2	0	21 ± 1	23 ± 2	42 ± 2	30 ± 2	38 ± 3	32 ± 2	42 ± 2	52 ± 2	38 ± 3	47 ± 2	50 ± 1
**15c**	12 ± 1	37 ± 2	65 ± 1	32 ± 2	35 ± 1	39 ± 2	37 ± 1	44 ± 2	32 ± 3	33 ± 2	65 ± 1	36 ± 2	47 ± 1	21 ± 2
**15d**	17 ± 2	50 ± 1	54 ± 2	48 ± 1	33 ± 2	42 ± 1	33 ± 2	50 ± 2	36 ± 1	58 ± 2	74 ± 3	61 ± 2	63 ± 1	50 ± 2
**water**	0	0	0	0	0	0	0	0	0	0	0	0	0	0
chlorothalonil ^b^	100	73 ± 2	100	73 ± 1	<50	100	<50	100	100	91 ± 1	91 ± 2	86 ± 3	100	100
carbendazim ^b^	<50	<50	<50	<50	100	<50	100	<50	100	100	100	100	100	<50

^a^ Average of three replicates. ^b^ The commercial agricultural fungicides chlorothalonil and carbendazim were used for comparison of antifungal activity. ^c^ F.C, *Fusarium oxysporium f.* sp. *cucumeris*; C.H, *Cercospora arachidicola Hori*; P.P, *Physalospora piricola*; R.C, *Rhizoctonia cerealis*; B.M, *Bipolaris maydis*; W.A, *watermelon anthracnose*; F.M, *Fusarium moniliforme*; A.S, *Alternaria solani*; F.G, *Fusarium graminearum*; P.I,*Phytophthora infestans*; P.C, *Phytophthora capsici*; S.S, *Sclerotinia sclerotiorum*; R.S, *Rhizoctonia solani*; B.C, *Botrytis cinerea.*

**Table 3:** In vivo fungicidal activities of **1a**–**o**, **2a**–**e** and **15a**–**d** against 6 kinds of Fungi.

Compound	Inhibition Rate (%) ^b^/200 μg/mL					
	S.S ^a^	R.C	B.C	P.C	C.C	B.G
**1a**	25 ± 2	28 ± 1	27 ± 2	15 ± 2	36 ± 1	0
**1b**	15 ± 2	28 ± 2	9 ± 2	20 ± 1	25 ± 2	0
**1c**	13 ± 1	17 ± 2	9 ± 2	10 ± 2	36 ± 2	0
**1d**	10 ± 2	11 ± 2	18 ± 2	20 ± 1	15 ± 2	0
**1e**	10 ± 2	17 ± 1	9 ± 2	30 ± 2	18 ± 2	0
**1f**	13 ± 2	28 ± 2	9 ± 1	25 ± 2	32 ± 1	0
**1g**	10 ± 1	32 ± 2	18 ± 2	15 ± 2	36 ± 2	0
**1h**	8 ± 2	11 ± 1	18 ± 2	15 ± 2	15 ± 1	0
**1i**	8 ± 2	6 ± 2	27 ± 2	15 ± 1	11 ± 2	0
**1j**	19 ± 2	11 ± 1	27 ± 2	10 ± 2	11 ± 2	0
**1k**	21 ± 2	22 ± 2	9 ± 1	30 ± 2	18 ± 2	0
**1l**	27 ± 1	17 ± 2	27 ± 1	5 ± 2	25 ± 2	0
**1m**	10 ± 2	6 ± 2	9 ± 2	10 ± 1	15 ± 2	0
**1n**	27 ± 1	6 ± 2	18 ± 1	5 ± 2	21 ± 2	0
**1o**	27 ± 2	28 ± 2	9 ± 2	10 ± 2	15 ± 1	0
**2a**	8 ± 2	28 ± 1	27 ± 2	10 ± 2	25 ± 2	0
**2b**	8 ± 2	11 ± 2	9 ± 2	15 ± 2	11 ± 2	0
**2c**	19 ± 2	17 ± 1	18 ± 2	15 ± 1	32 ± 2	0
**2d**	10 ± 1	17 ± 2	18 ± 1	20 ± 2	18 ± 1	0
**2e**	8 ± 2	22 ± 1	9 ± 2	10 ± 1	32 ± 2	0
**15a**	27 ± 2	17 ± 1	9 ± 2	25 ± 1	15 ± 2	0
**15b**	27 ± 1	32 ± 2	27 ± 1	40 ± 2	25 ± 1	0
**15c**	19 ± 2	25 ± 1	18 ± 2	40 ± 1	36 ± 2	0
**15d**	35 ± 1	32 ± 2	27 ± 1	40 ± 2	15 ± 1	0
**water**	0	0	0	0	0	0
azoxystrobin ^c^	100	100	100	83 ± 2	81 ± 1	82 ± 2

^a^ S.S, Sclerotinia sclerotiorum (rape-protection); R.C, Rhizoctonia cerealis; B.C, Botrytis cinerea. (cucumber-protection); P.C, Phytophthora capsici; C.C, Corynespora cassiicola (cucumber-protection); B.G, *Blum eria graminis f.* sp. tritici (wheat-protection). ^b^ Average of five replicates. ^c^ The dilution of azoxystrobin is 1000 times.

## Supplementary Materials

The following are available online at http://www.mdpi.com/1660-3397/16/9/311/s1, ^1^H and ^13^C NMR, HRMS spectra of compounds **1**, **2** and **15**.
