# Supplementary material for: Naamines and Naamidines as Novel Agents against a Plant Virus and Phytopathogenic Fungi
Source: Mar Drugs. 2018 Sep 3;16(9):311. doi: 10.3390/md16090311 (PMC6164833; doi:10.3390/md16090311)
Supplement: Supplementary file 1 [file marinedrugs-16-00311-s001.pdf]

Electronic supplementary information (ESI)

Naamines and Naamidines as Novel Agents against a Plant Virus and Phytopathogenic Fungi

Pengbin Guo [1], Gang Li [1], Yuxiu Liu [1], Aidang Lu [1], Ziwen Wang [2,4,*] and Qingmin Wang [1,3,*]

1 State Key Laboratory of Elemento-Organic Chemistry, Research Institute of Elemento-Organic Chemistry, College of Chemistry, Nankai University, Tianjin 300071, China; 13212007181@163.com (P.G.); 2120150654@mail.nankai.edu.cn (G.L.); liuyuxiu@nankai.edu.cn (Y.L.); aidang_lu@163.com (A.L.)

2 Tianjin Key Laboratory of Structure and Performance for Functional Molecules, College of Chemistry, Tianjin Normal University, Tianjin 300387, China

3 Collaborative Innovation Center of Chemical Science and Engineering (Tianjin), Tianjin 300071, China

4 Key Laboratory of Inorganic-Organic Hybrid Functional Materials Chemistry (Tianjin Normal University), Ministry of Education, Tianjin 300387, China

* Correspondence: hxxywzw@tjnu.edu.cn(Z.W.); wangqm@nankai.edu.cn (Q.W.); Tel.: +86-22-2376-6531(Z.W.); +86-22-2350-3952(Q.W.)

Contents

^{1}H NMR and ^{13}C NMR spectra of **1a** ········ S4

HRMS spectrum of **1a** and ^{1}H NMR spectrum of **1b** ········ S5

^{13}C NMR and HRMS spectra of **1b** ········ S6

^{1}H NMR and ^{13}C NMR spectra of **1c** ········ S7

HRMS spectrum of **1c** and ^{1}H NMR spectrum of **1d** ········ S8

^{13}C NMR and HRMS spectra of **1d** ········ S9

^{1}H NMR and ^{13}C NMR spectra of **1e** ········ S10

HRMS spectrum of **1e** and ^{1}H NMR spectrum of **1f** ········ S11

^{13}C NMR and HRMS spectra of **1f** ········ S12

^{1}H NMR and ^{13}C NMR spectra of **1g** ········ S13

HRMS spectrum of **1g** and ^{1}H NMR spectrum of **1h** ········ S14

^{13}C NMR and HRMS spectra of **1h** ········ S15

^{1}H NMR and ^{13}C NMR spectra of **1i** ········ S16

HRMS spectrum of **1i** and ^{1}H NMR spectrum of **1j** ········ S17

^{13}C NMR and HRMS spectra of **1j** ········ S18

^{1}H NMR and ^{13}C NMR spectra of **1k** ········ S19

HRMS spectrum of **1k** and ^{1}H NMR spectrum of **1l** ········ S20

^{13}C NMR and HRMS spectra of **1l** ········ S21

^{1}H NMR and ^{13}C NMR spectra of **1m** S22

^{1}H NMR and ^{13}C NMR spectra of **1n** S23

^{1}H NMR and ^{13}C NMR spectra of **1o** S24

^{1}H NMR and ^{13}C NMR spectra of **2a** S25

HRMS spectrum of **2a** and ^{1}H NMR spectrum of **2b** S26

^{13}C NMR and HRMS spectra of **2b** S27

^{1}H NMR and ^{13}C NMR spectra of **2c** S28

HRMS spectrum of **2c** and ^{1}H NMR spectrum of **2d** S29

^{13}C NMR and HRMS spectra of **2d** S30

^{1}H NMR and ^{13}C NMR spectra of **2e** S31

HRMS spectrum of **2e** S32

^{1}H NMR spectra of **4a** and **4b** S33

^{1}H NMR spectra of **4c** and **5a** S34

^{1}H NMR spectra of **5b** and **5c** S35

^{13}C NMR and HRMS spectra of **5c** S36

^{1}H NMR spectra of **6a** and **6b** S37

^{1}H NMR and ^{13}C NMR spectra of **6c** S38

HRMS spectrum of **6c** and ^{1}H NMR spectrum of **7a** S39

^{1}H NMR spectra of **7b** and **7c** S40

^{13}C NMR and HRMS spectra of **7c** S41

^{1}H NMR spectra of **10a** and **10b** S42

^{1}H NMR spectra of **10c** and **10d** S43

^{1}H NMR and ^{13}C NMR spectra of **11a** S44

HRMS spectrum of **11a** and ^{1}H NMR spectrum of **11b** S45

^{13}C NMR and HRMS spectra of **11b** S46

^{1}H NMR and ^{13}C NMR spectra of **11c** S47

HRMS spectrum of 11c and ^{1}H NMR spectrum of 11d S48

^{1}H NMR and ^{13}C NMR spectra of **12a** S49

HRMS spectrum of **12a** and ^{1}H NMR spectrum of **12b** S50

^{13}C NMR and HRMS spectra of **12b** S51

^{1}H NMR and ^{13}C NMR spectra of **12c**······S52

HRMS spectrum of **12c** and ^{1}H NMR spectrum of **12d**······S53

^{1}H NMR and ^{13}C NMR spectra of **13a** ······S54

HRMS spectrum of **13a** and ^{1}H NMR spectrum of **13b**······S55

^{13}C NMR and HRMS spectra of **13b**······S56

^{1}H NMR and ^{13}C NMR spectra of **13c**······S57

HRMS spectrum of **13c** and ^{1}H NMR spectrum of **13d**······S58

^{1}H NMR and ^{13}C NMR spectra of **15a** ······S59

HRMS spectrum of **15a** and ^{1}H NMR spectrum of **15b**······S60

^{13}C NMR and HRMS spectra of **15b**······S61

^{1}H NMR and ^{13}C NMR spectra of **15c**······S62

HRMS spectrum of **15c** and ^{1}H NMR spectrum of **15d**······S63

HRMS spectrum of **15d** and ^{1}H NMR spectrum of **16** ······S64

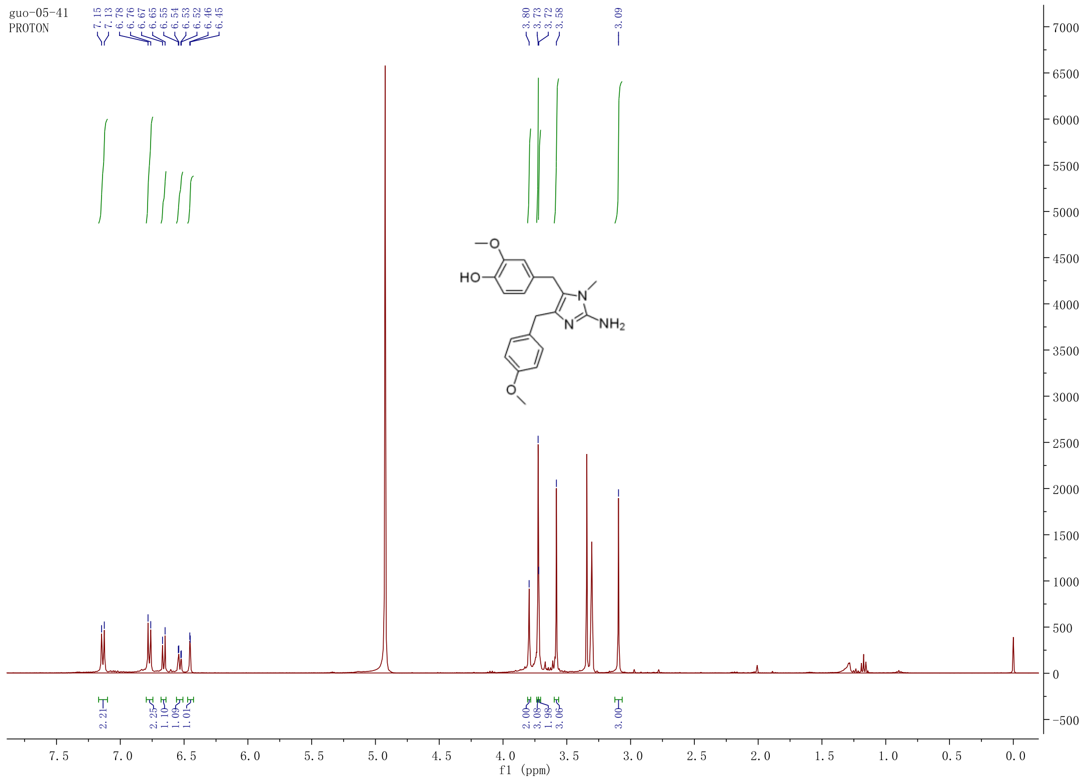

^{1}H NMR spectrum of **1a**

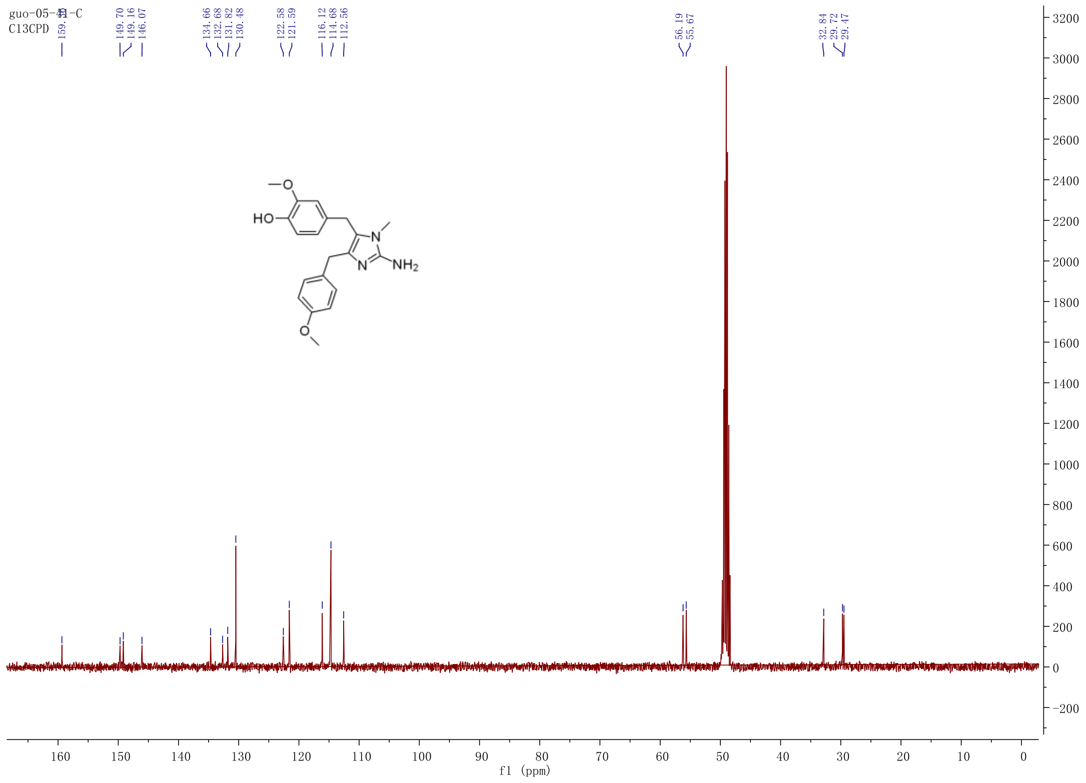

^{13}C NMR spectrum of **1a**

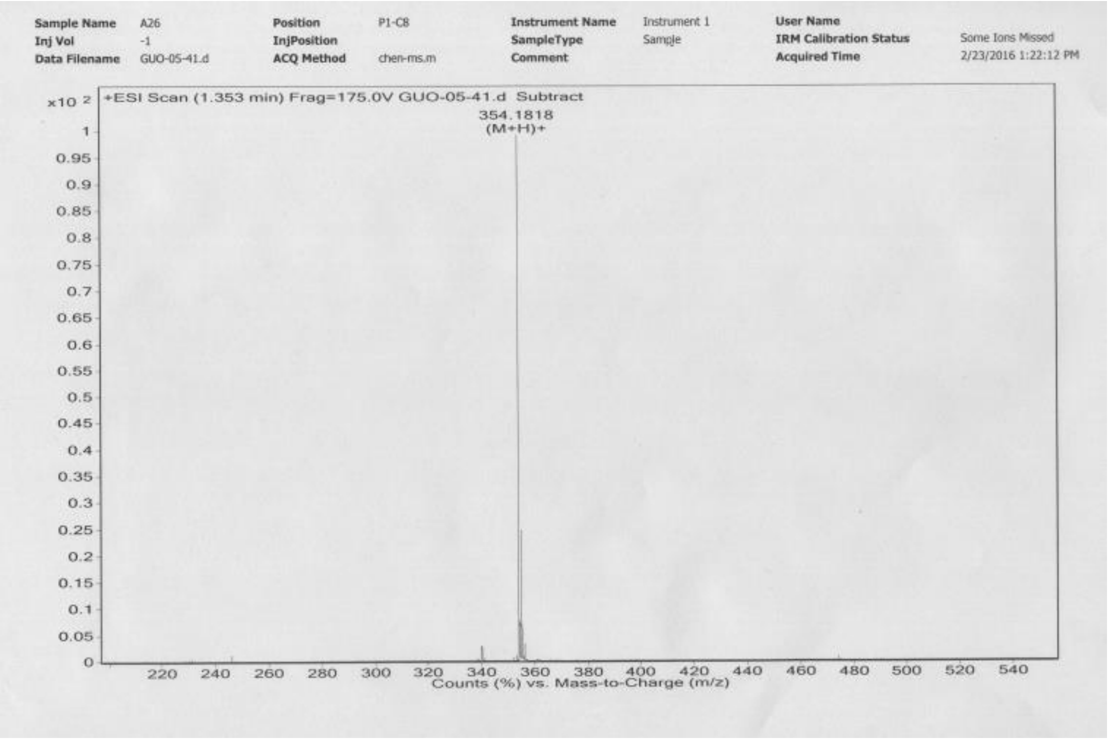

HRMS spectrum of **1a**

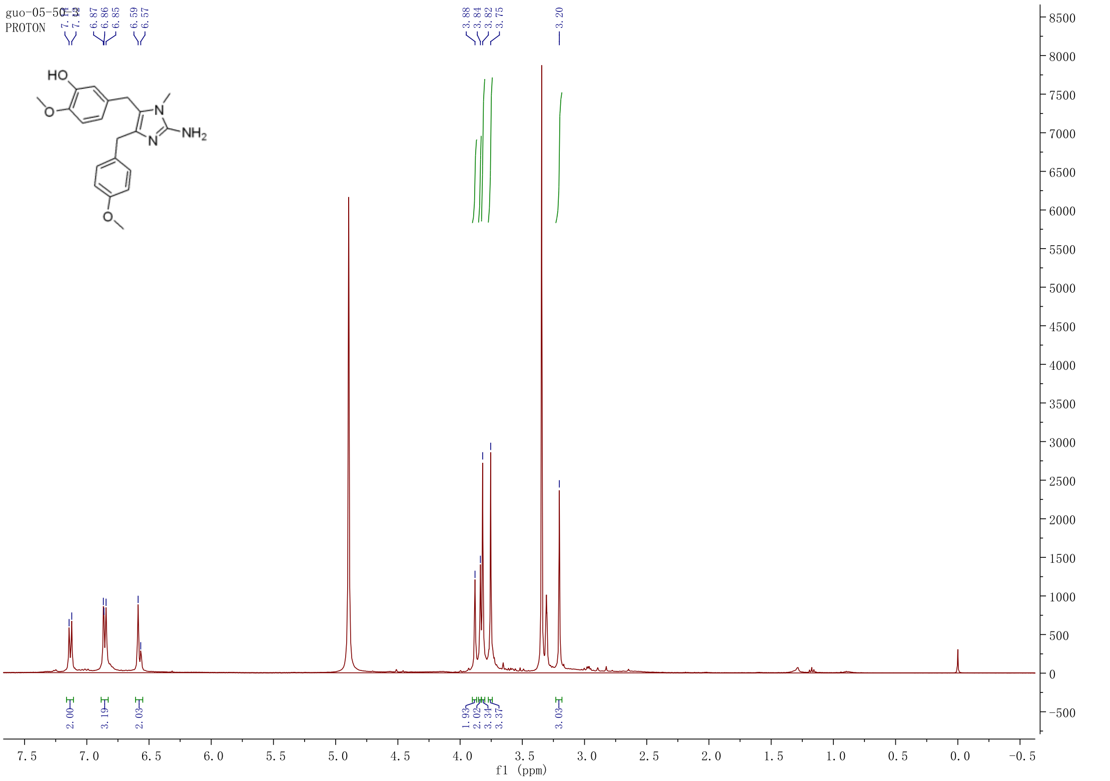

^{1}H NMR spectrum of **1b**

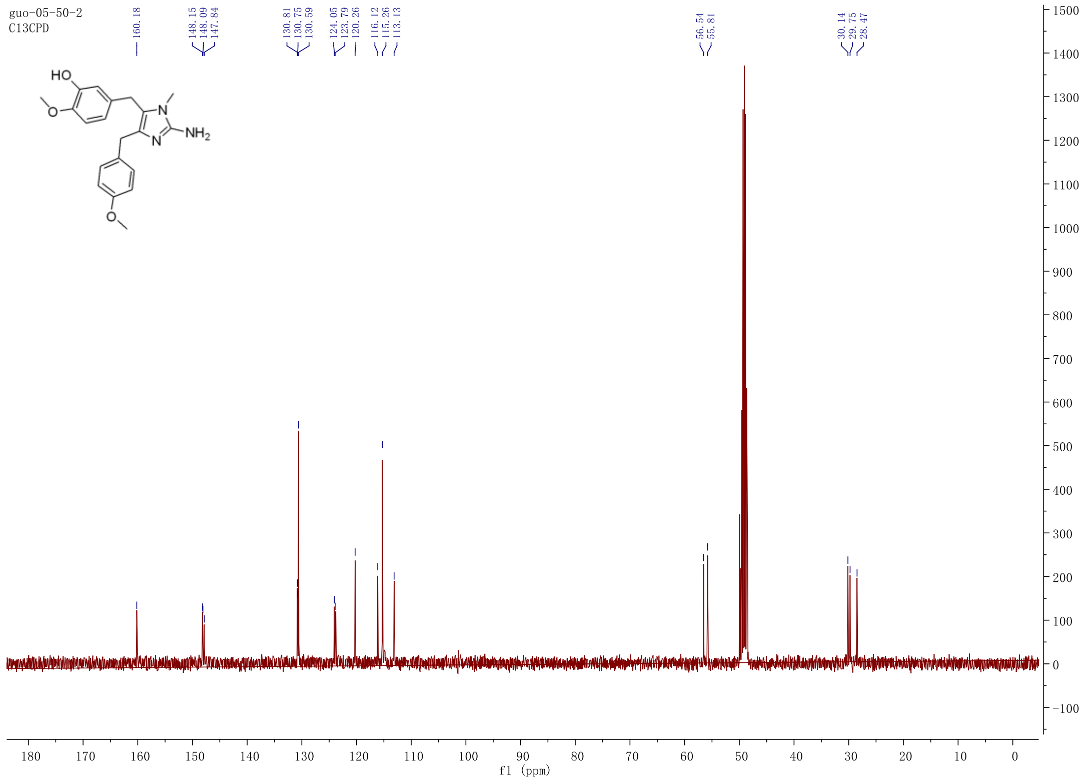

^{13}C NMR spectrum of **1b**

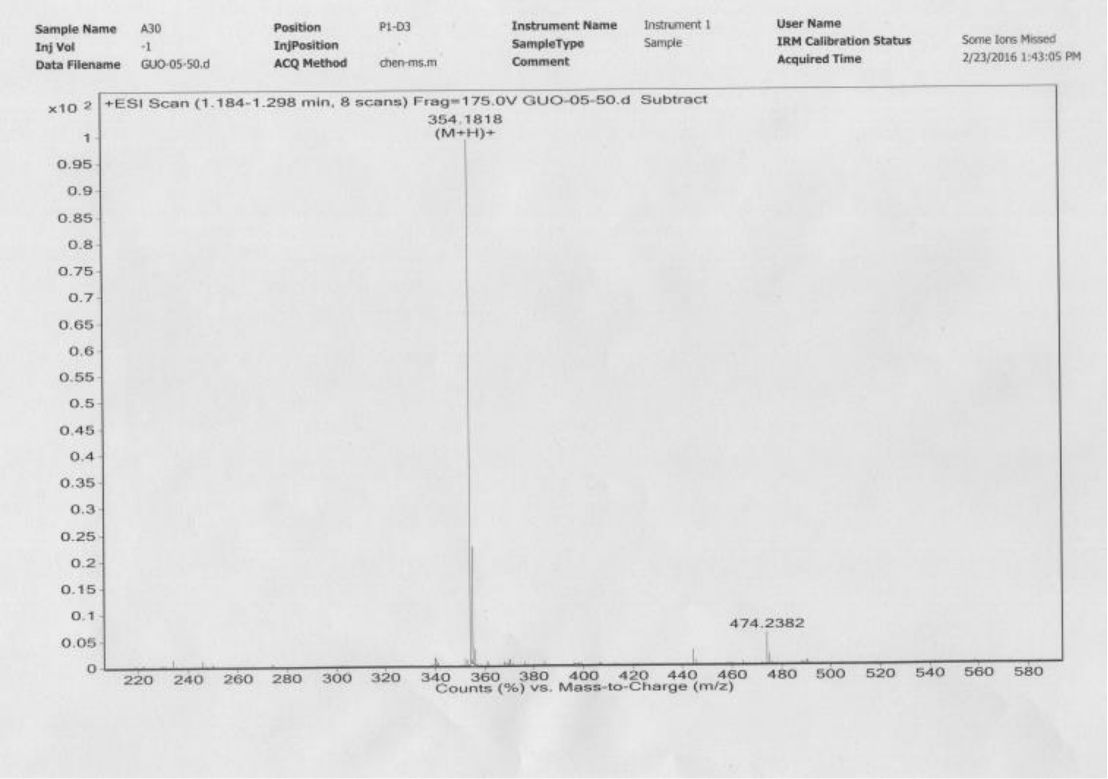

HRMS spectrum of **1b**

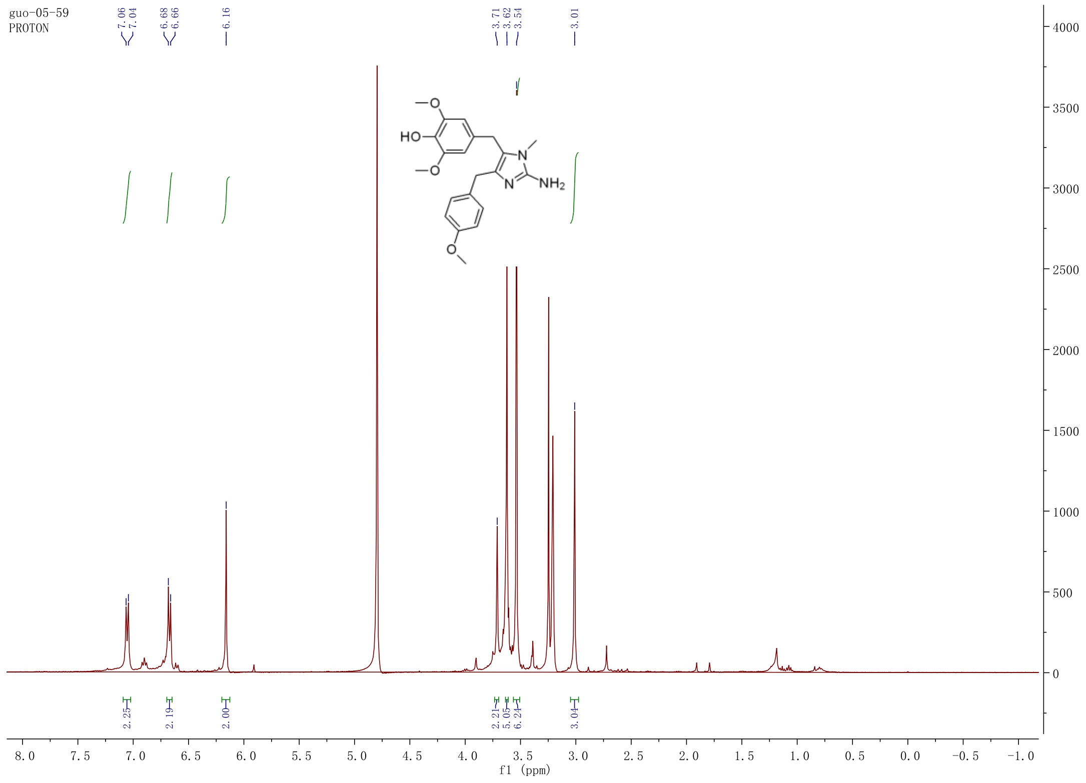

^{1}H NMR spectrum of **1c**

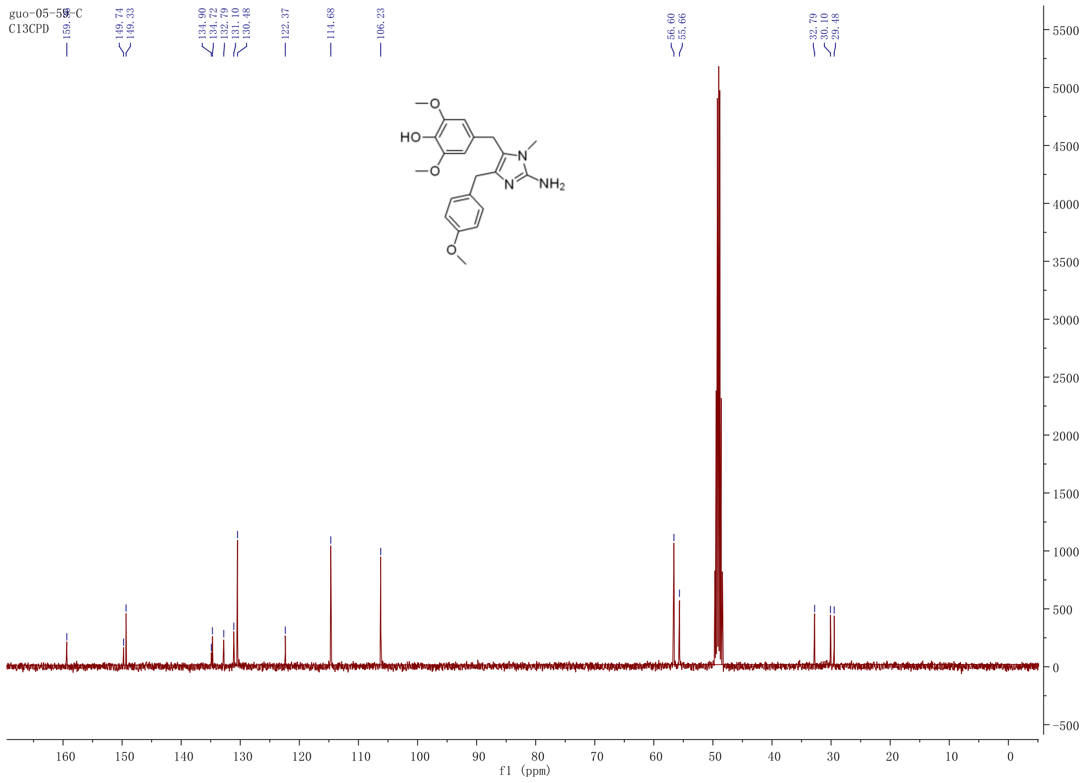

^{13}C NMR spectrum of **1c**

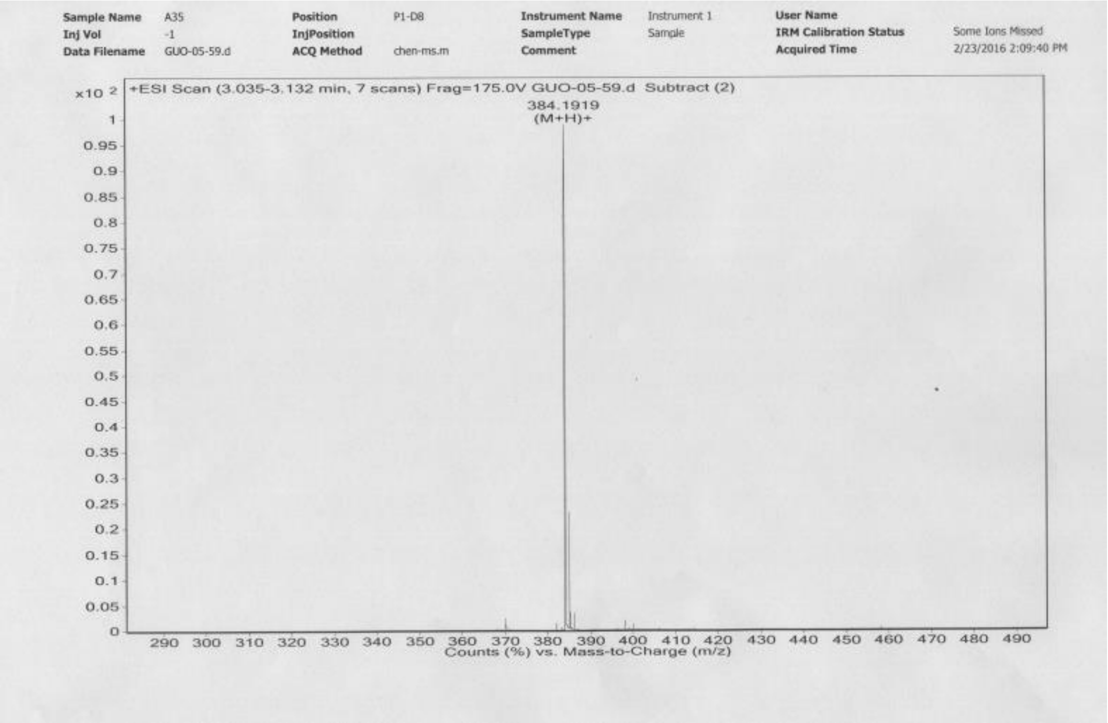

HRMS spectrum of **1c**

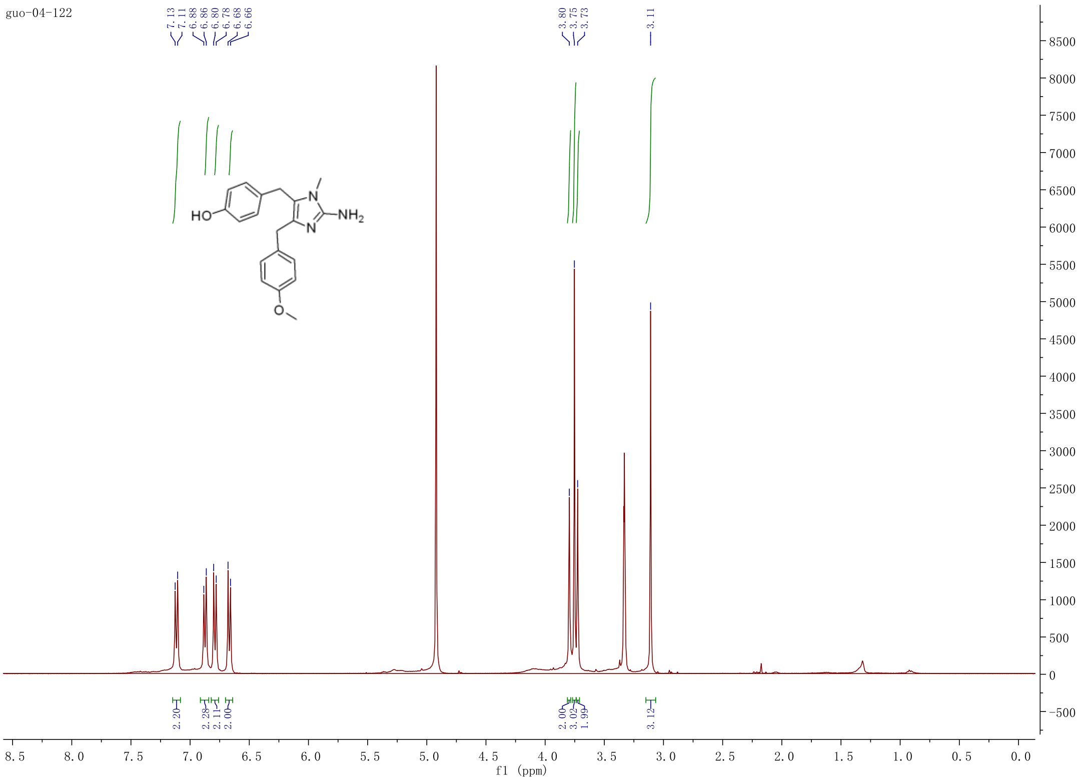

^{1}H NMR spectrum of **1d**

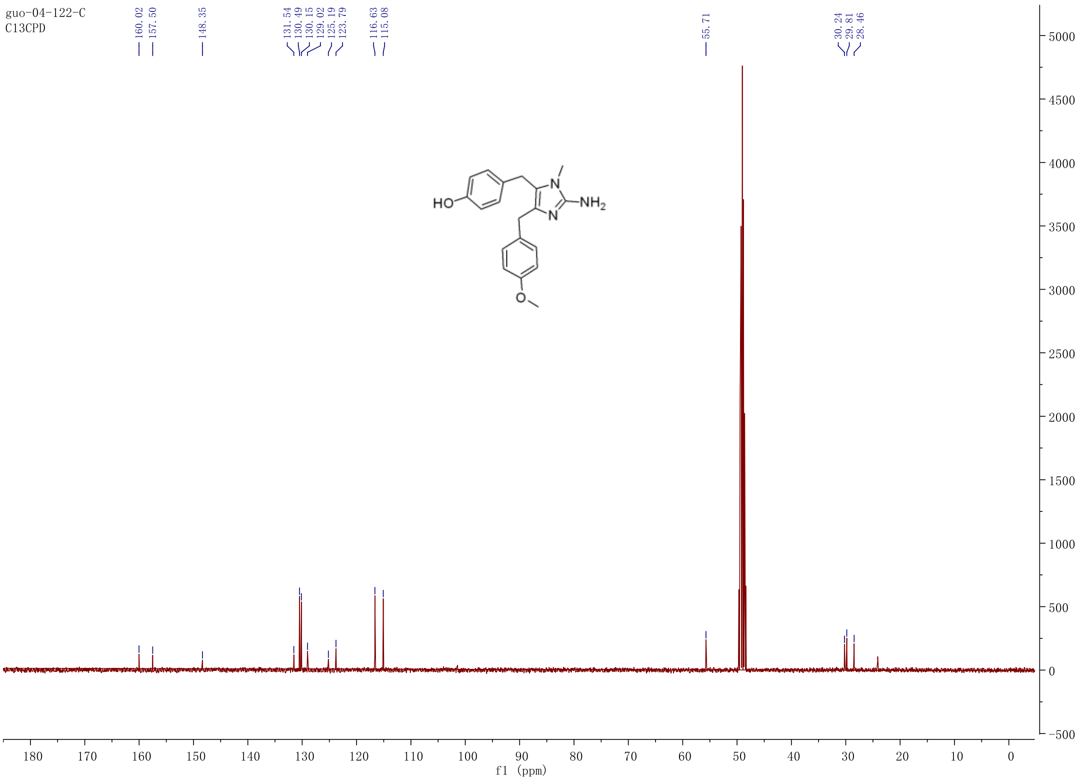

^{13}C NMR spectrum of **1d**

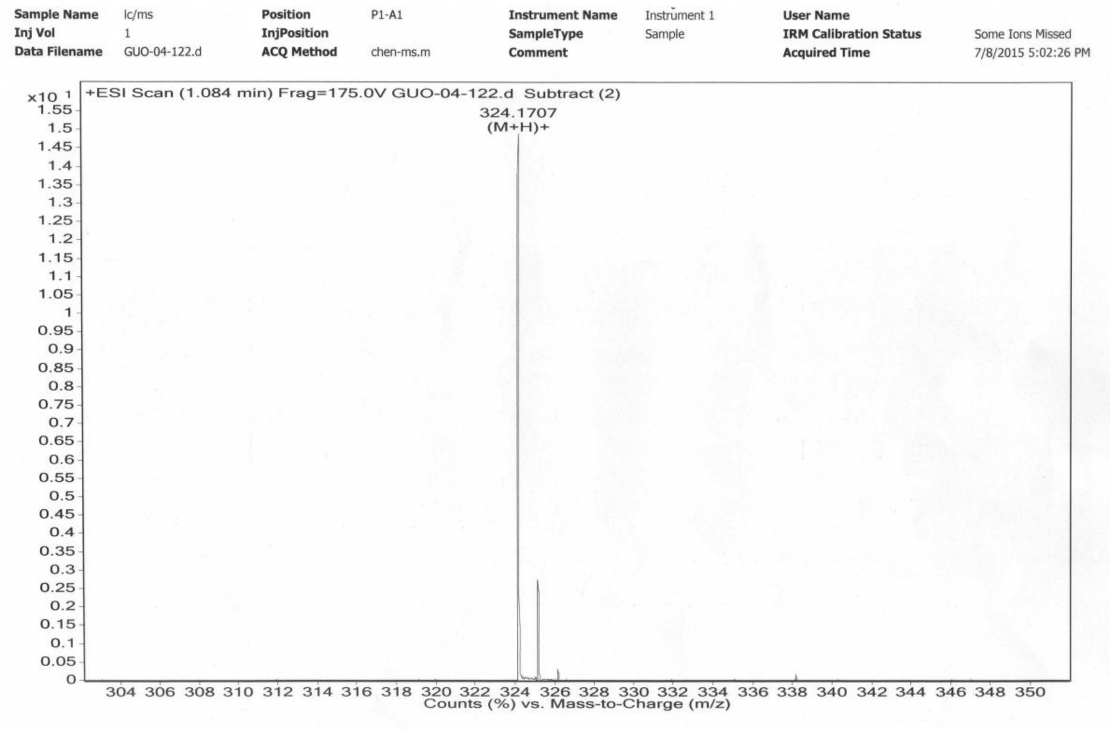

HRMS spectrum of **1d**

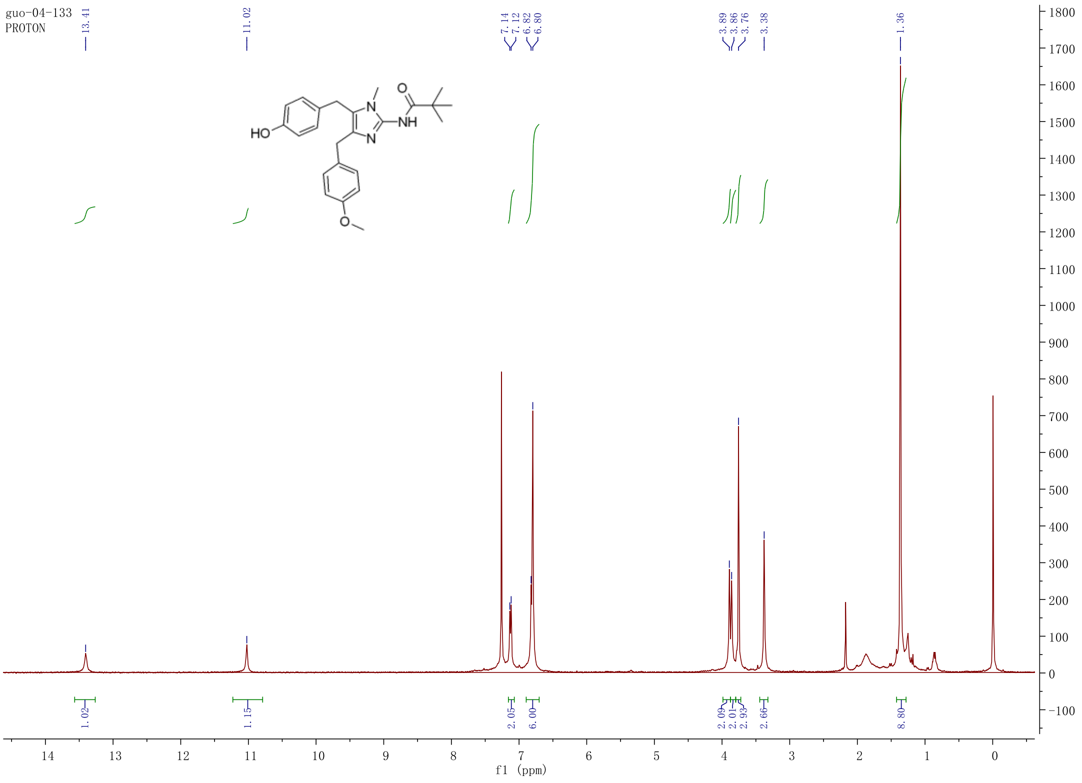

^{1}H NMR spectrum of **1e**

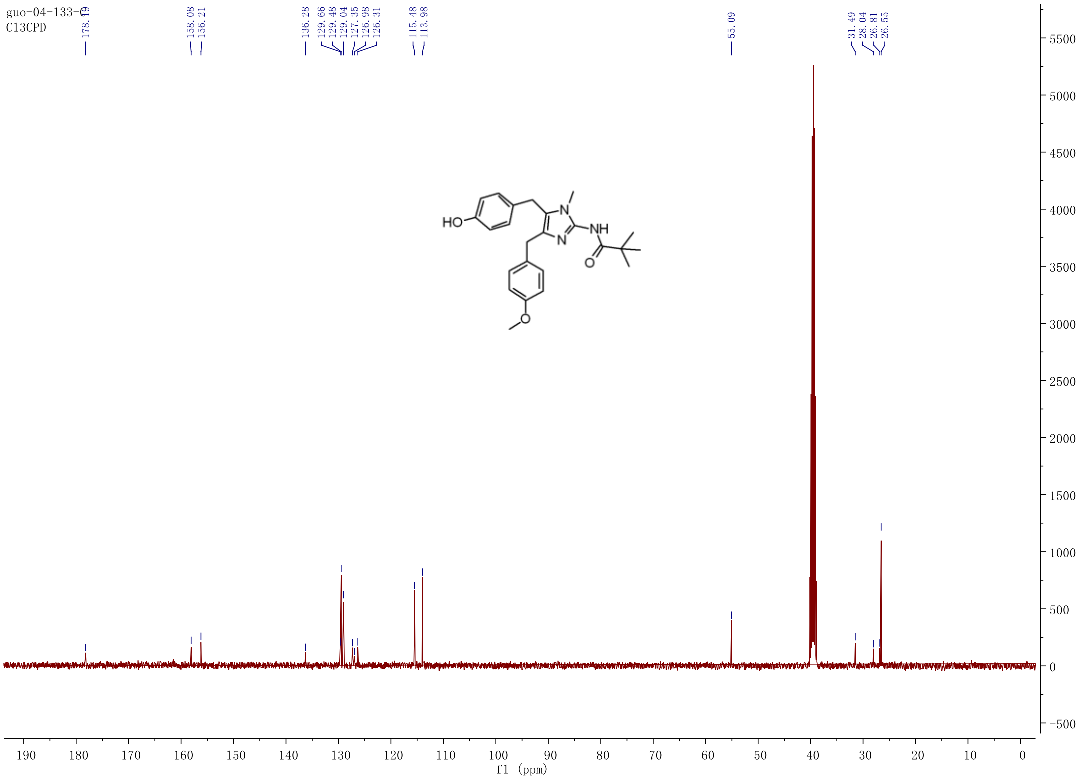

^{13}C NMR spectrum of **1e**

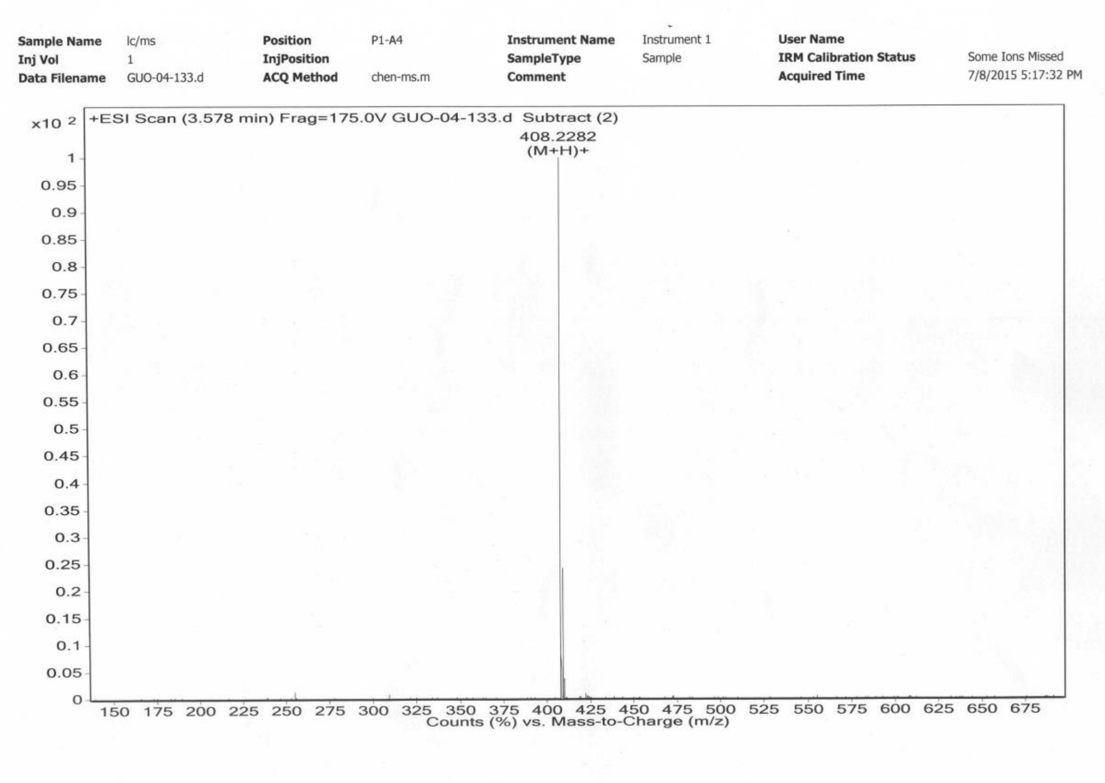

HRMS spectrum of **1e**

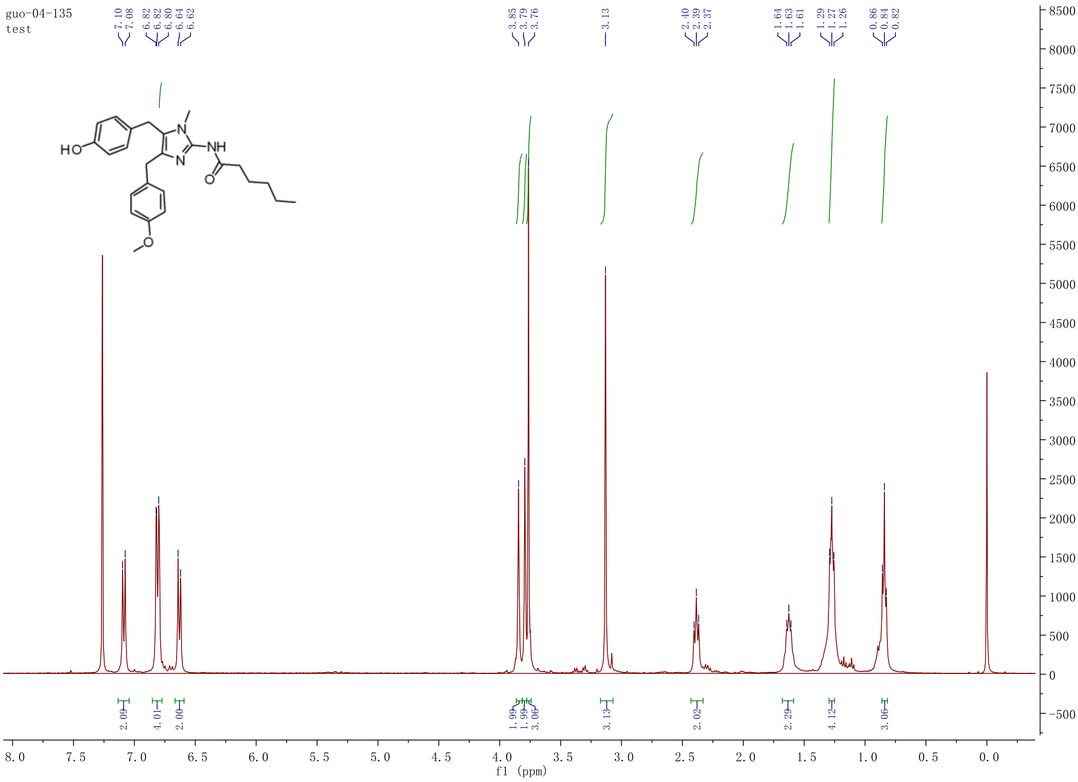

^{1}H NMR spectrum of **1f**

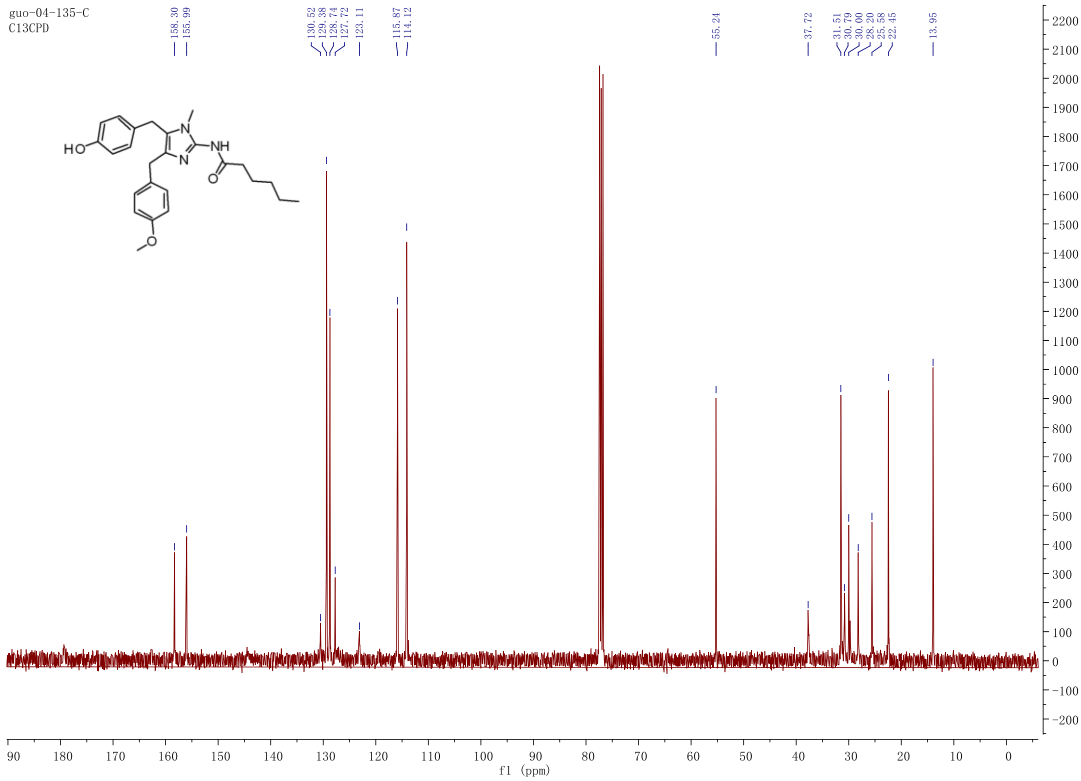

^{13}C NMR spectrum of **1f**

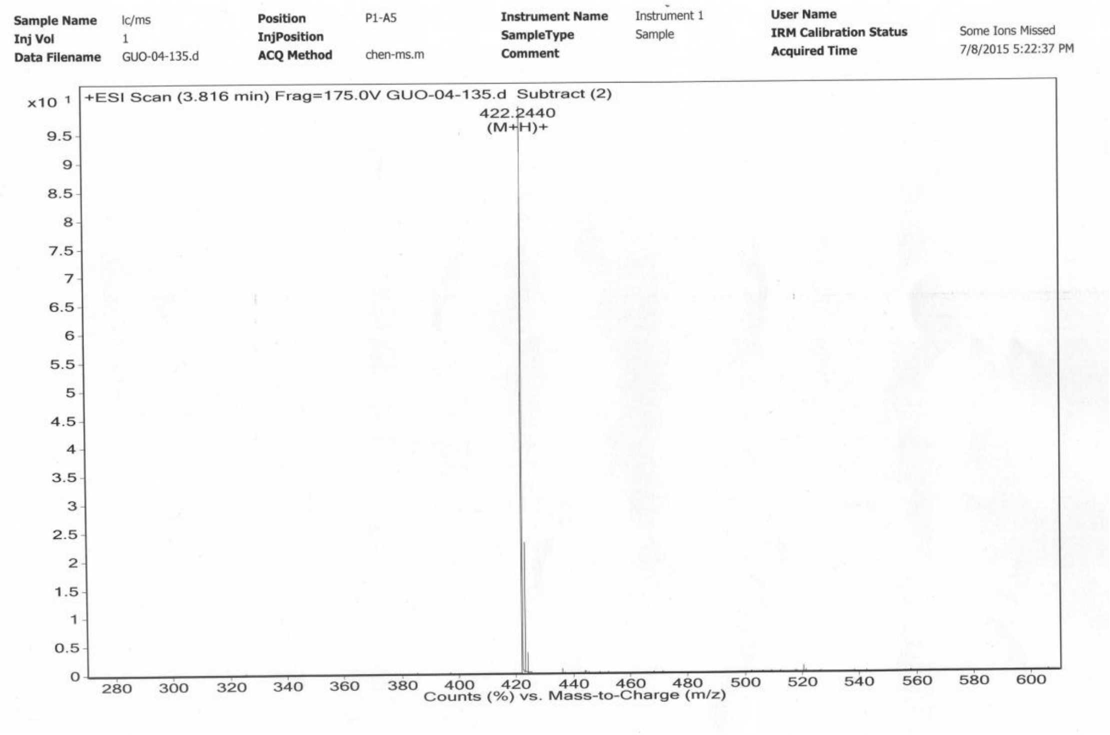

HRMS spectrum of **1f**

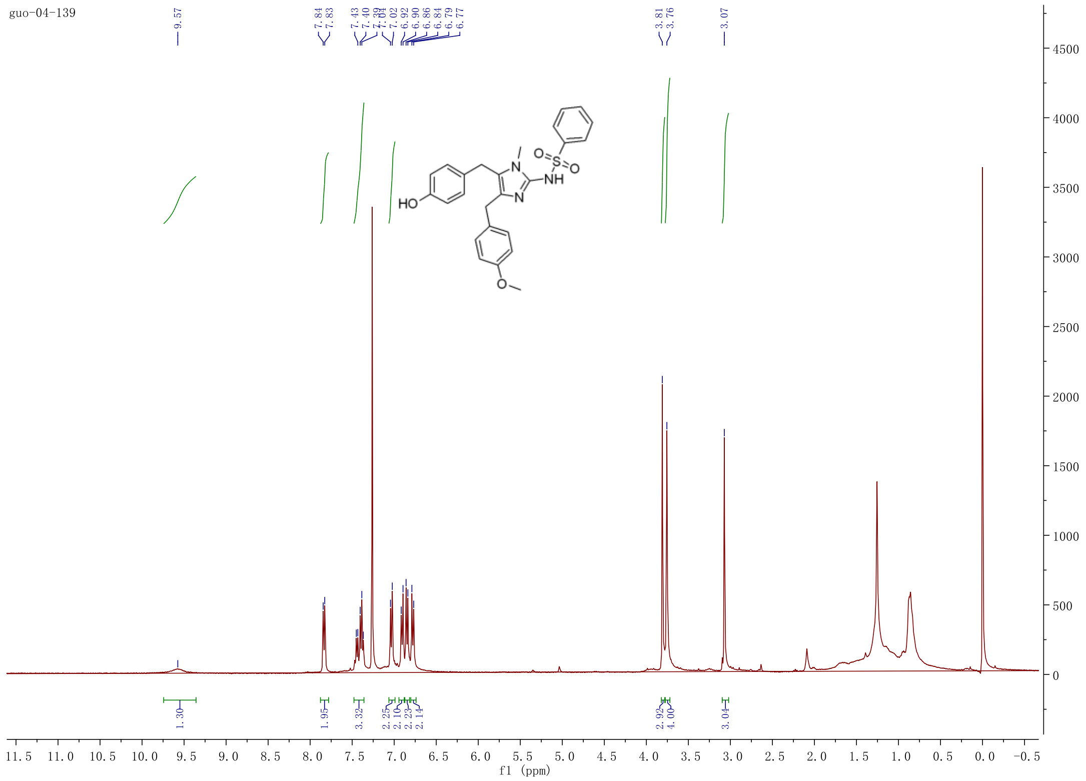

^{1}H NMR spectrum of **1g**

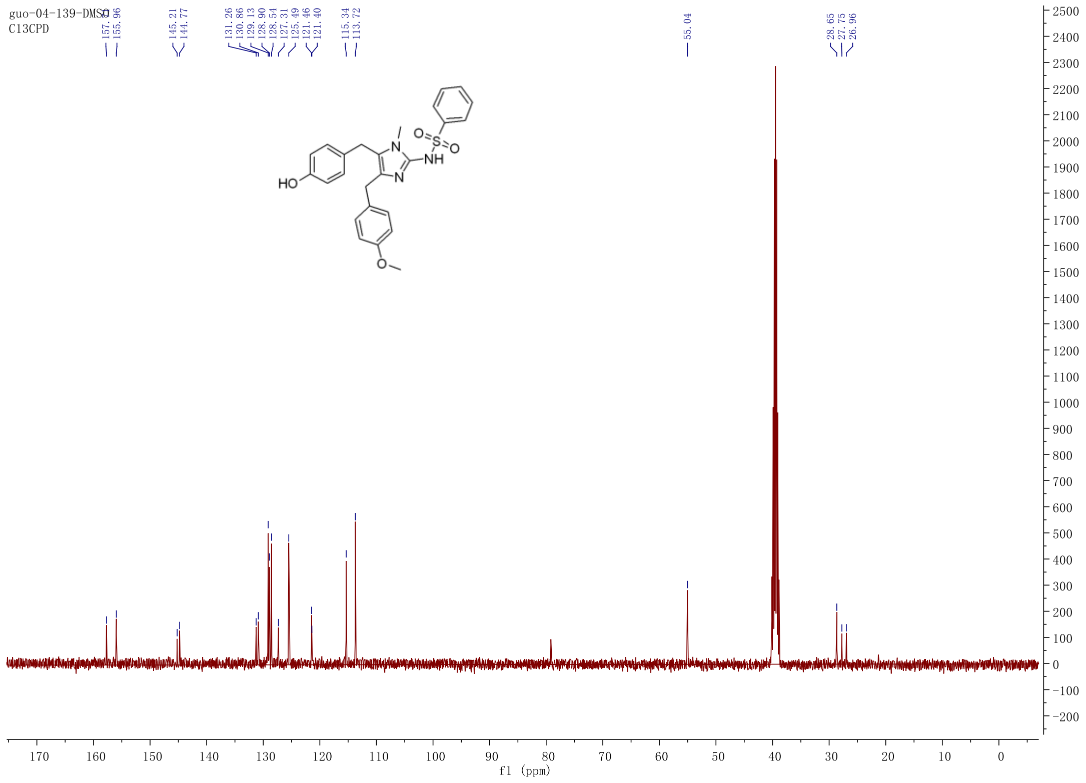

^{13}C NMR spectrum of **1g**

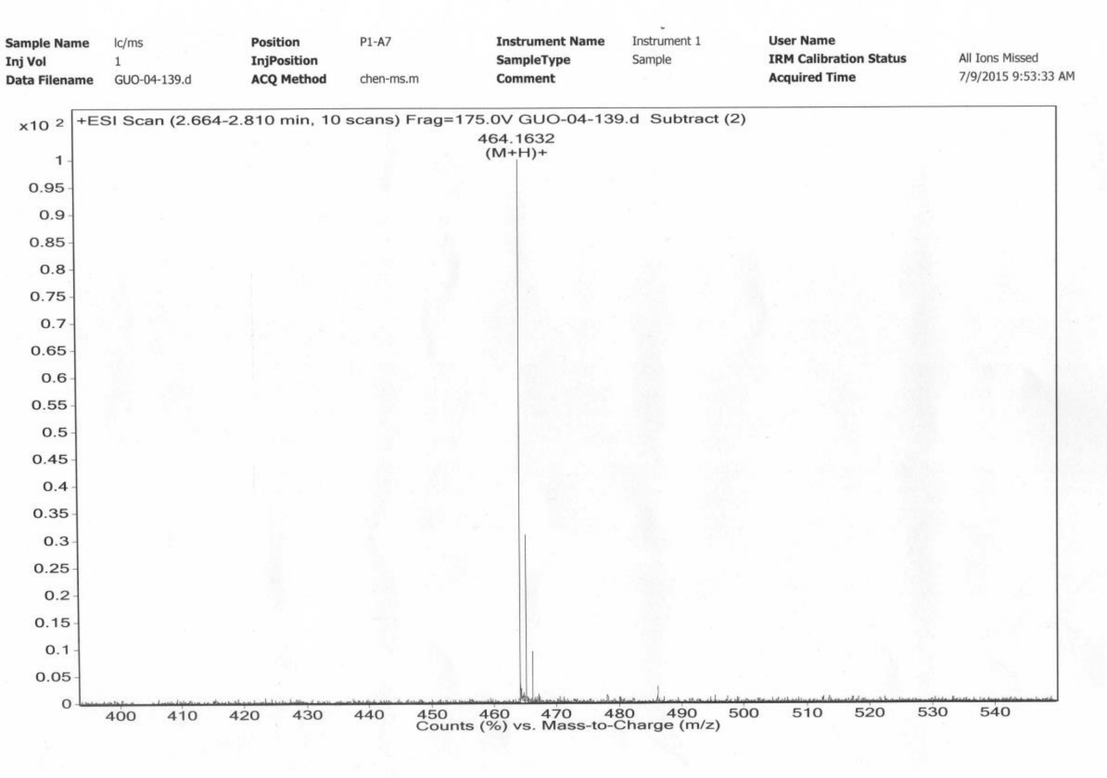

HRMS spectrum of **1g**

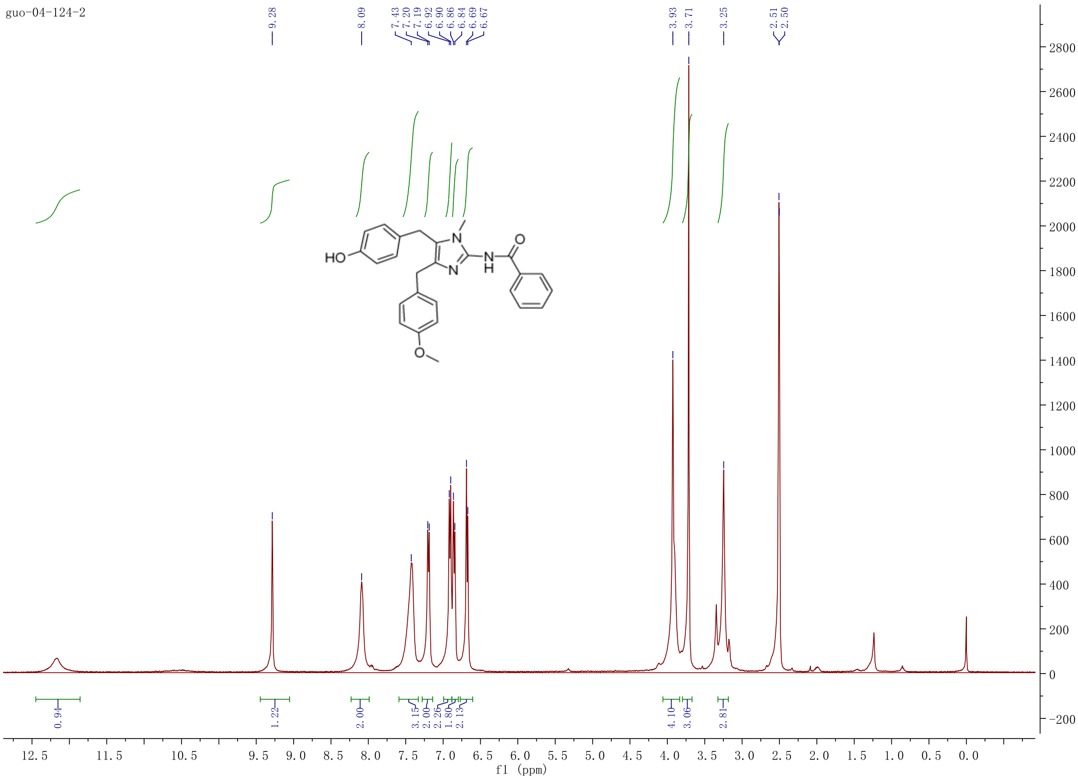

^{1}H NMR spectrum of **1h**

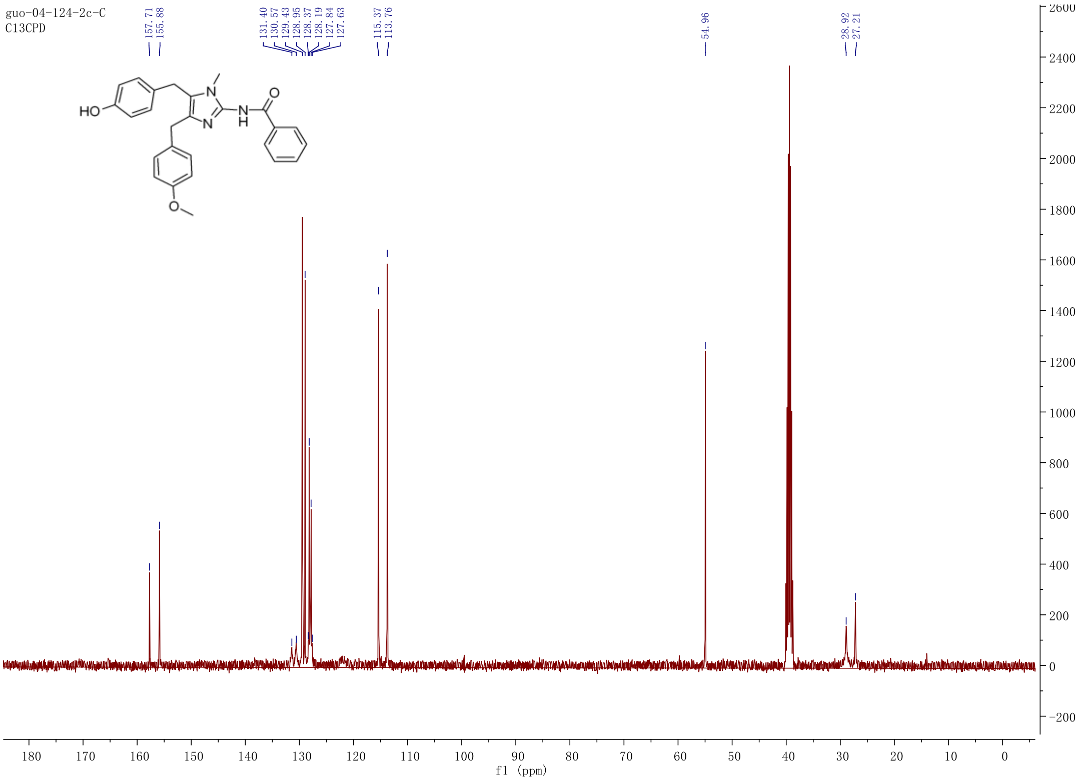

^{13}C NMR spectrum of **1h**

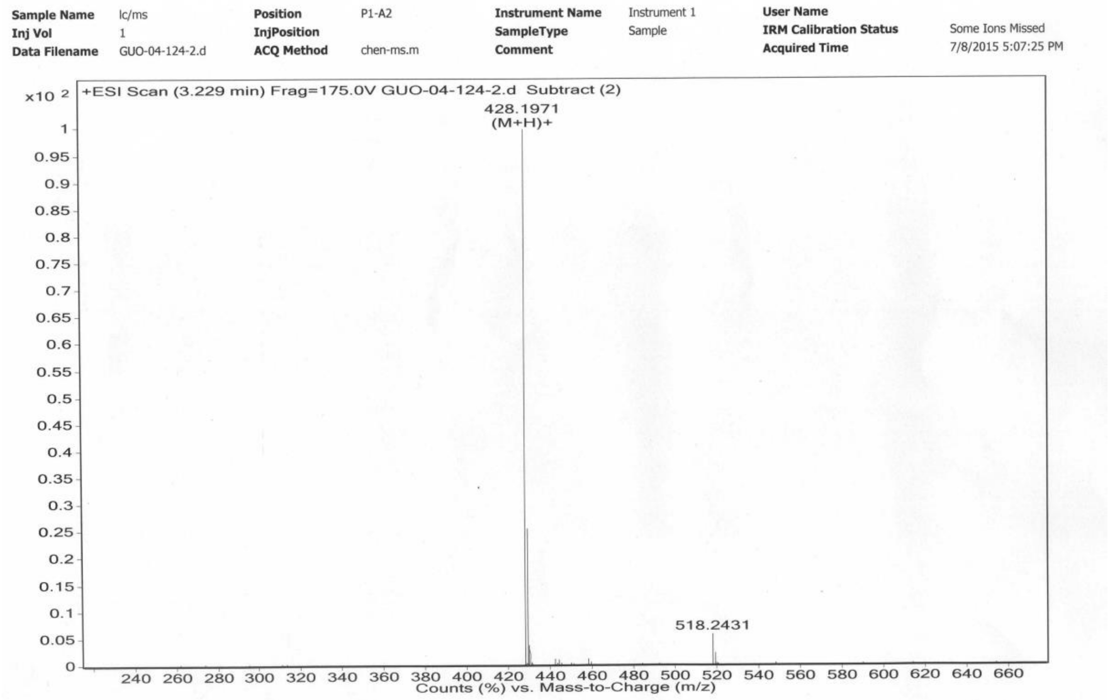

HRMS spectrum of **1h**

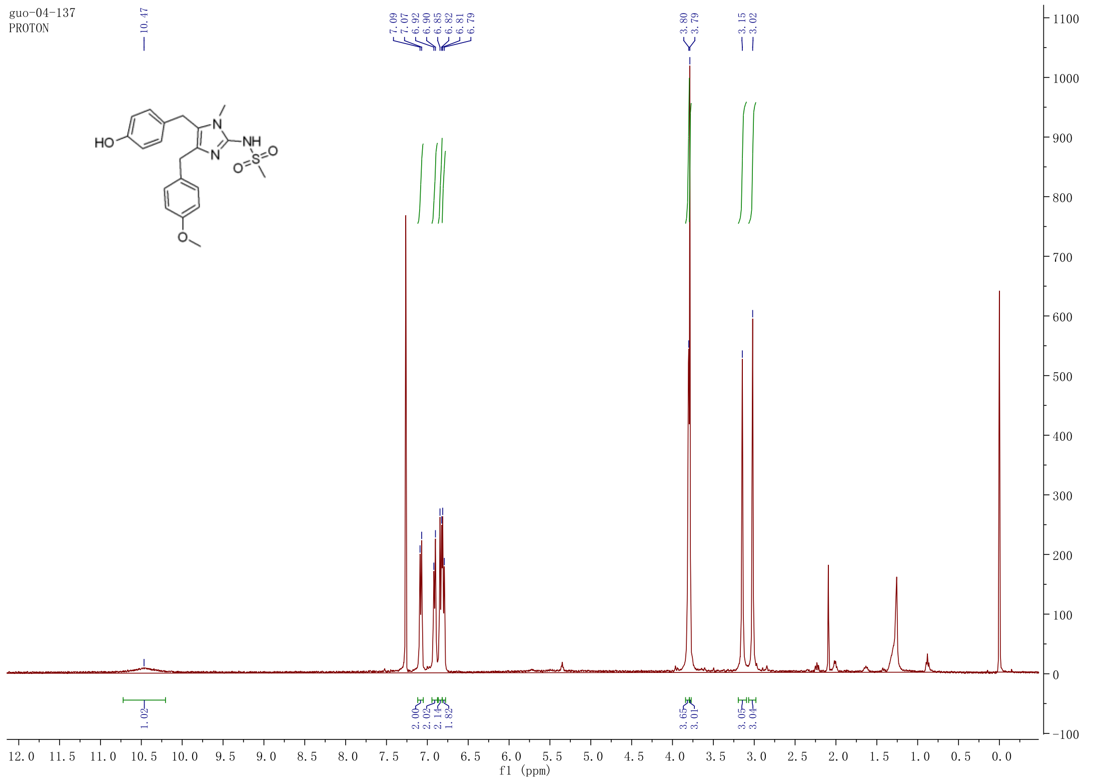

^{1}H NMR spectrum of **1i**

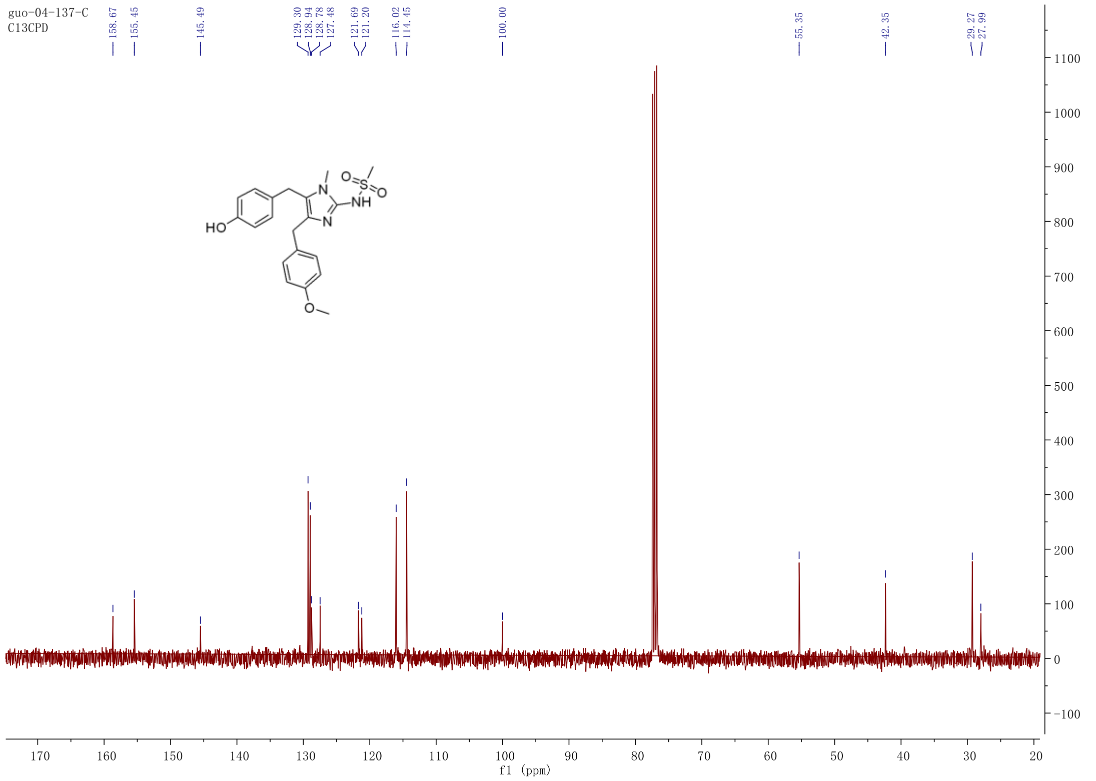

^{13}C NMR spectrum of **1i**

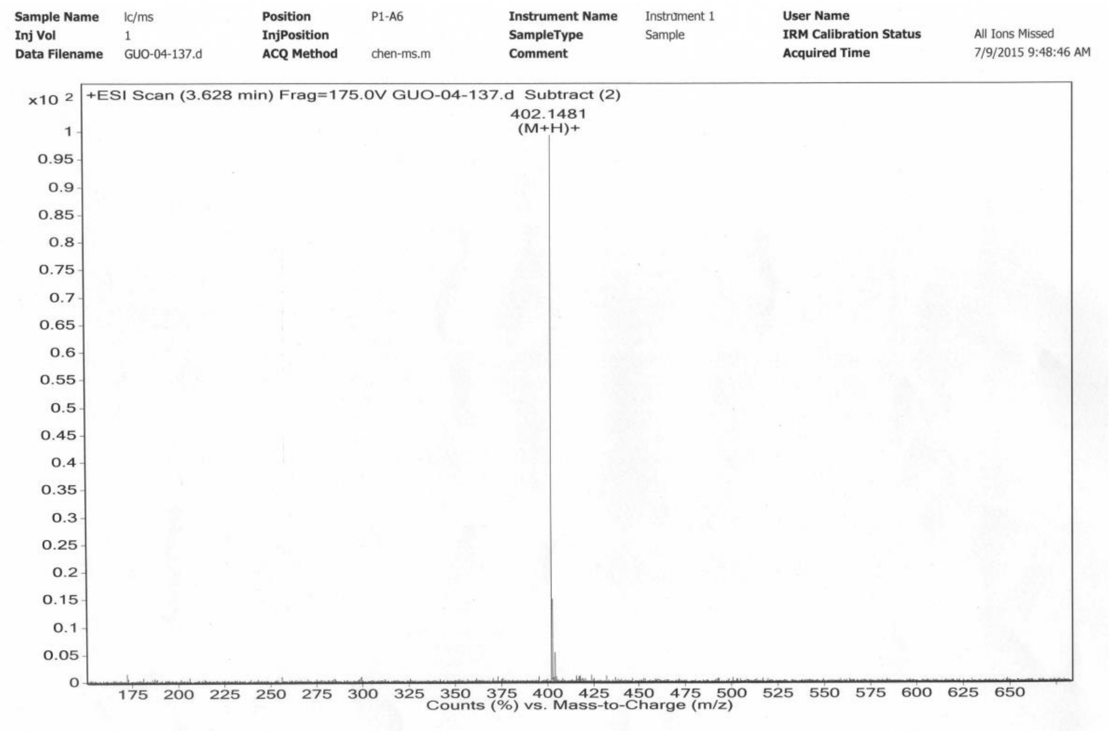

HRMS spectrum of **1i**

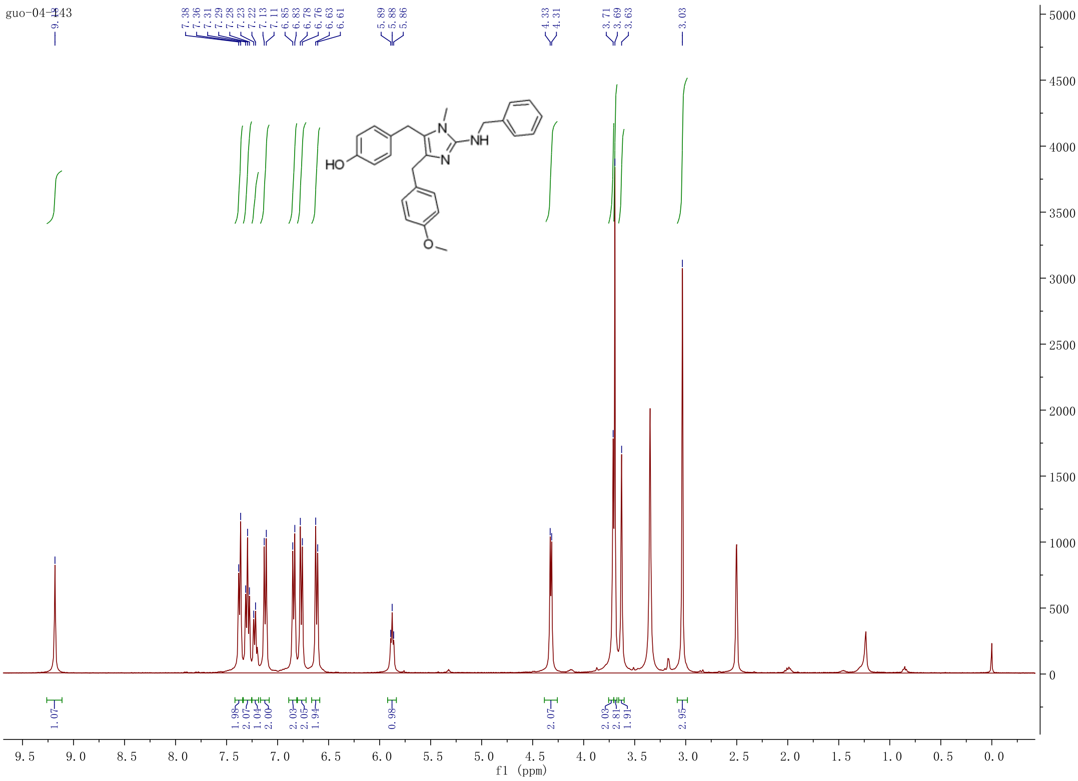

^{1}H NMR spectrum of **1j**

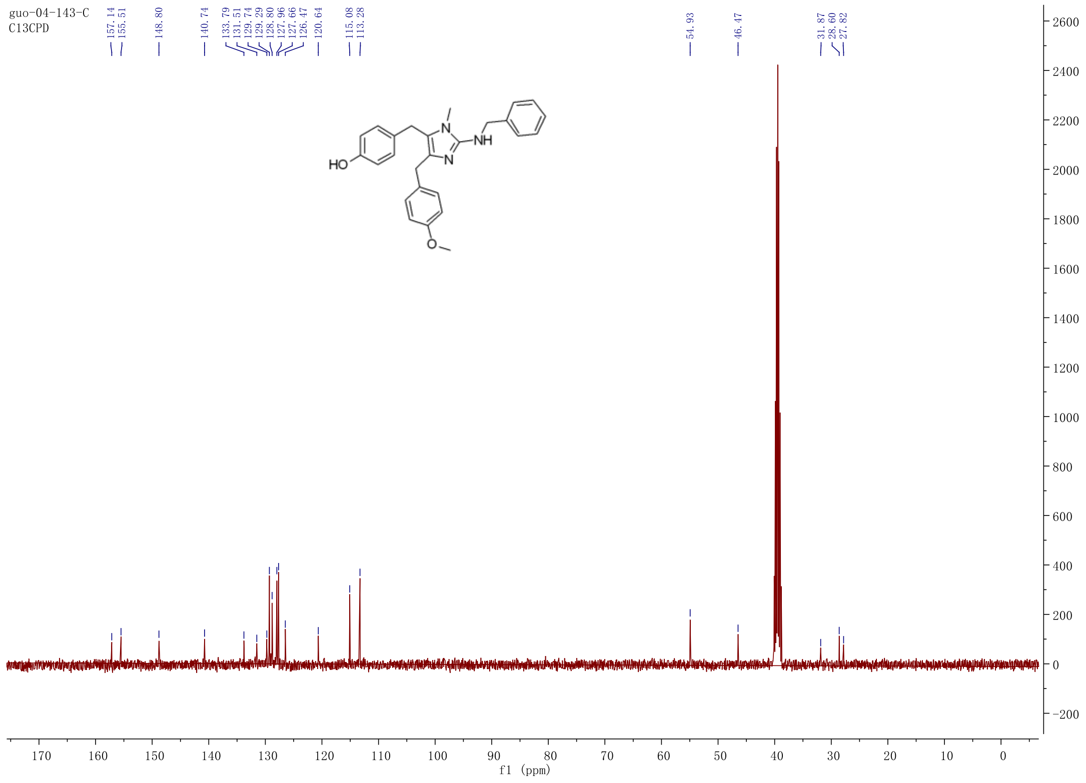

^{13}C NMR spectrum of **1j**

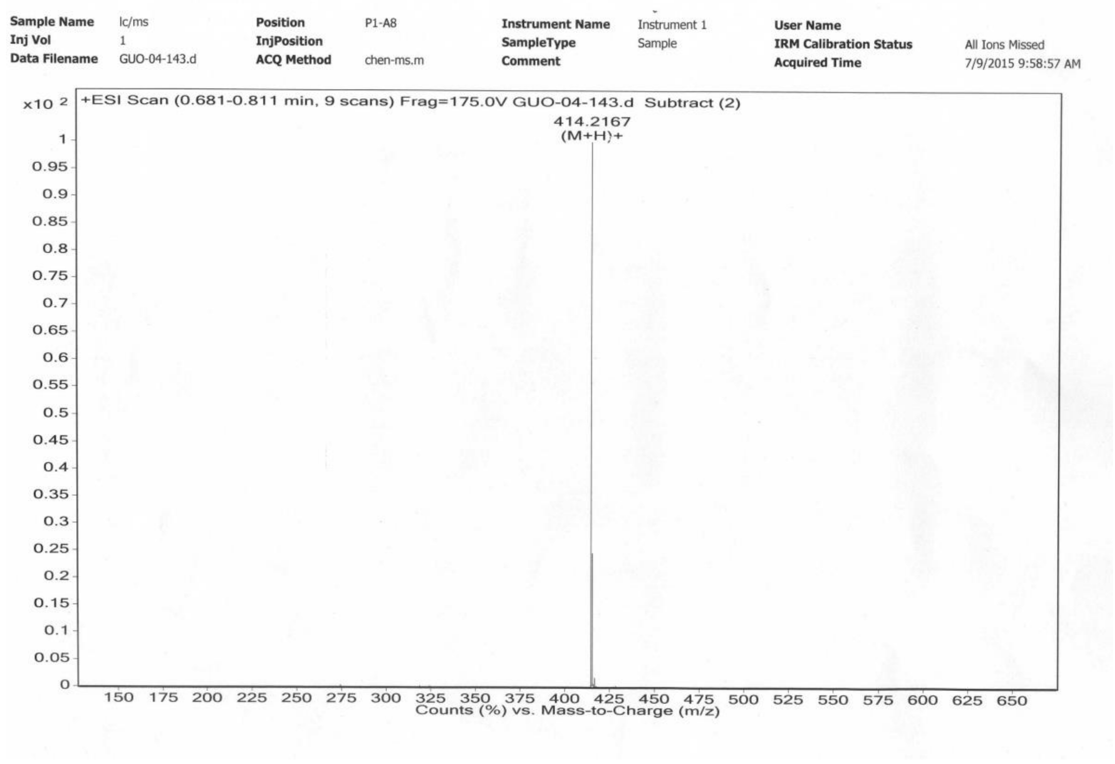

HRMS spectrum of **1j**

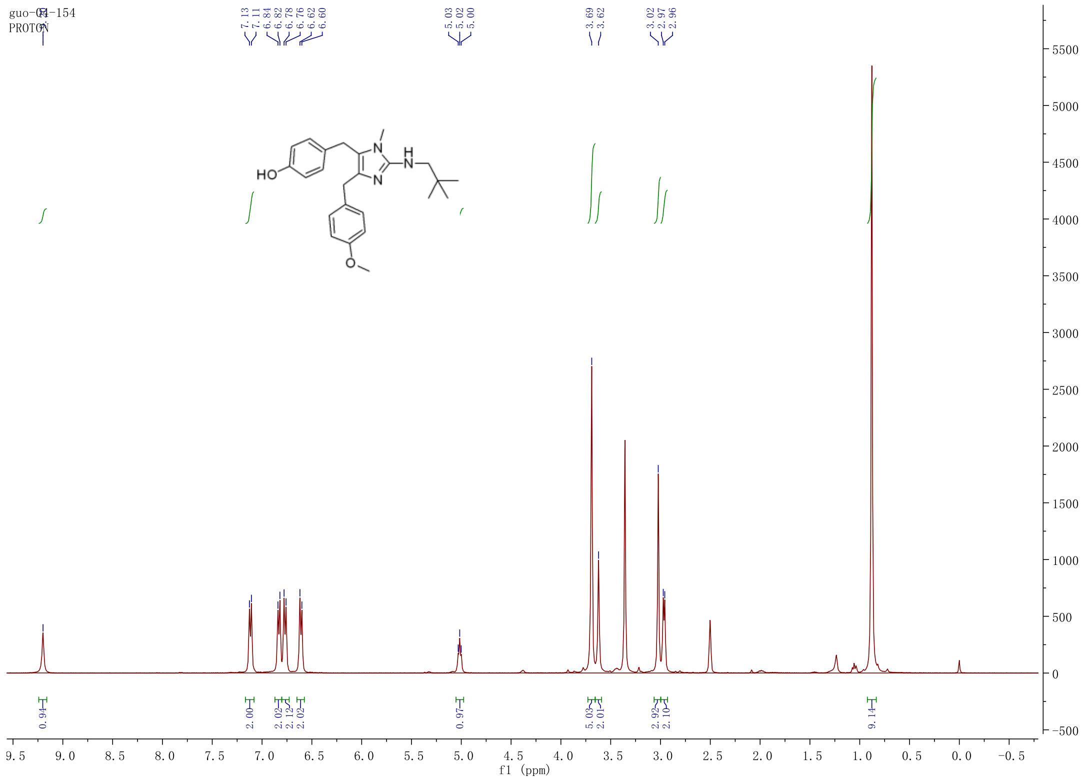

^{1}H NMR spectrum of **1k**

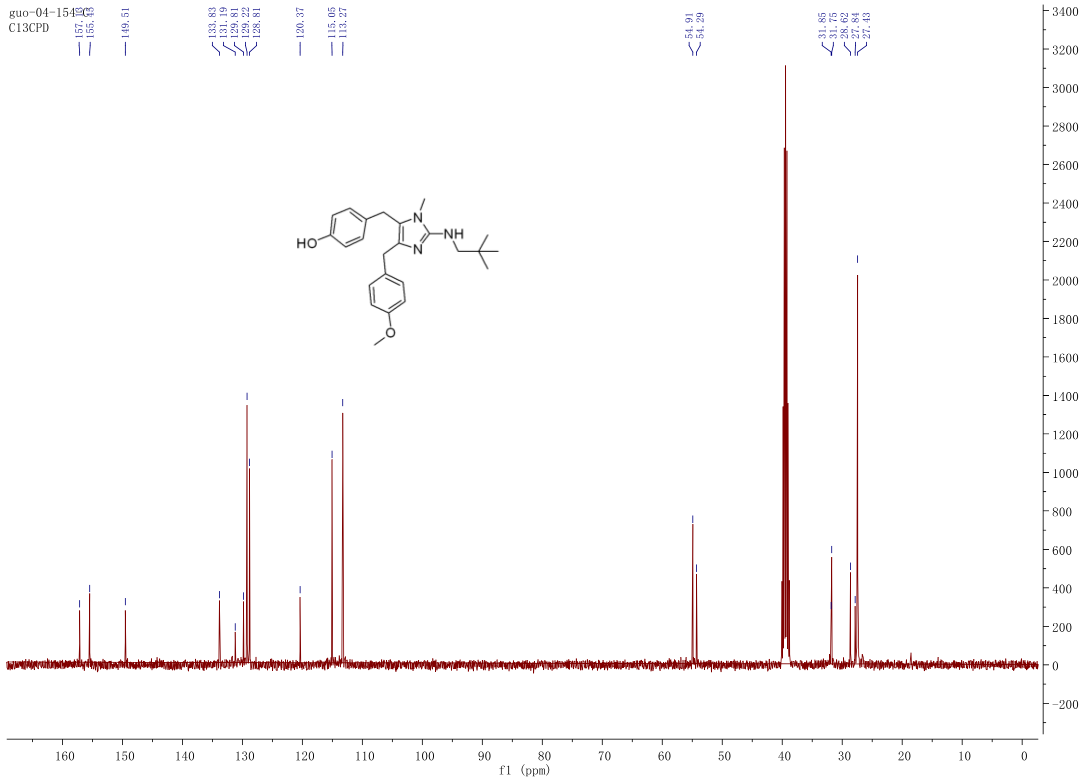

^{13}C NMR spectrum of **1k**

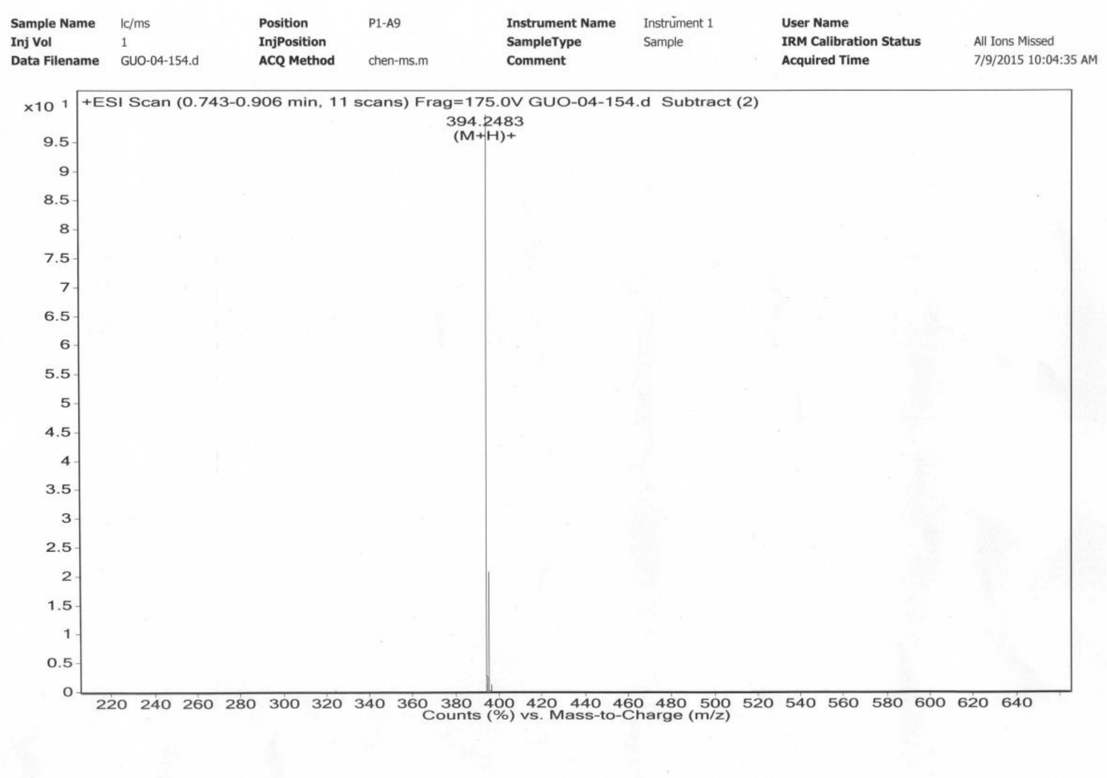

HRMS spectrum of **1k**

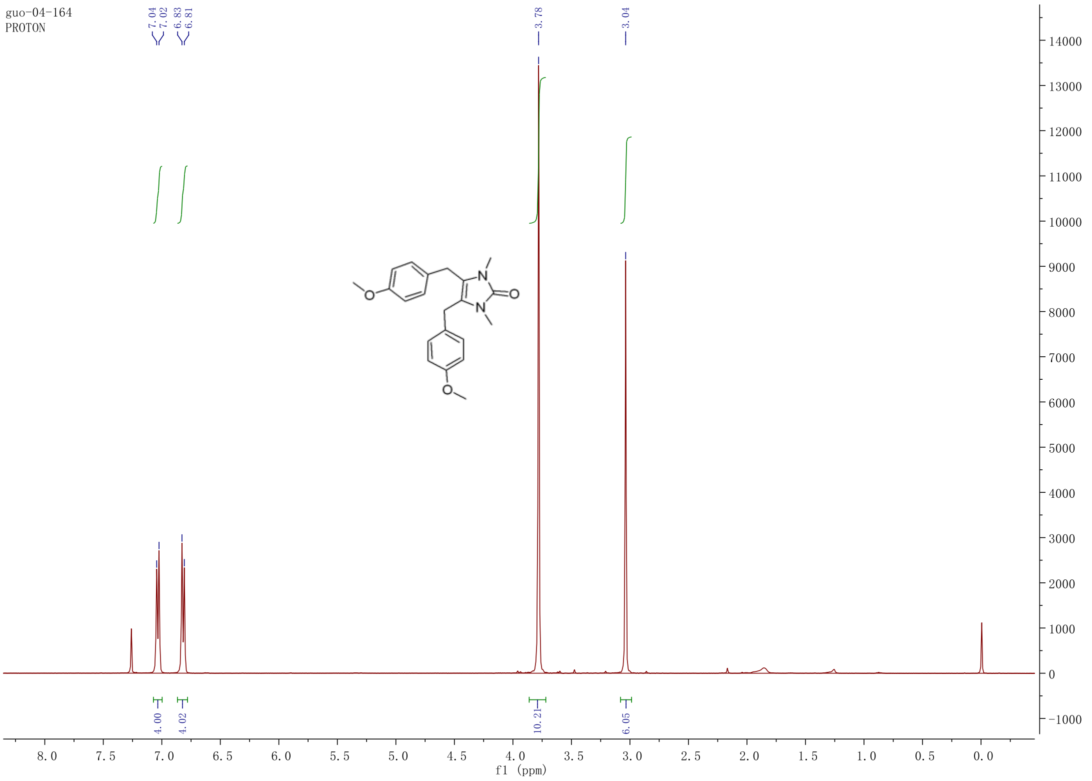

^{1}H NMR spectrum of **1l**

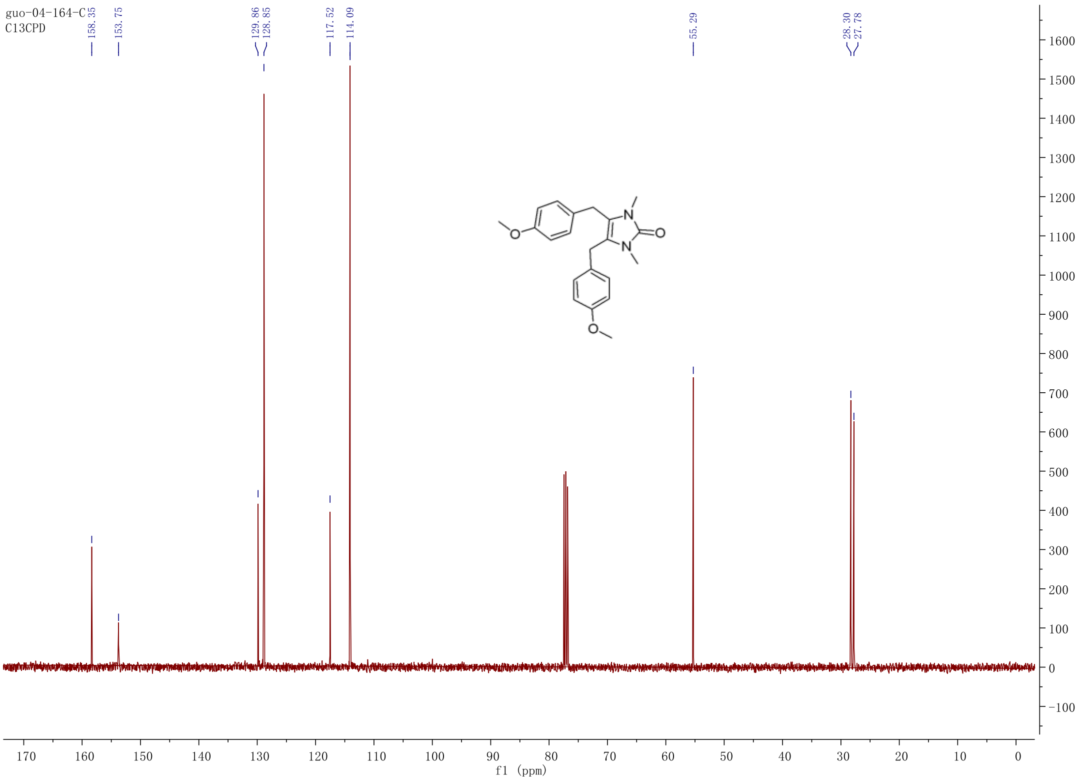

^{13}C NMR spectrum of **1l**

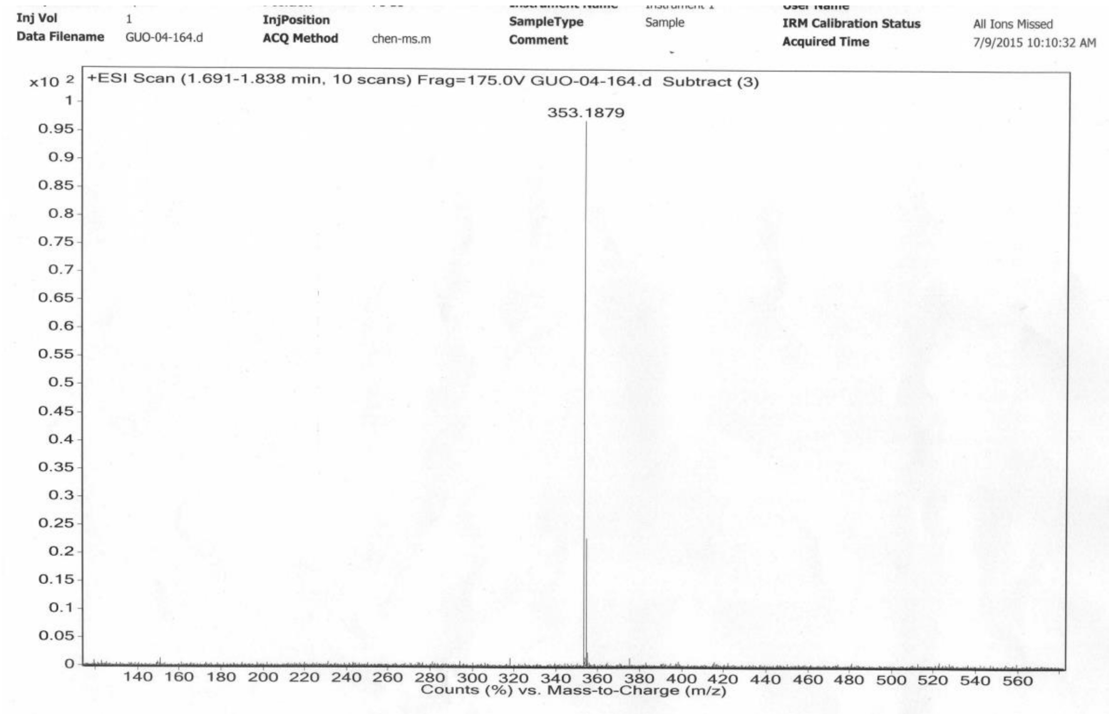

HRMS spectrum of **1l**

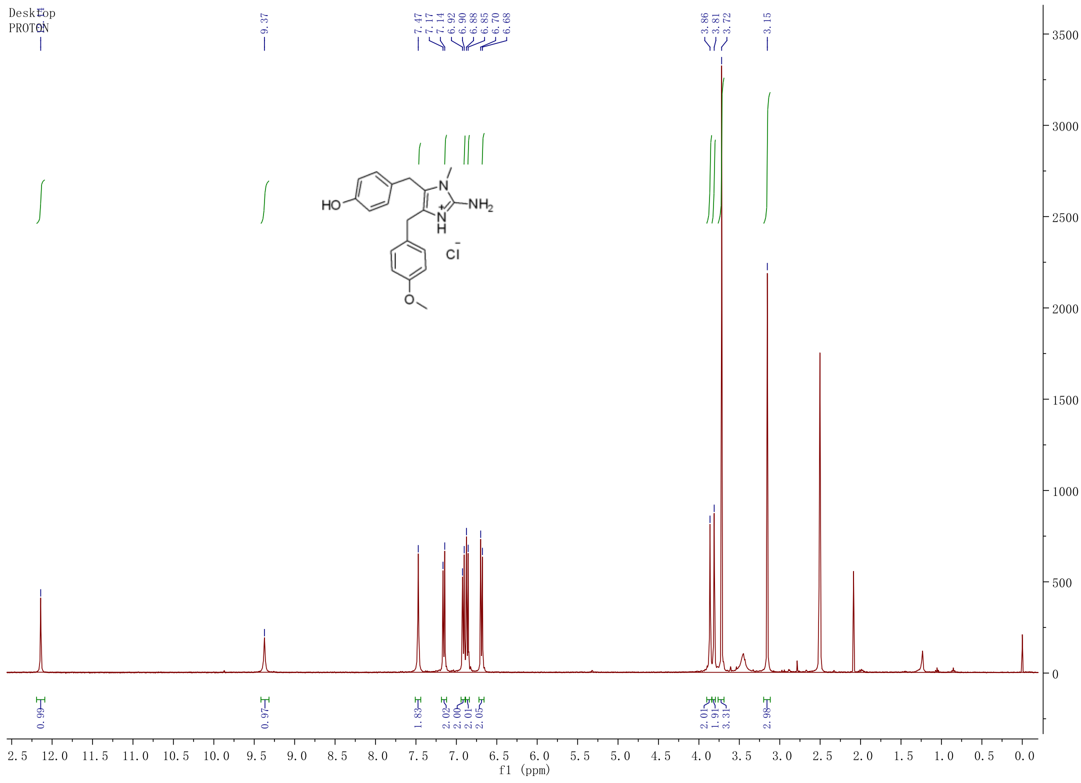

^{1}H NMR spectrum of **1m**

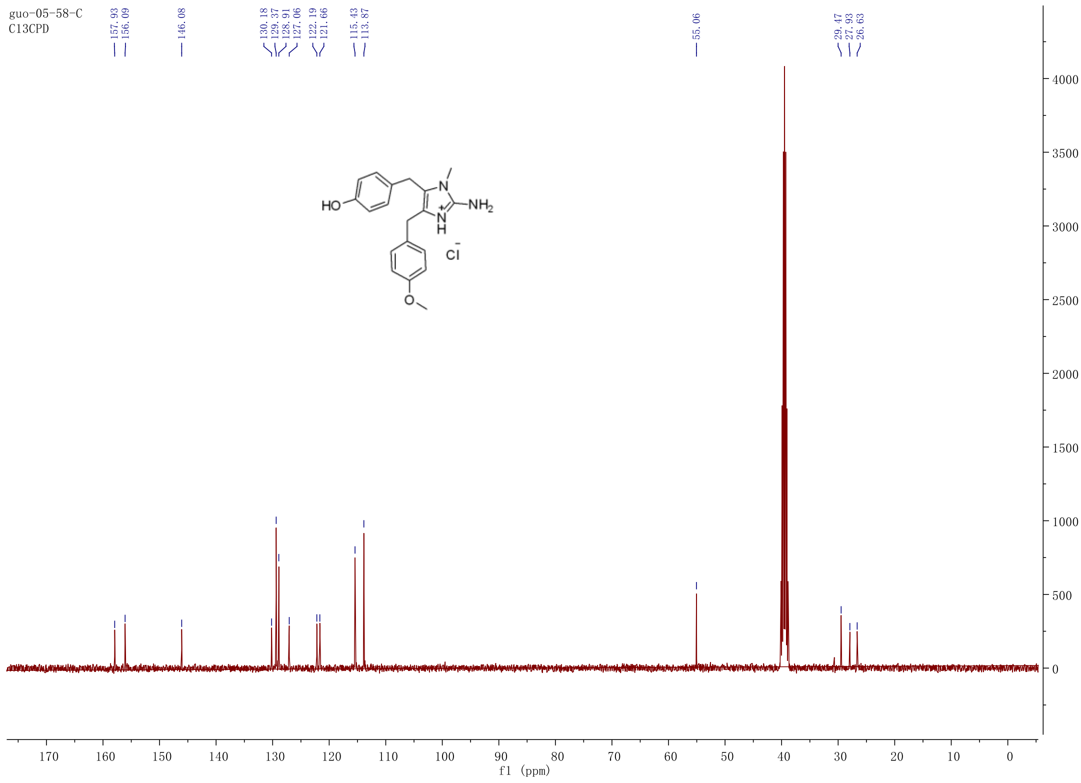

^{13}C NMR spectrum of **1m**

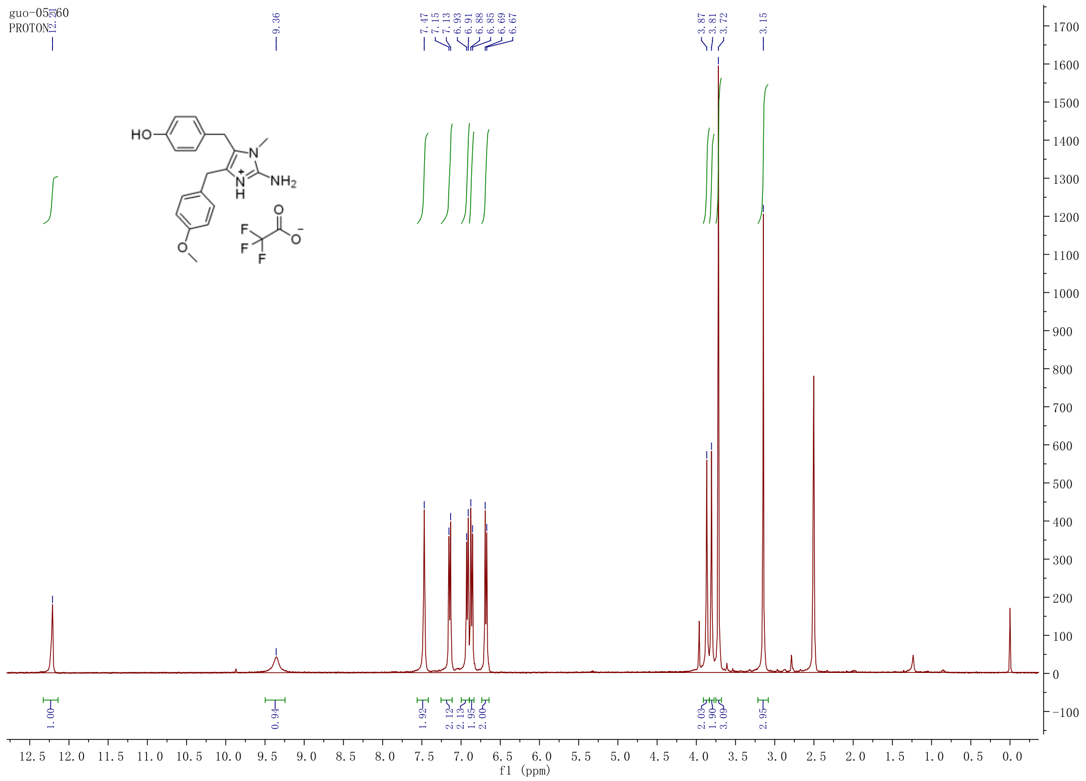

^{1}H NMR spectrum of **1n**

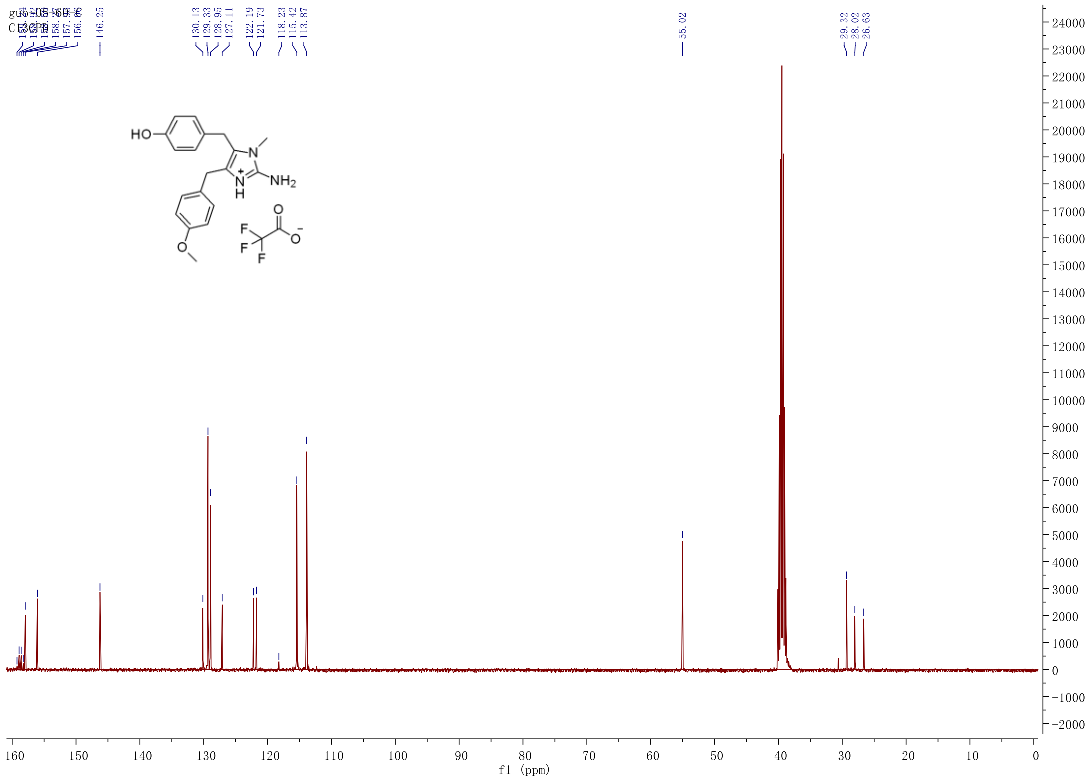

^{13}C NMR spectrum of **1n**

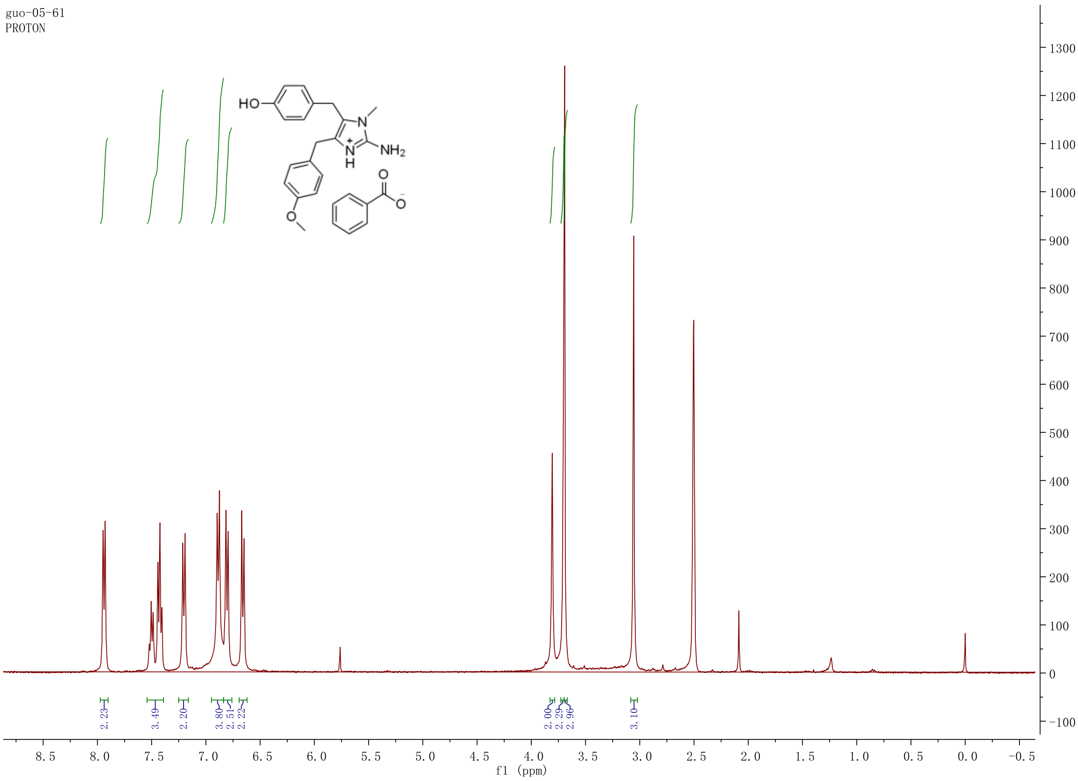

^{1}H NMR spectrum of **1o**

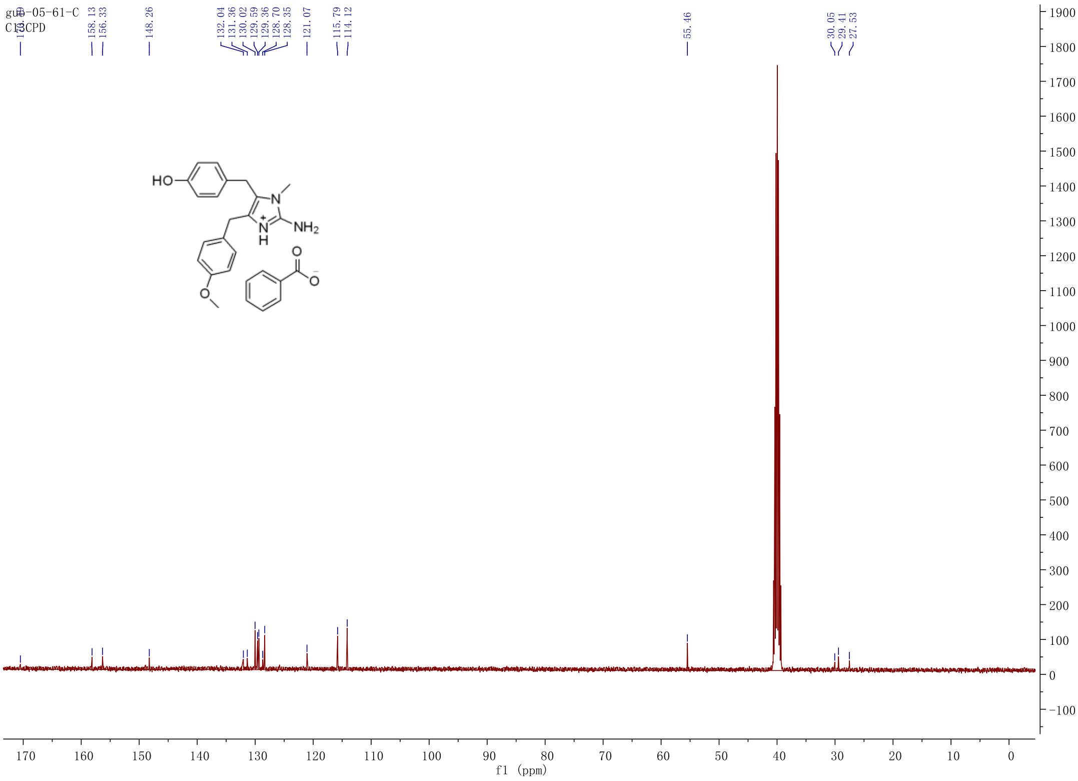

^{13}C NMR spectrum of **1o**

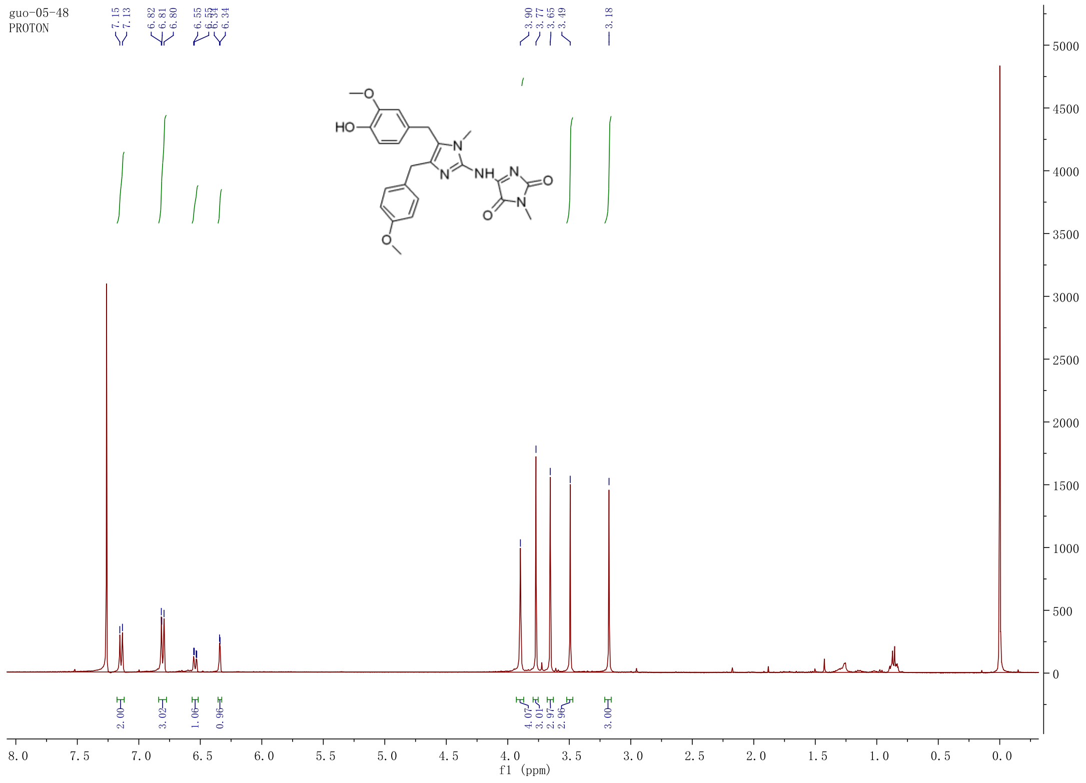

^{1}H NMR spectrum of **2a**

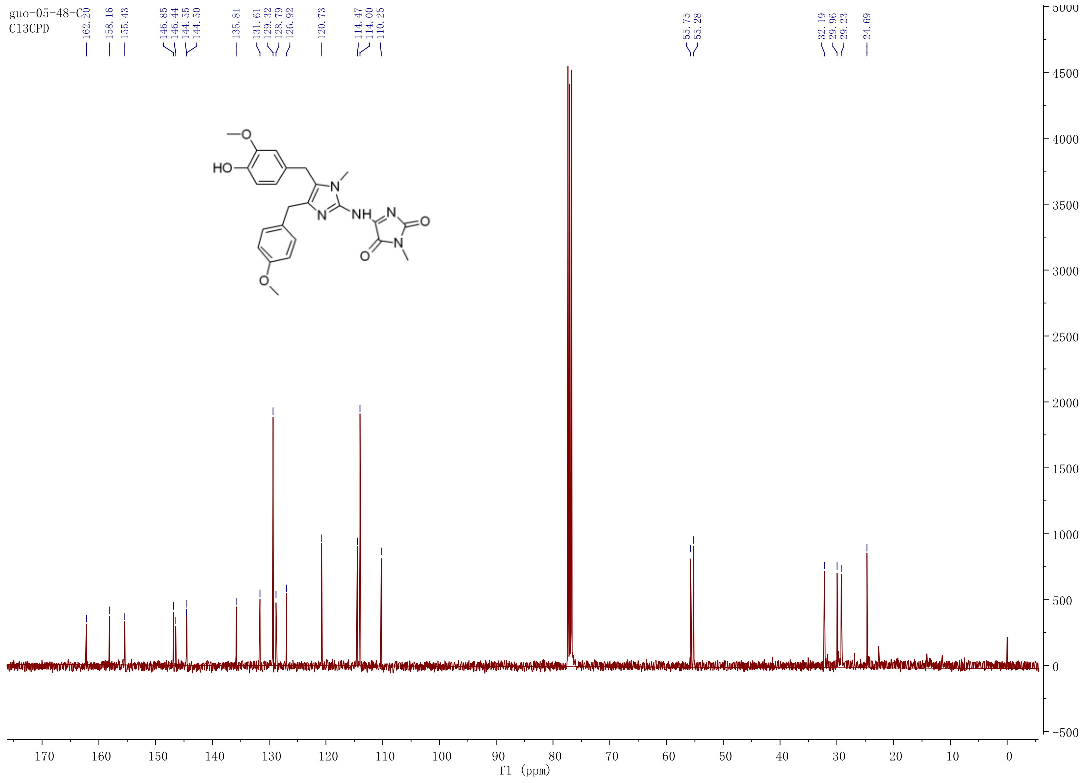

^{13}C NMR spectrum of **2a**

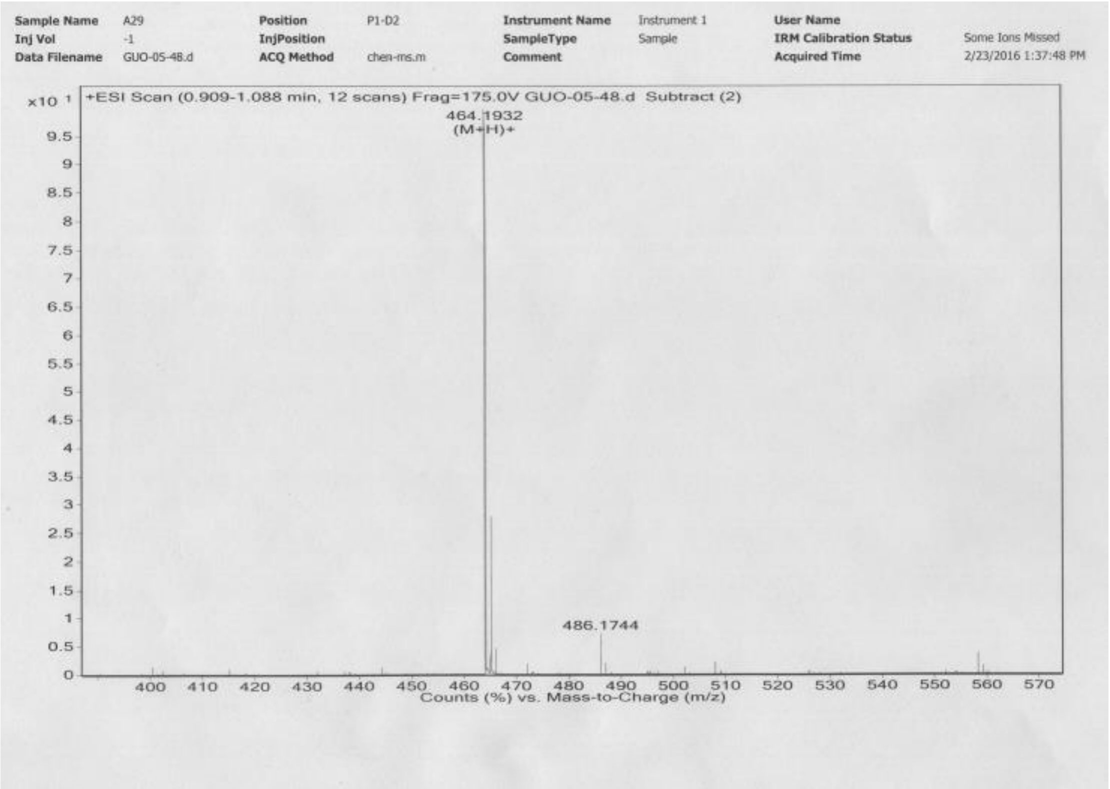

HRMS spectrum of **2a**

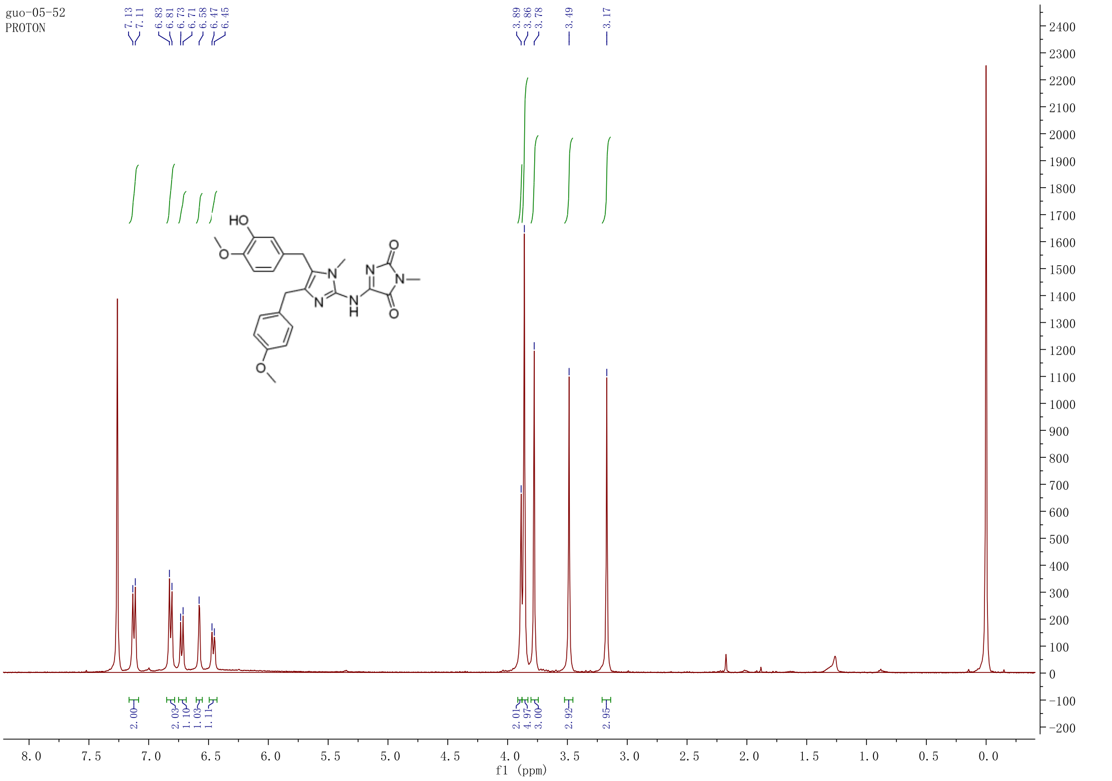

^{1}H NMR spectrum of **2b**

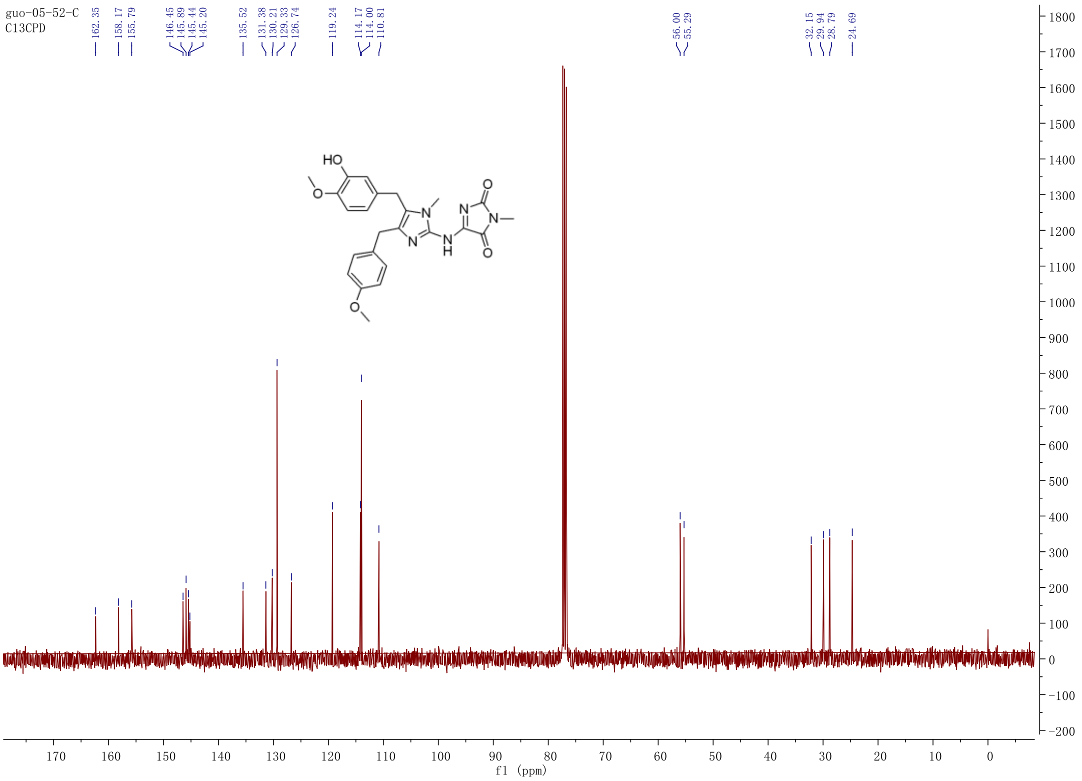

^{13}C NMR spectrum of **2b**

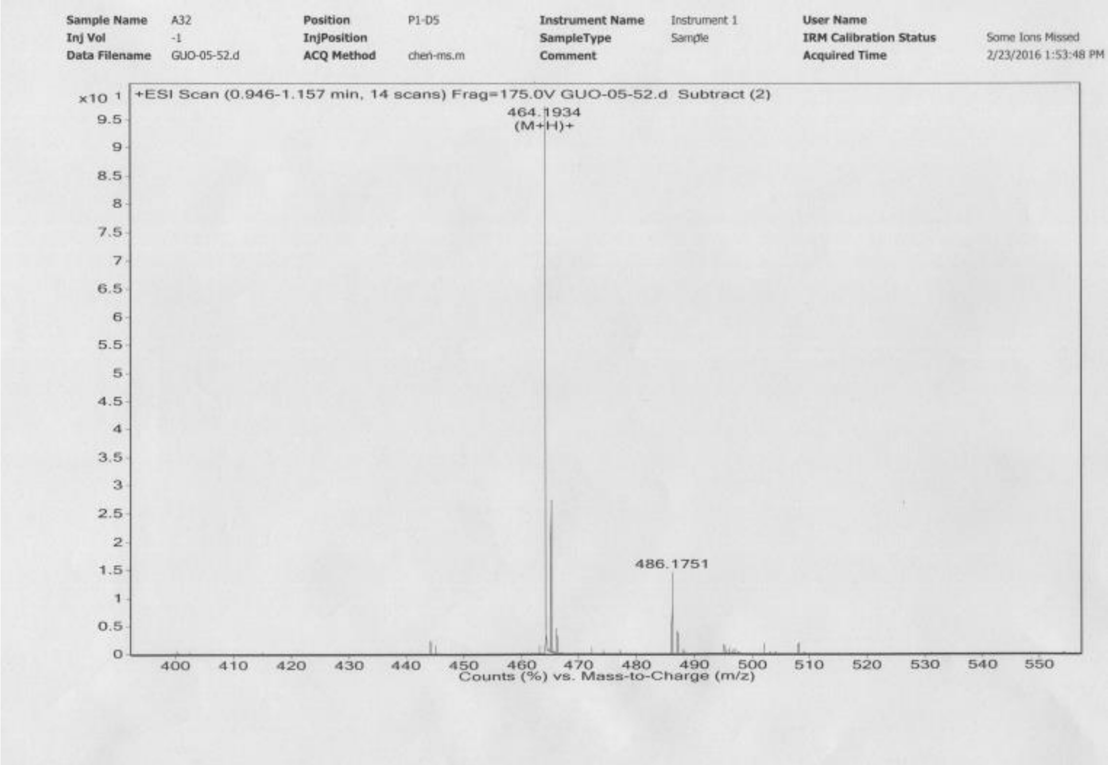

HRMS spectrum of **2b**

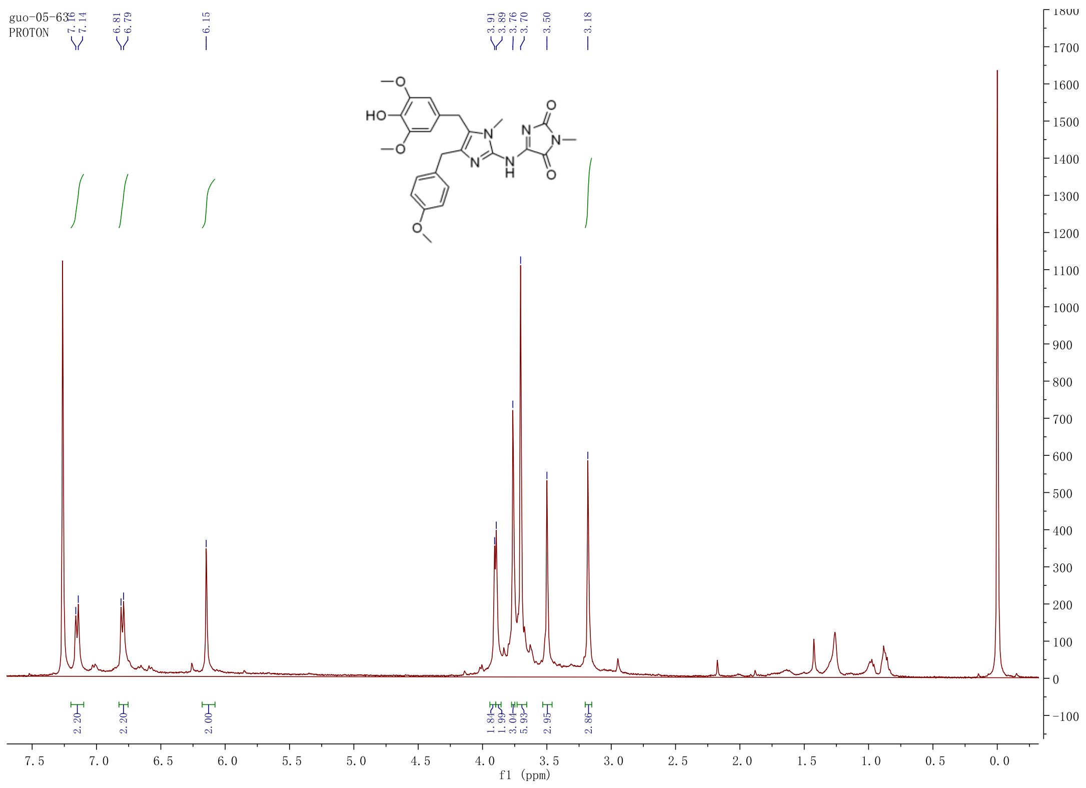

^{1}H NMR spectrum of **2c**

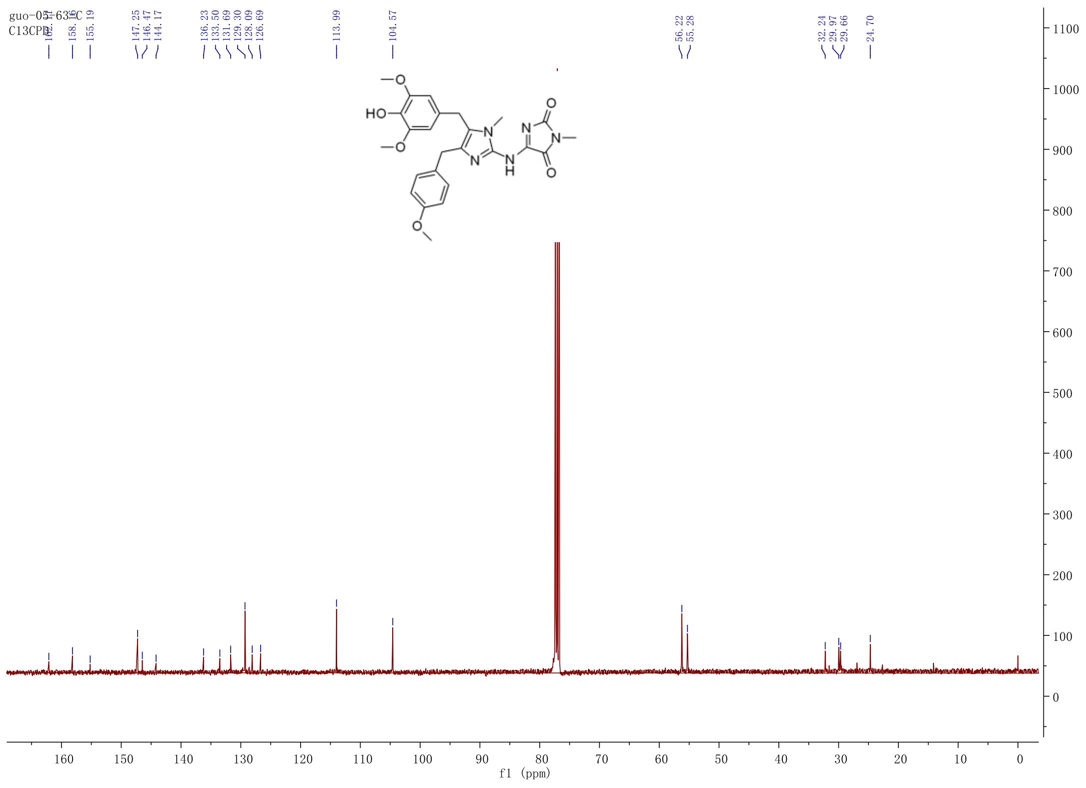

^{13}C NMR spectrum of **2c**

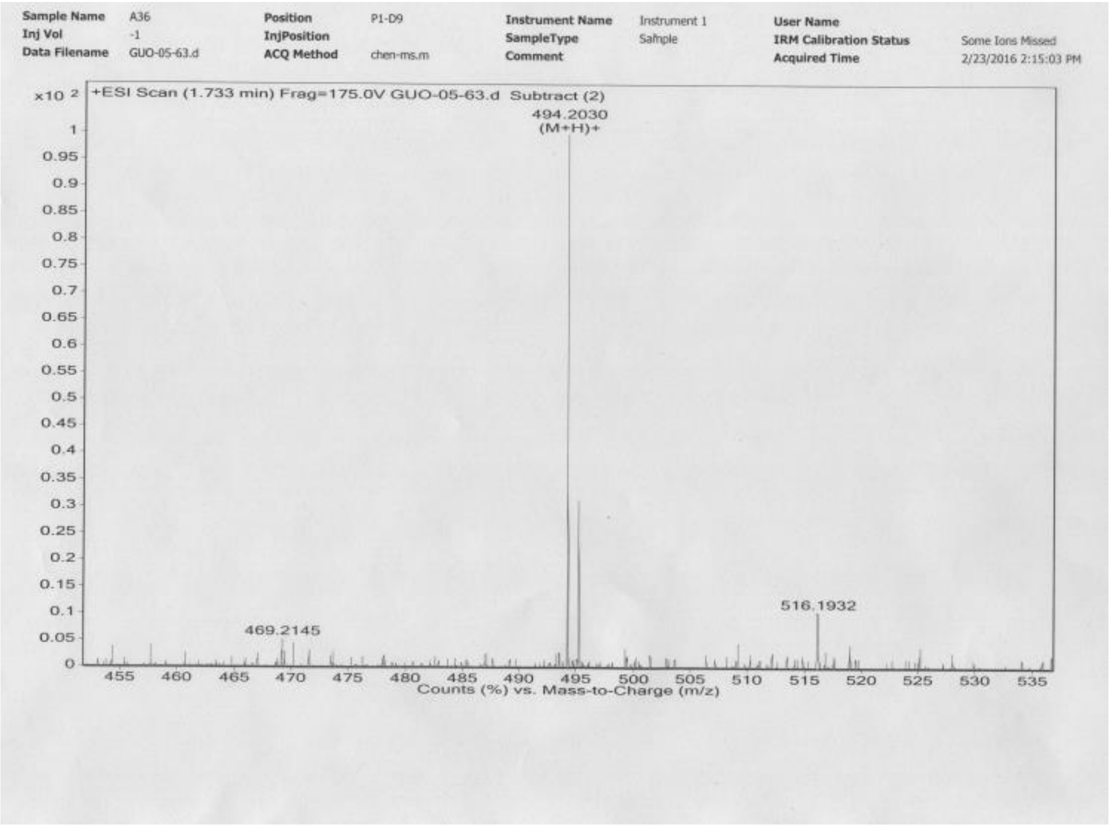

HRMS spectrum of **2c**

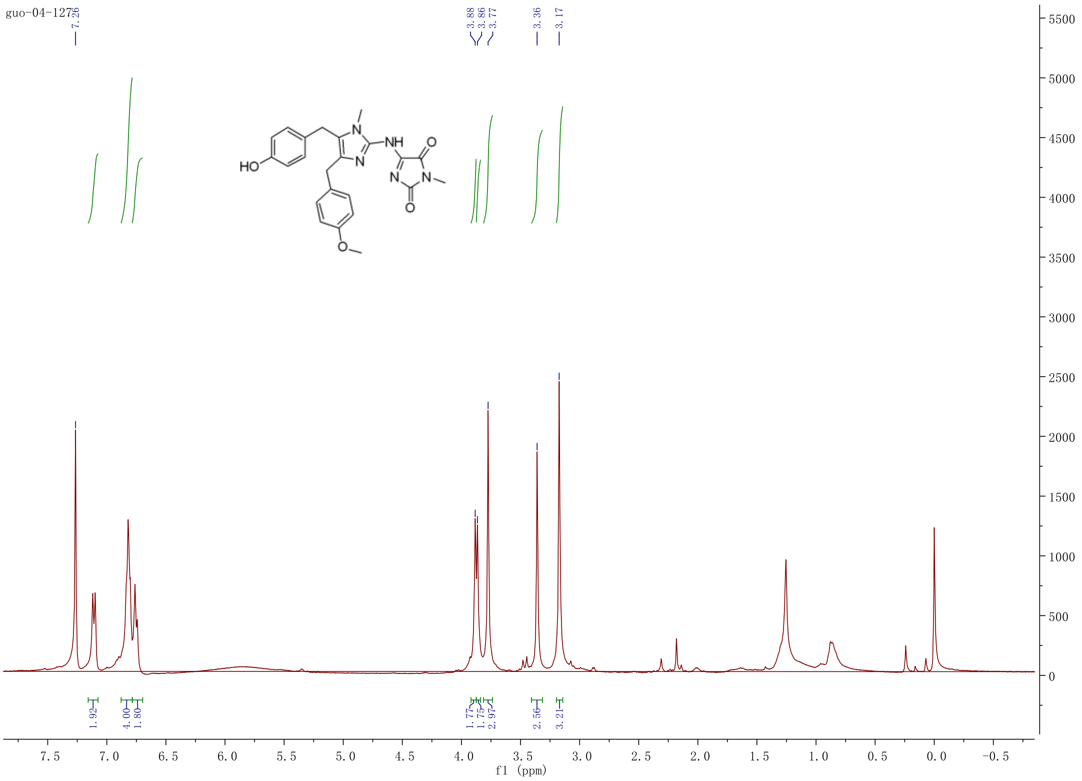

^{1}H NMR spectrum of **2d**

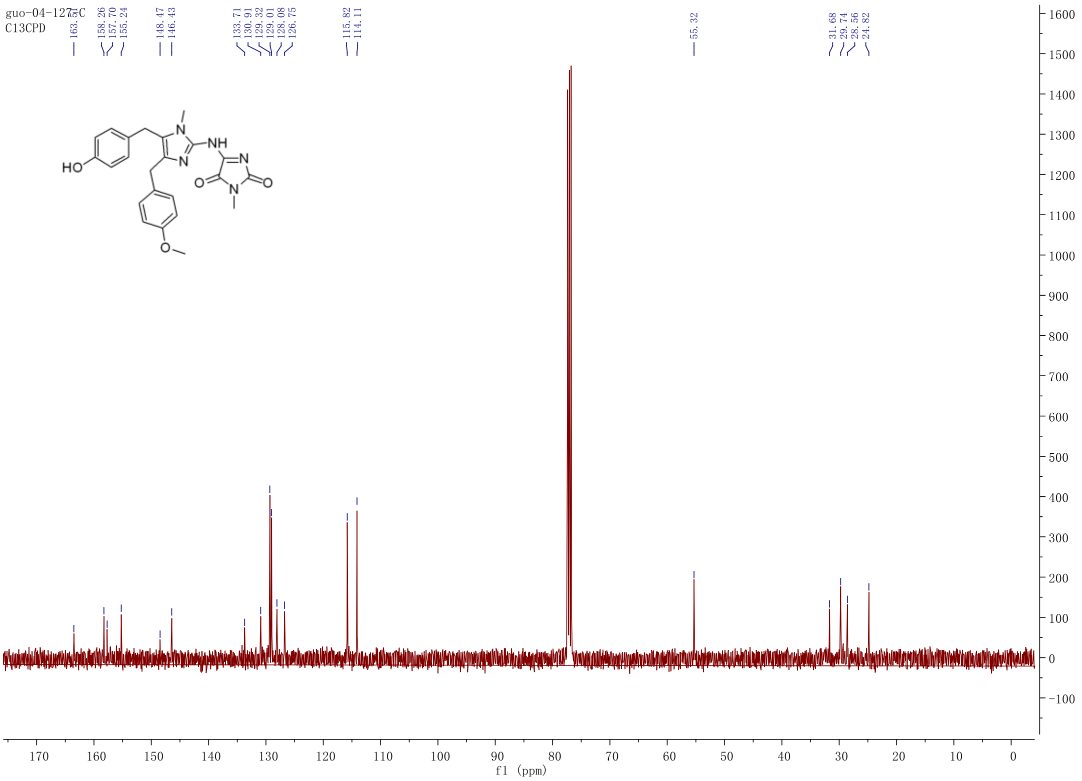

^{13}C NMR spectrum of **2d**

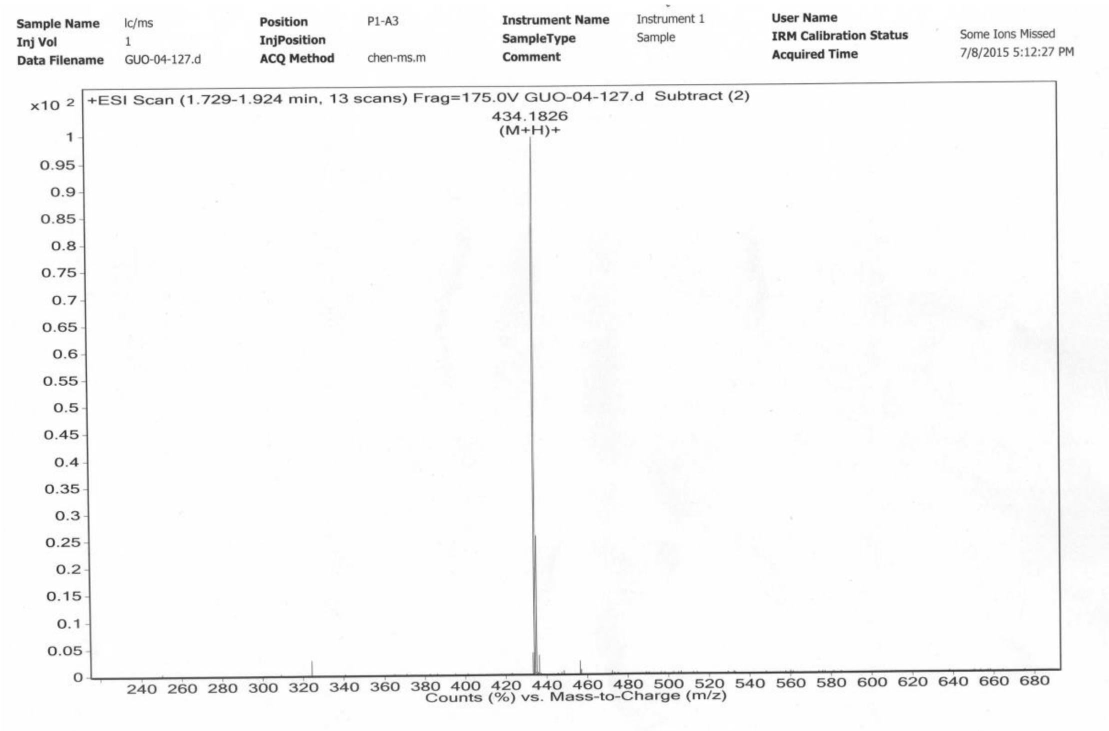

HRMS spectrum of **2d**

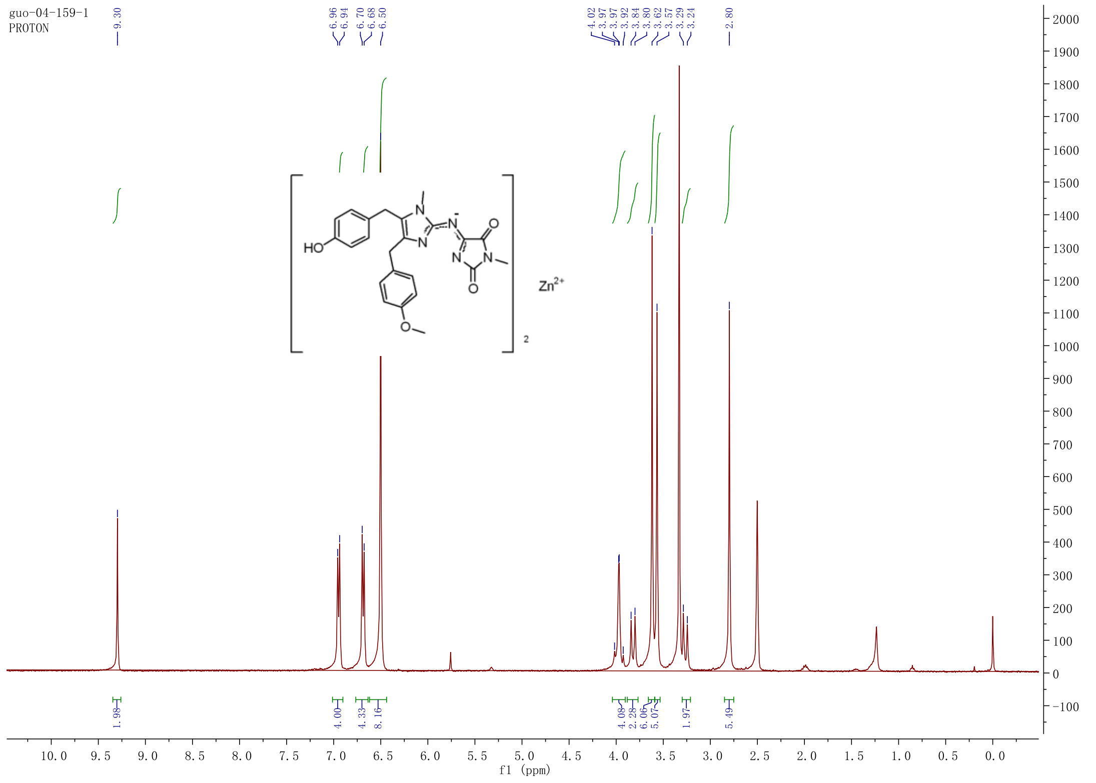

^{1}H NMR spectrum of **2e**

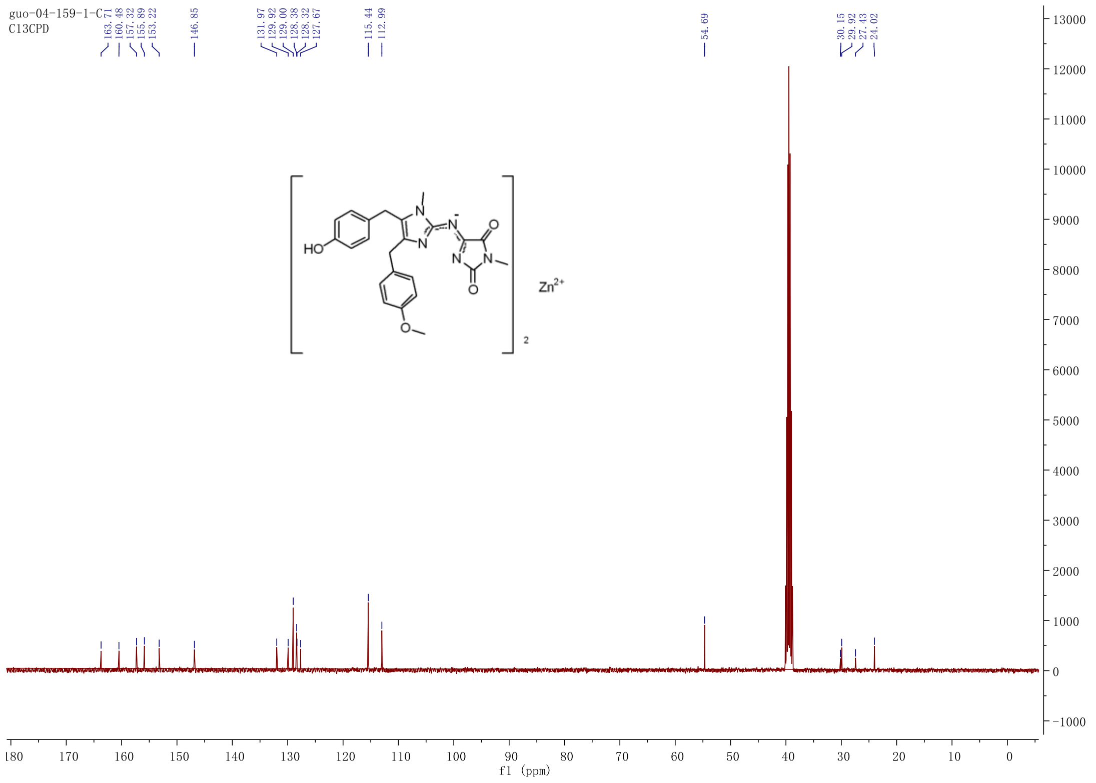

^{13}C NMR spectrum of **2e**

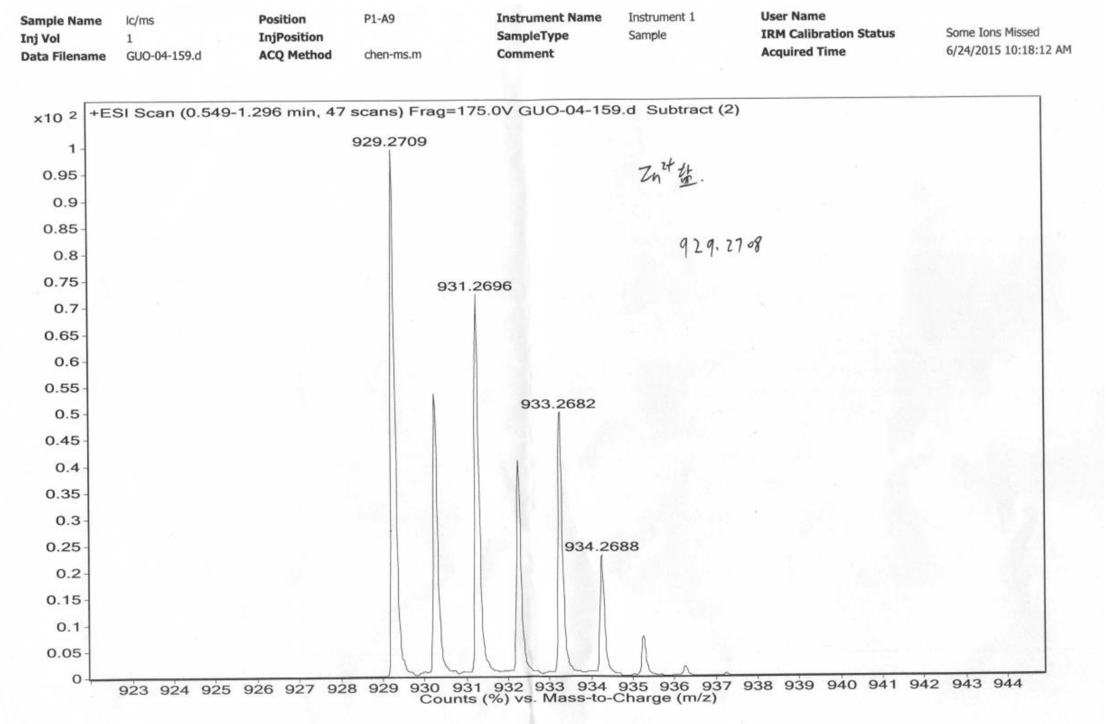

HRMS spectrum of **2e**

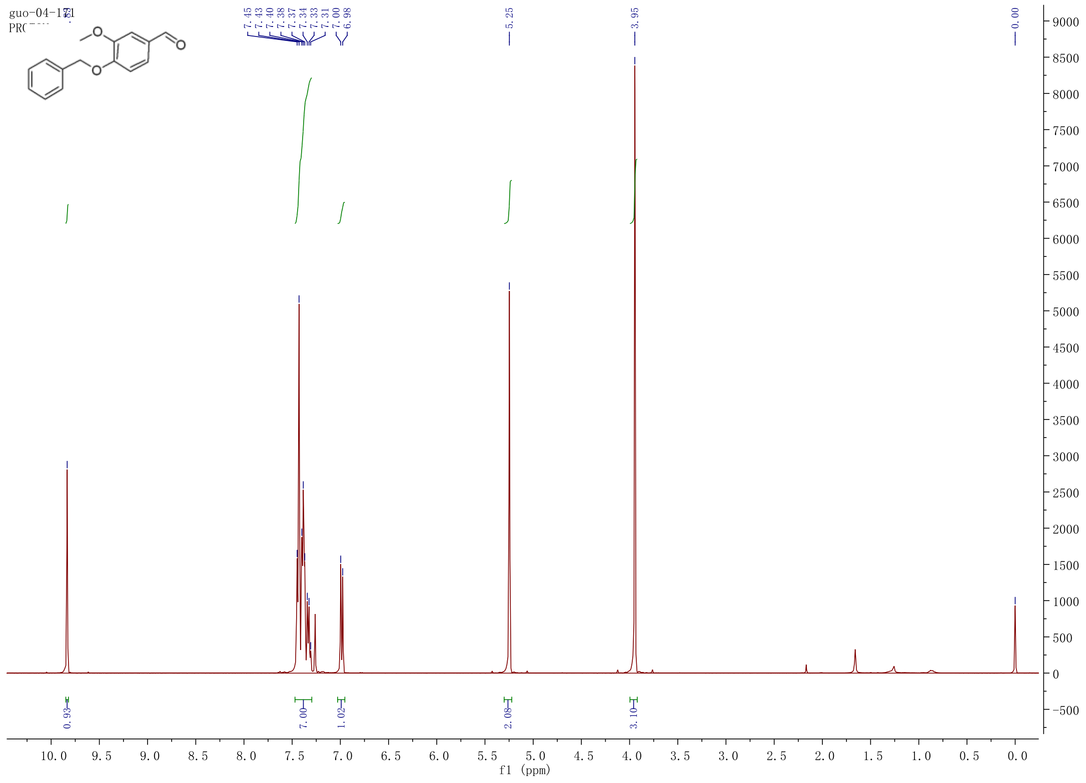

^{1}H NMR spectrum of **4a**

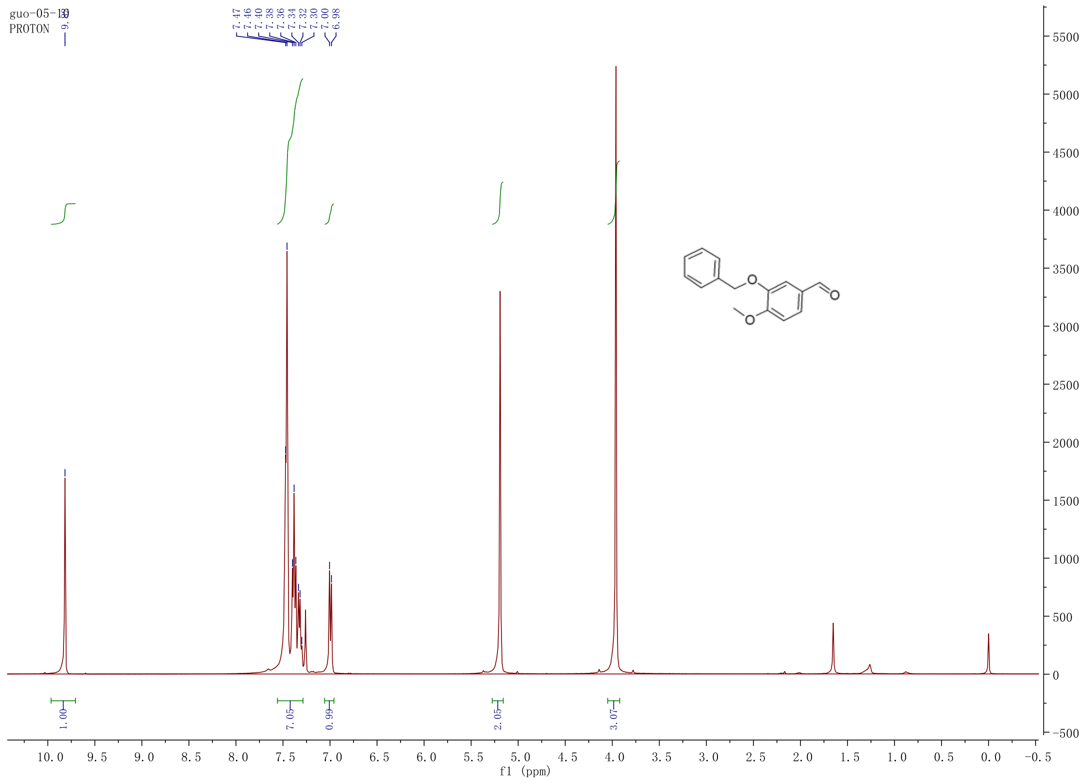

^{1}H NMR spectrum of **4b**

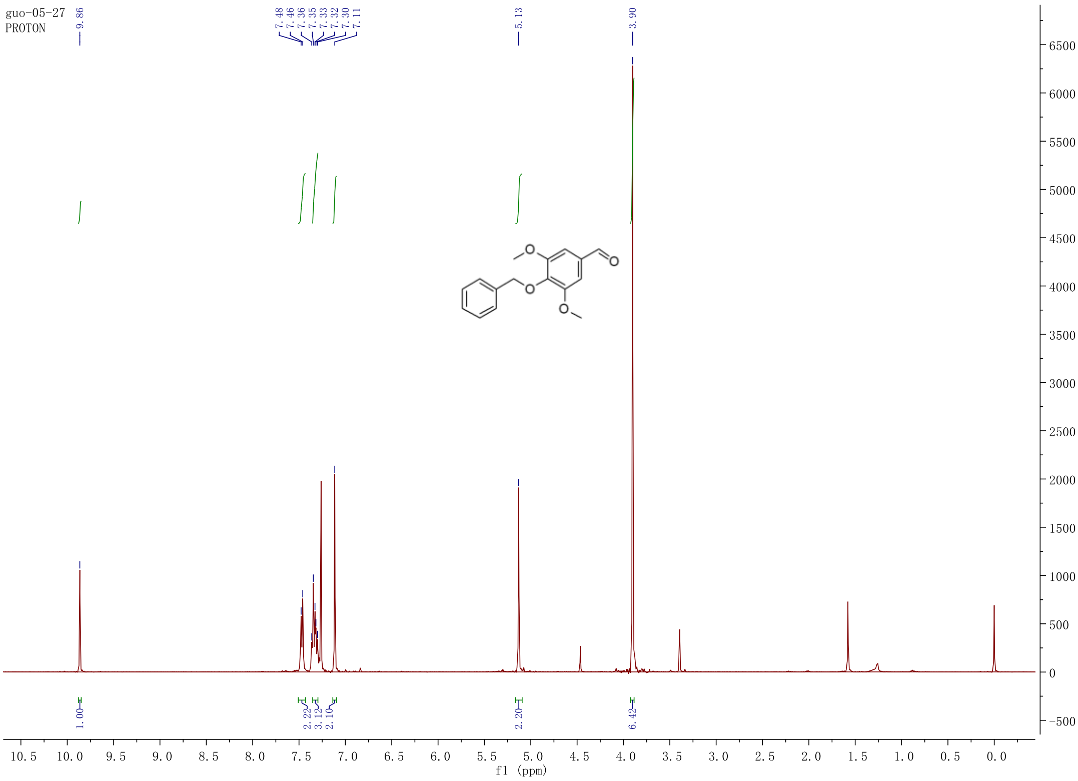

^{1}H NMR spectrum of **4c**

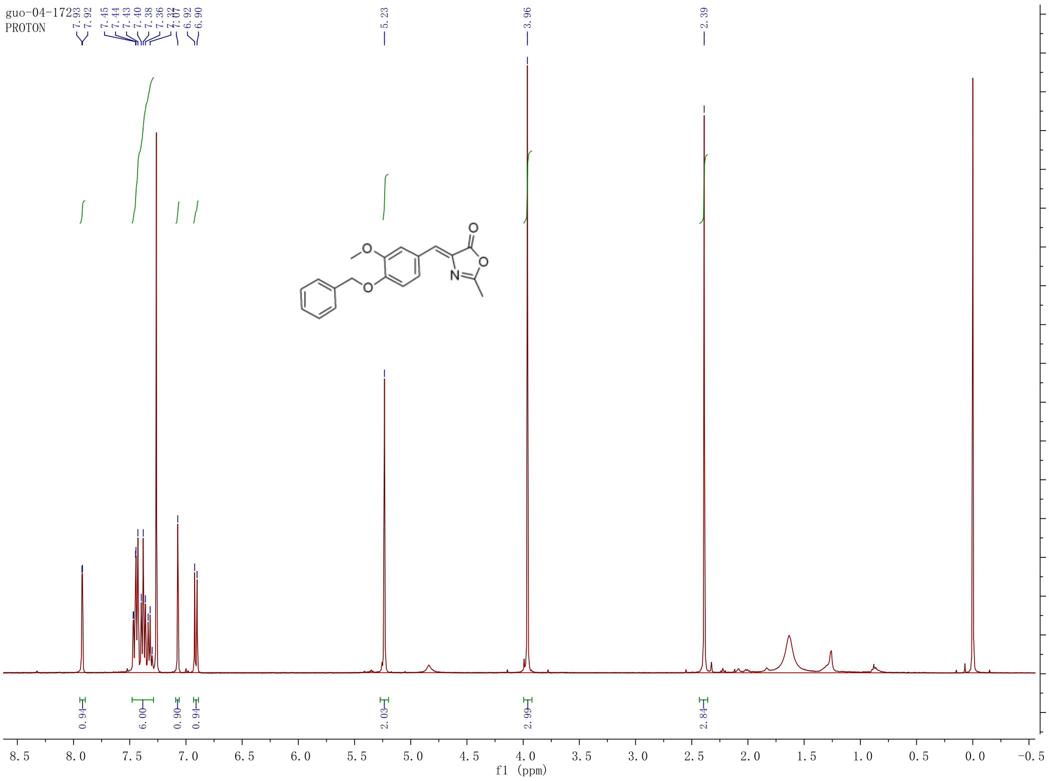

^{1}H NMR spectrum of **5a**

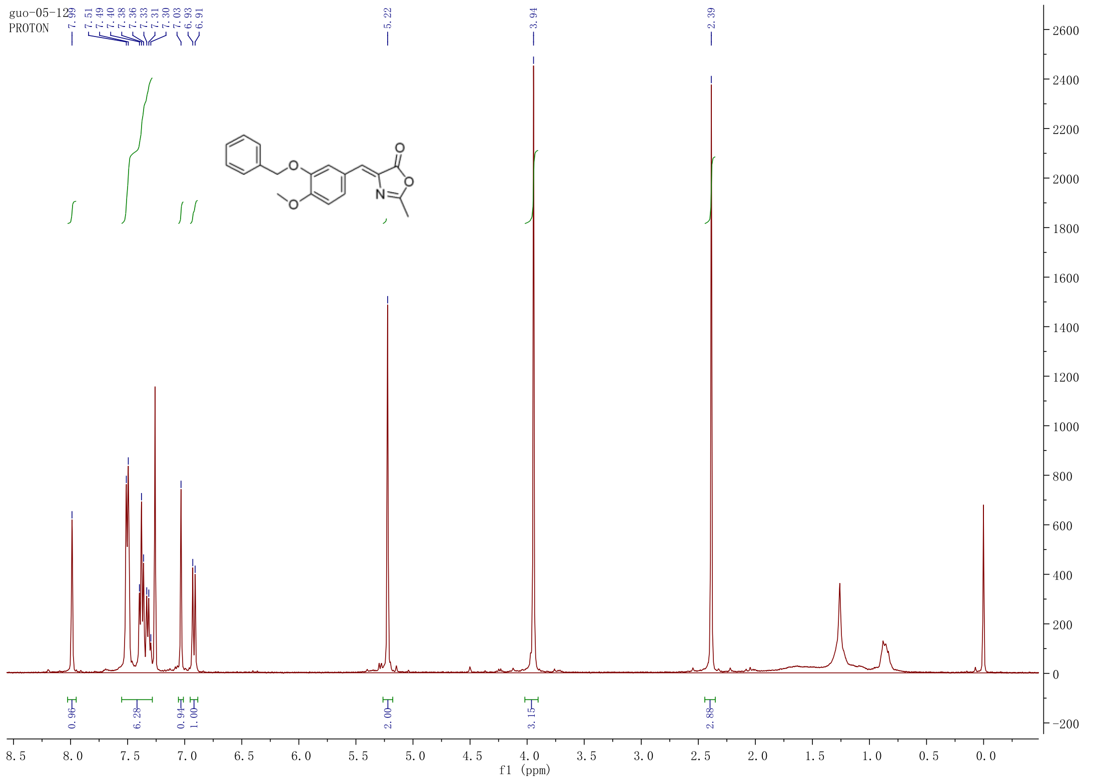

^{1}H NMR spectrum of **5b**

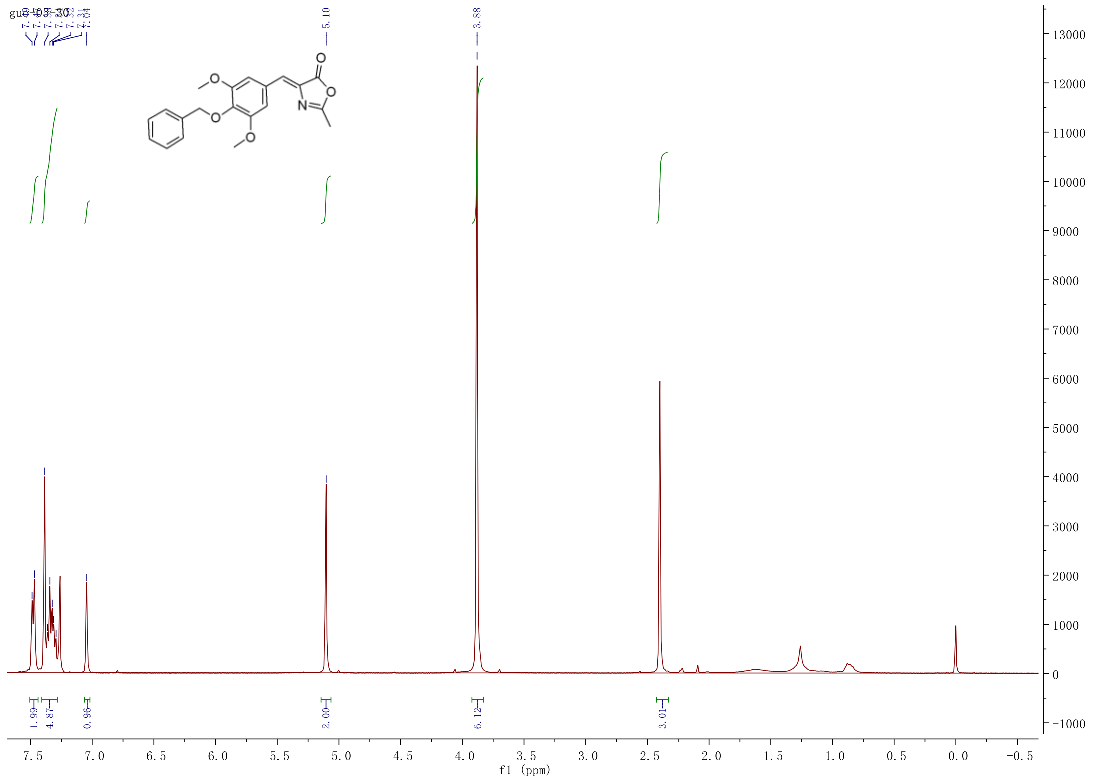

^{1}H NMR spectrum of **5c**

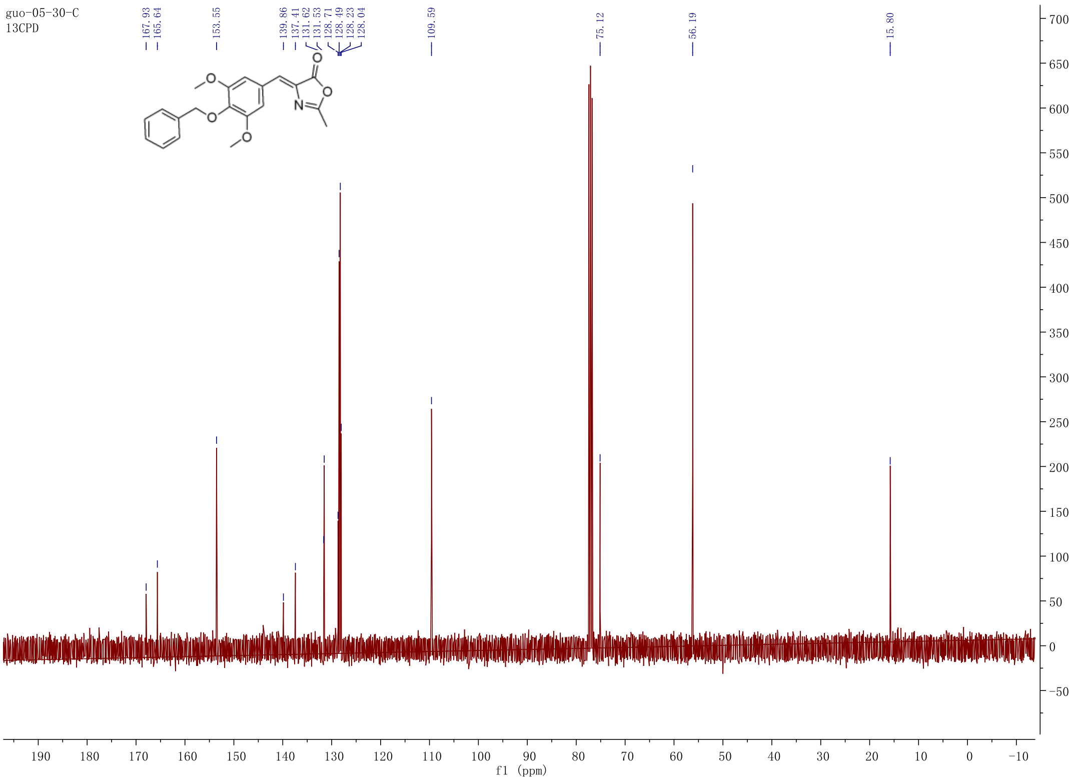

^{13}C NMR spectrum of **5c**

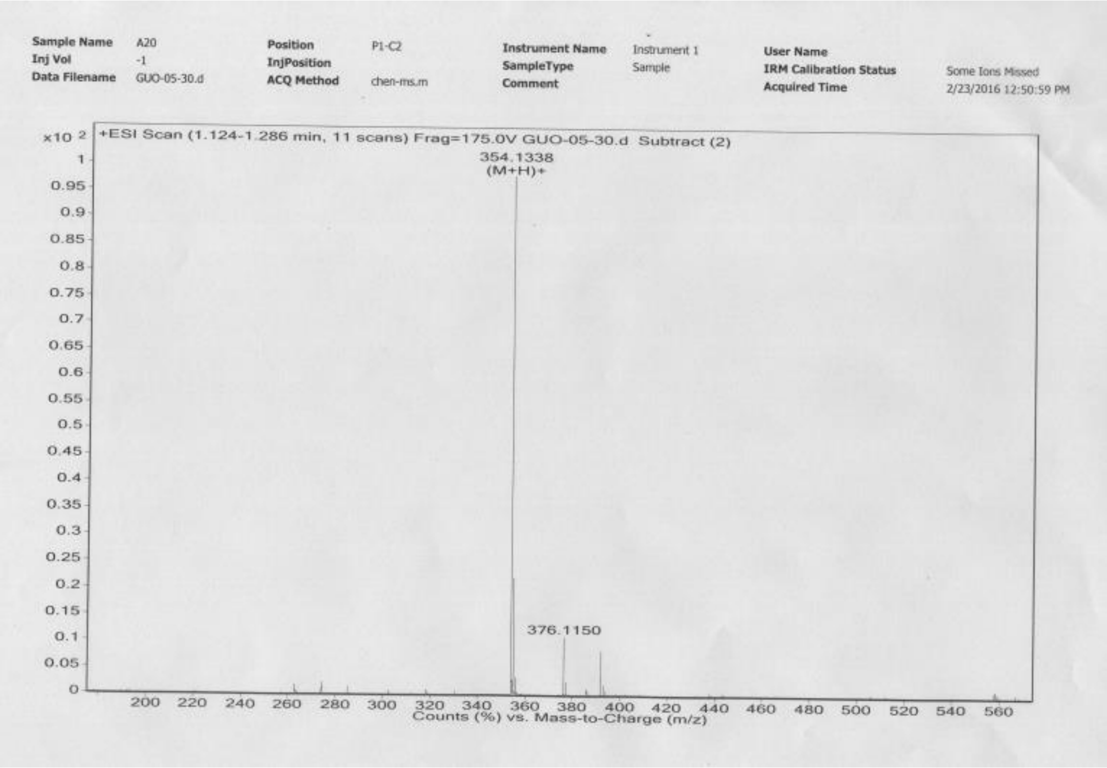

HRMS spectrum of **5c**

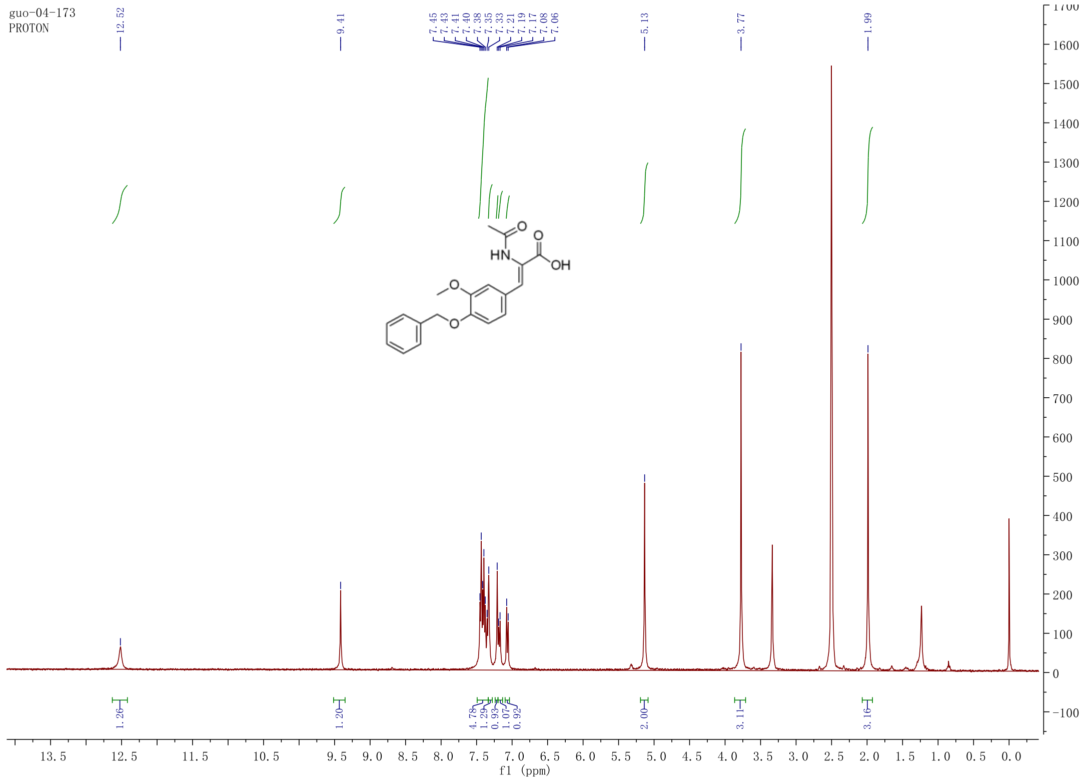

^{1}H NMR spectrum of **6a**

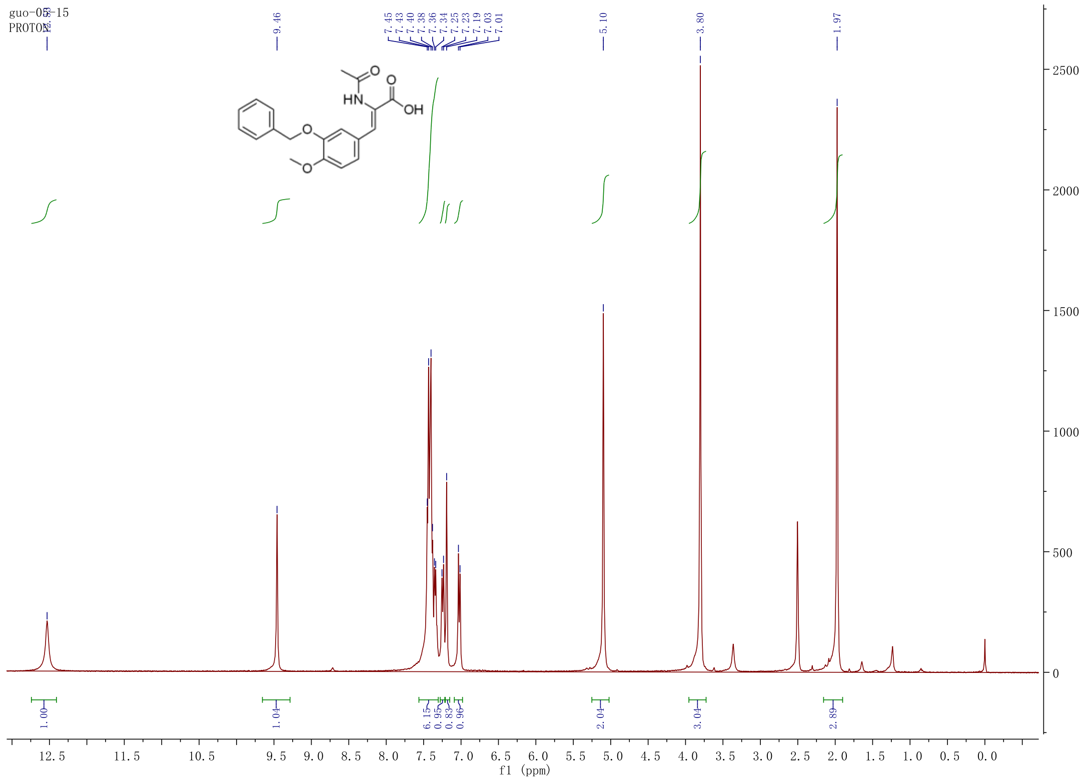

^{1}H NMR spectrum of **6b**

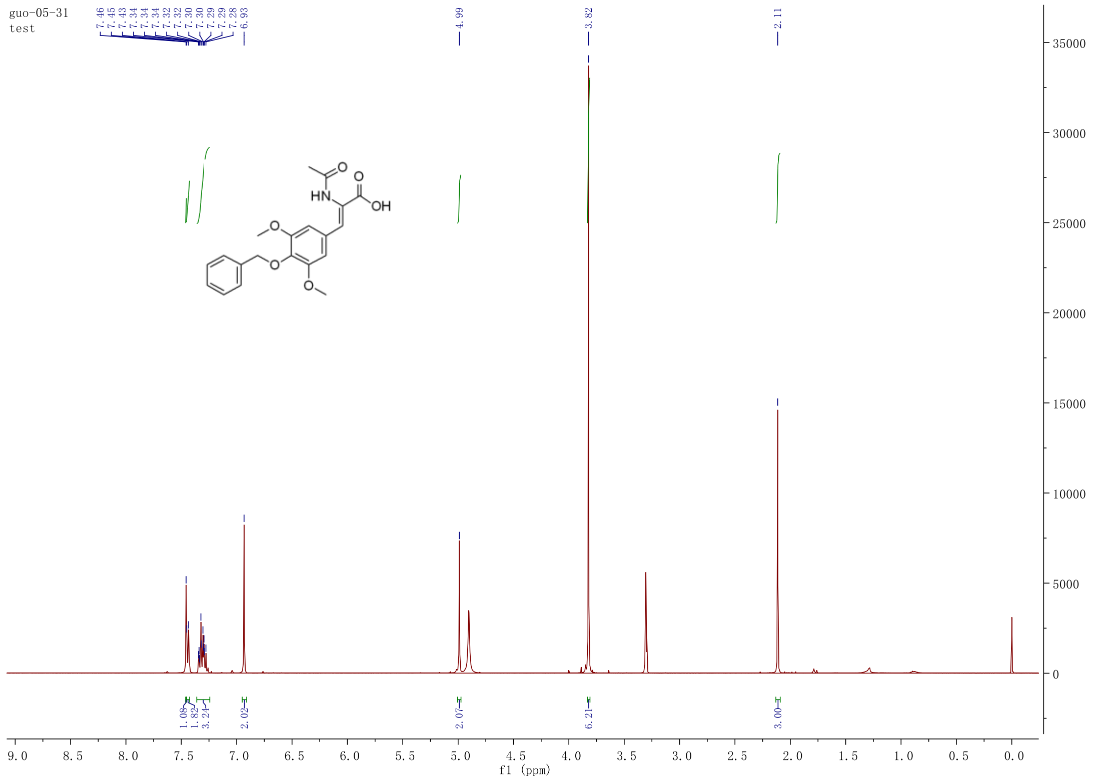

^{1}H NMR spectrum of **6c**

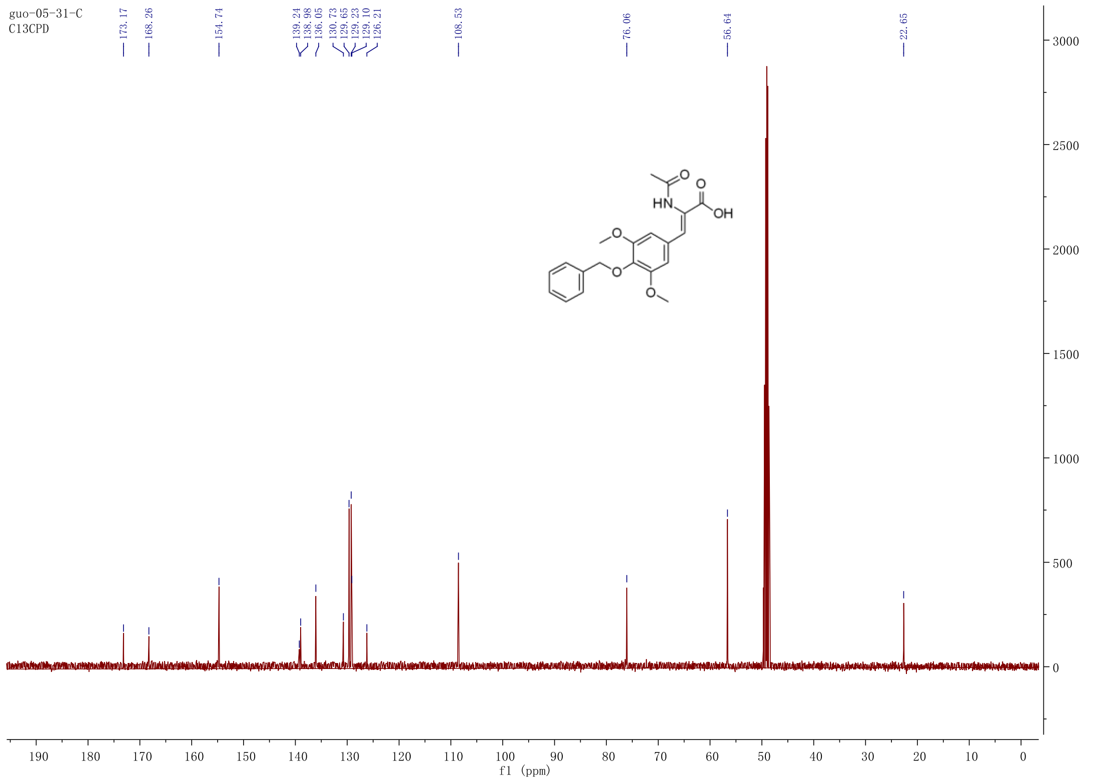

^{13}C NMR spectrum of **6c**

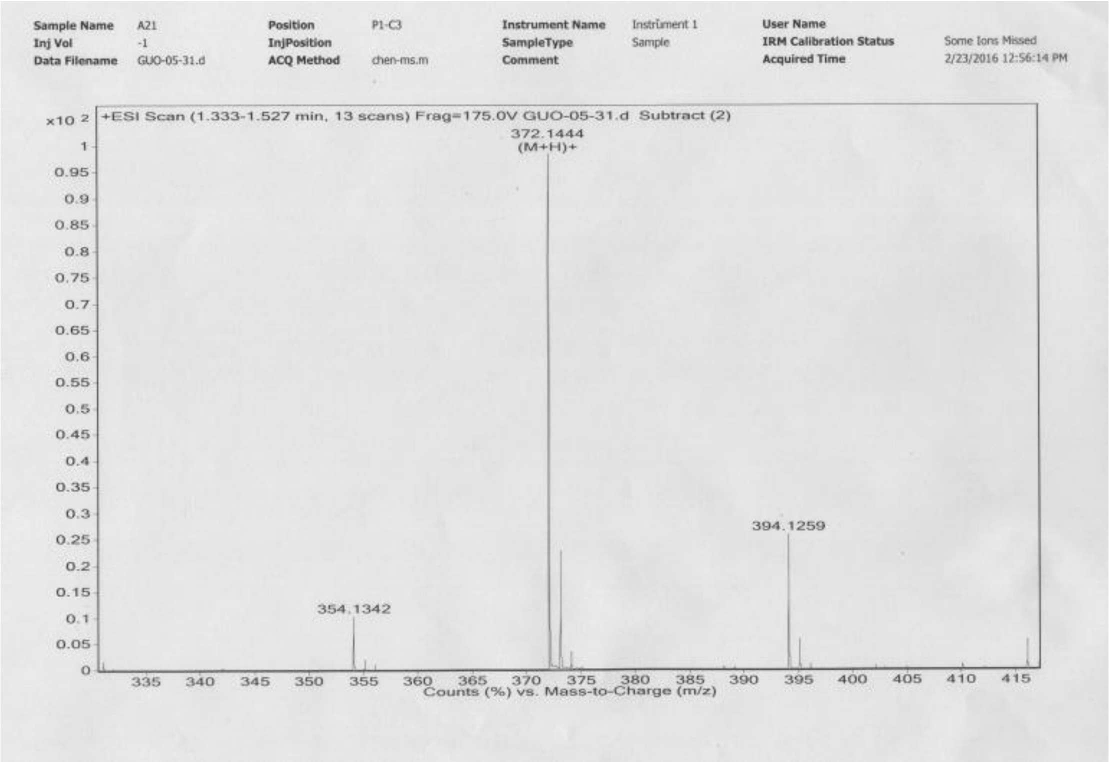

HRMS spectrum of **6c**

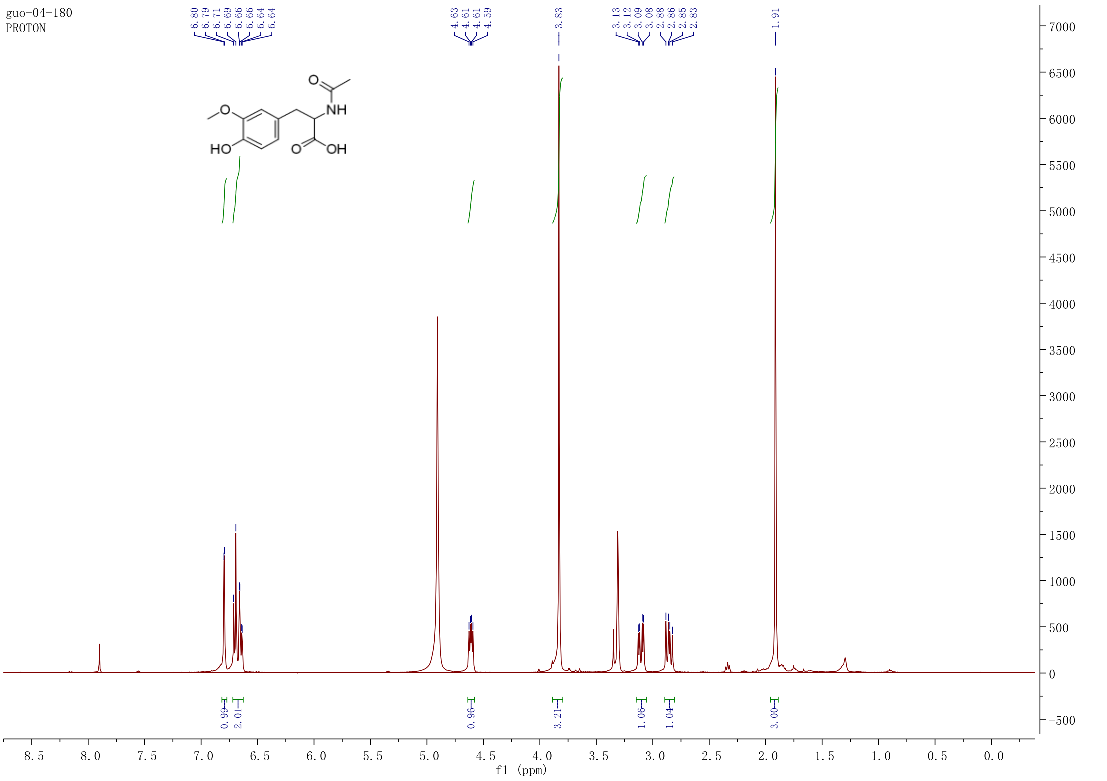

^{1}H NMR spectrum of **7a**

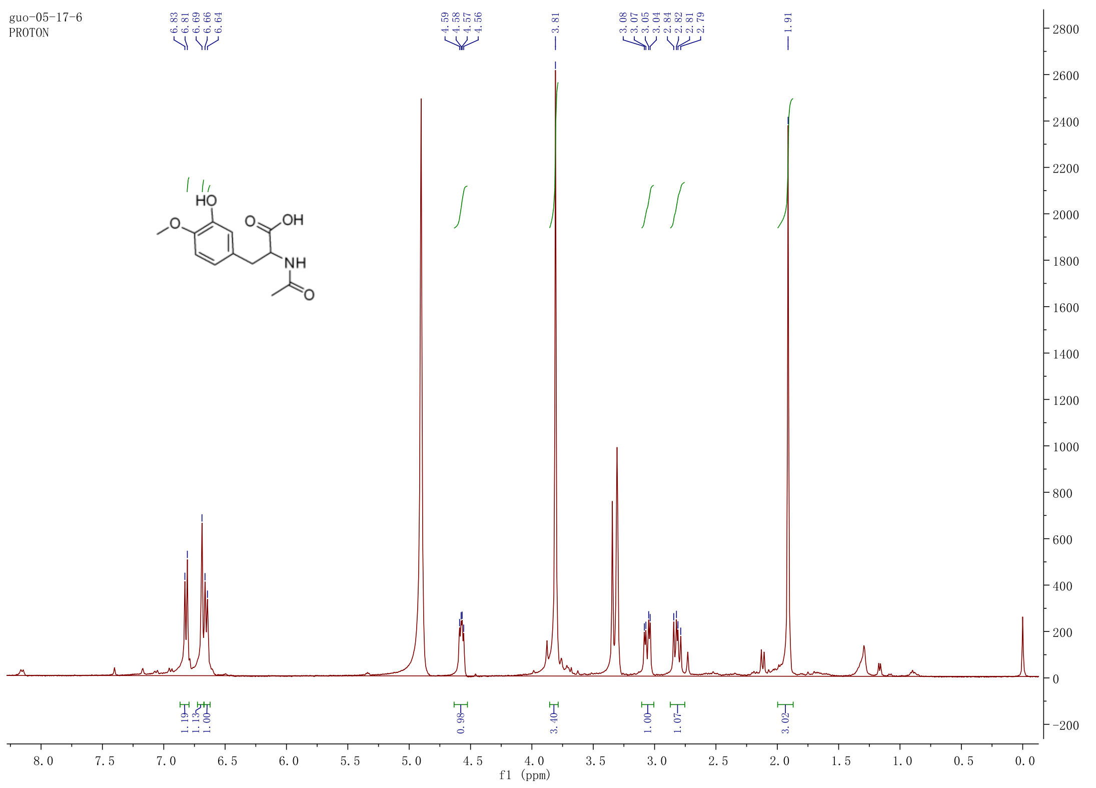

^{1}H NMR spectrum of **7b**

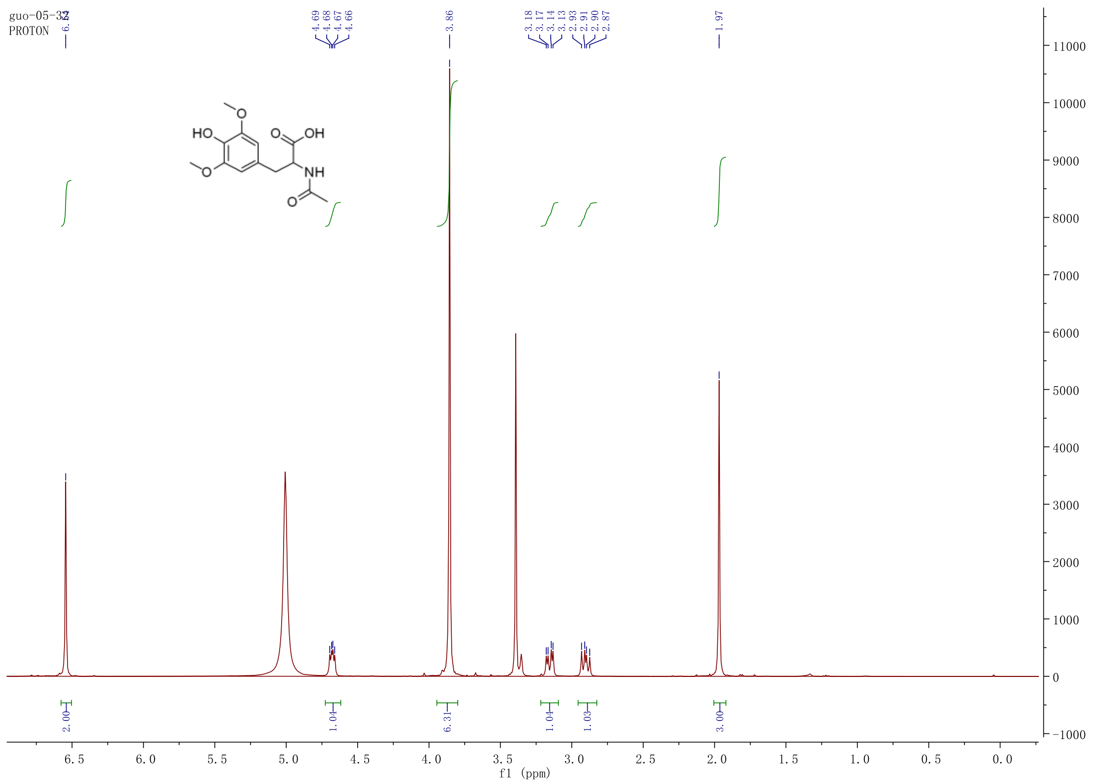

^{1}H NMR spectrum of **7c**

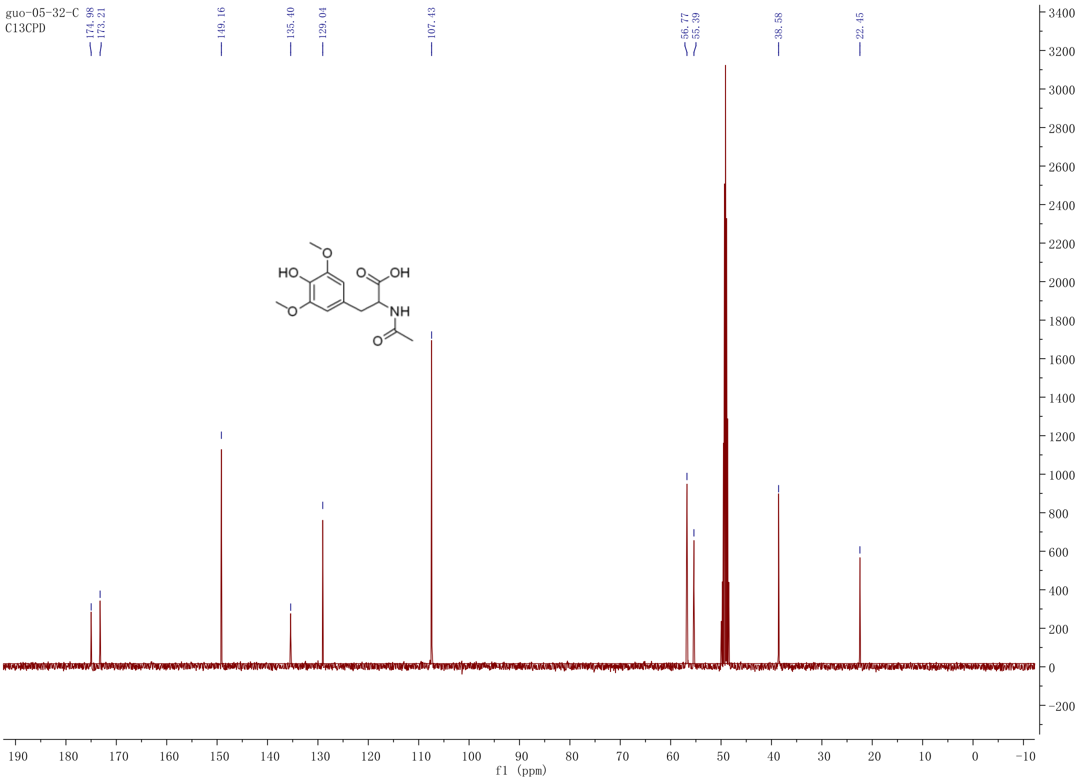

^{13}C NMR spectrum of **7c**

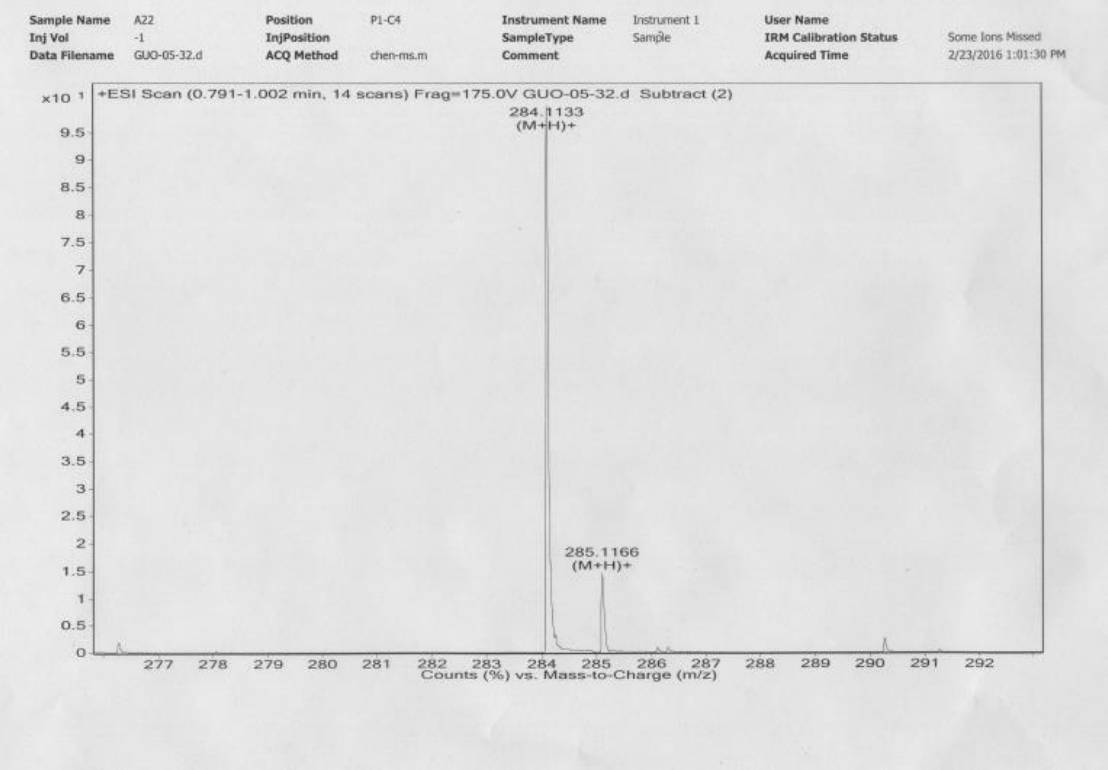

HRMS spectrum of **7c**

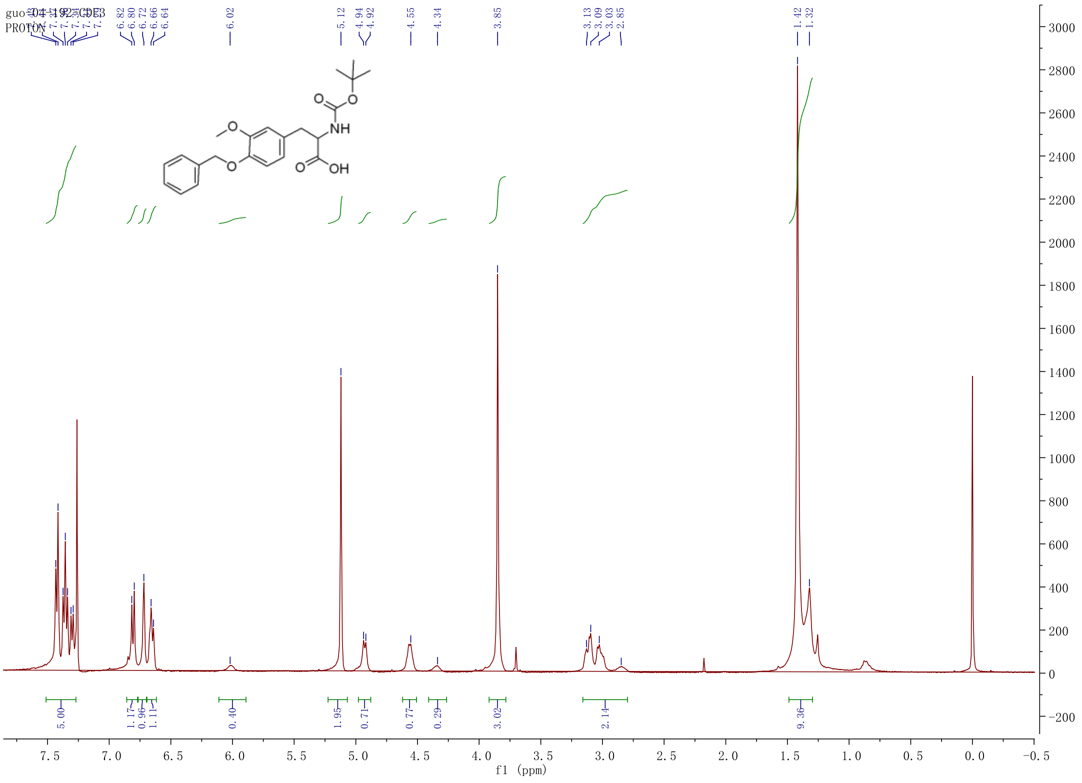

^{1}H NMR spectrum of **10a**

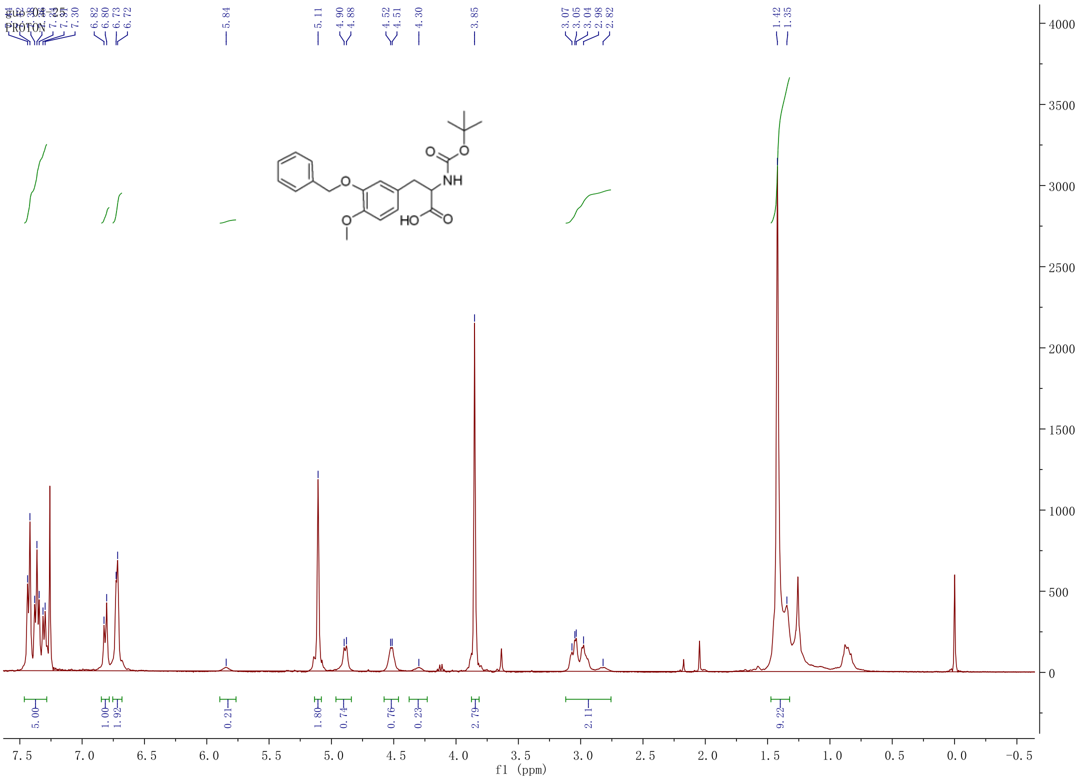

^{1}H NMR spectrum of **10b**

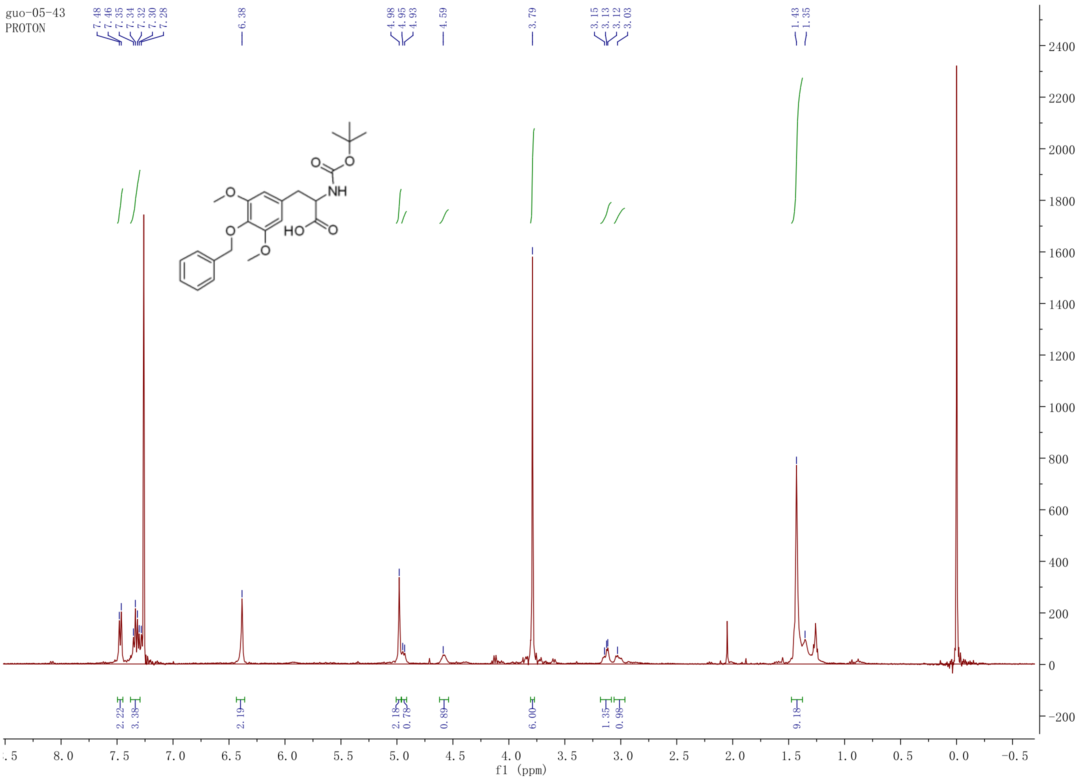

^{1}H NMR spectrum of **10c**

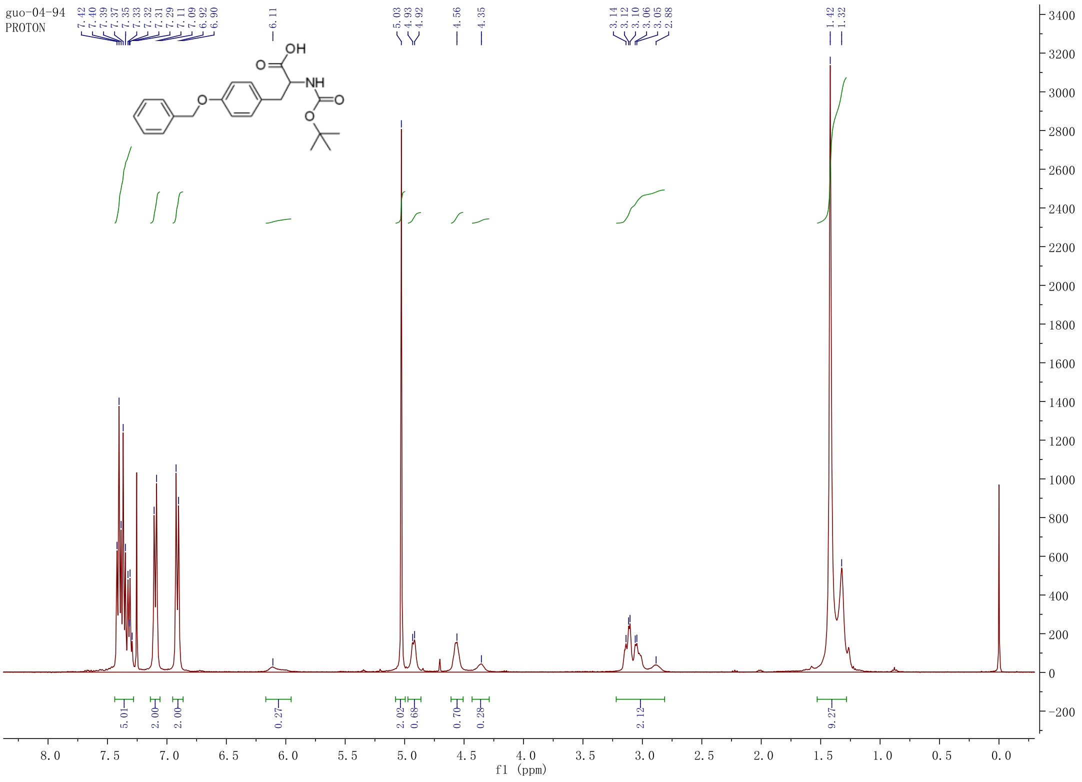

^{1}H NMR spectrum of **10d**

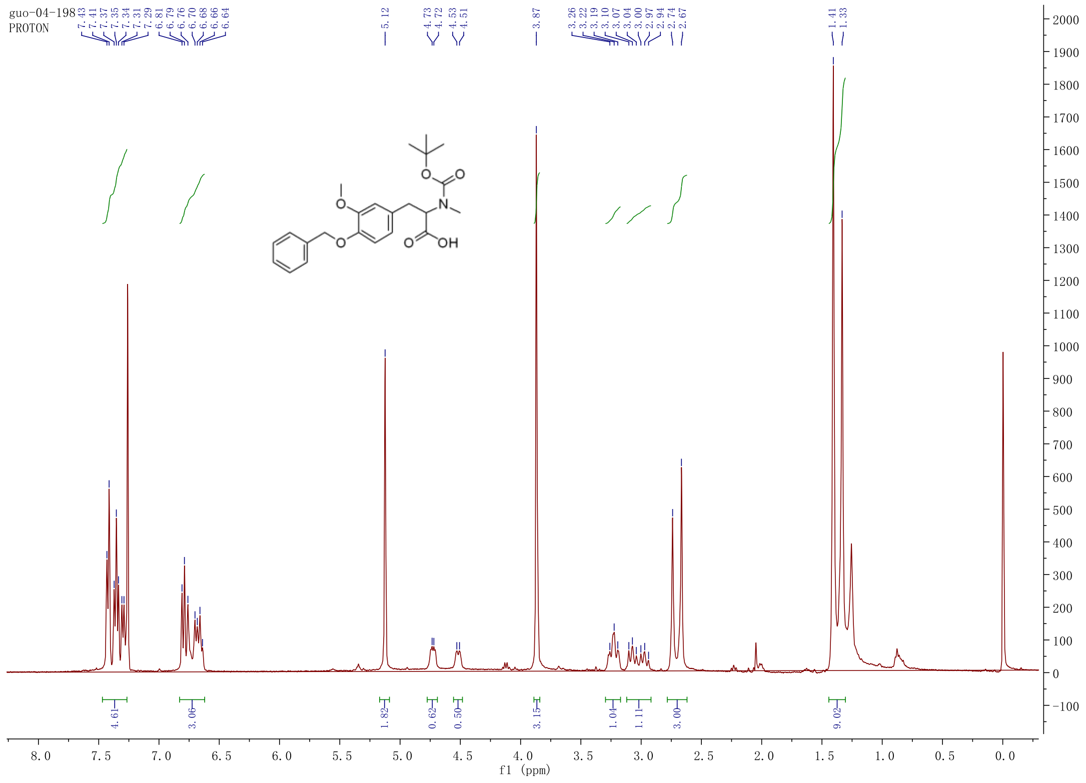

^{1}H NMR spectrum of **11a**

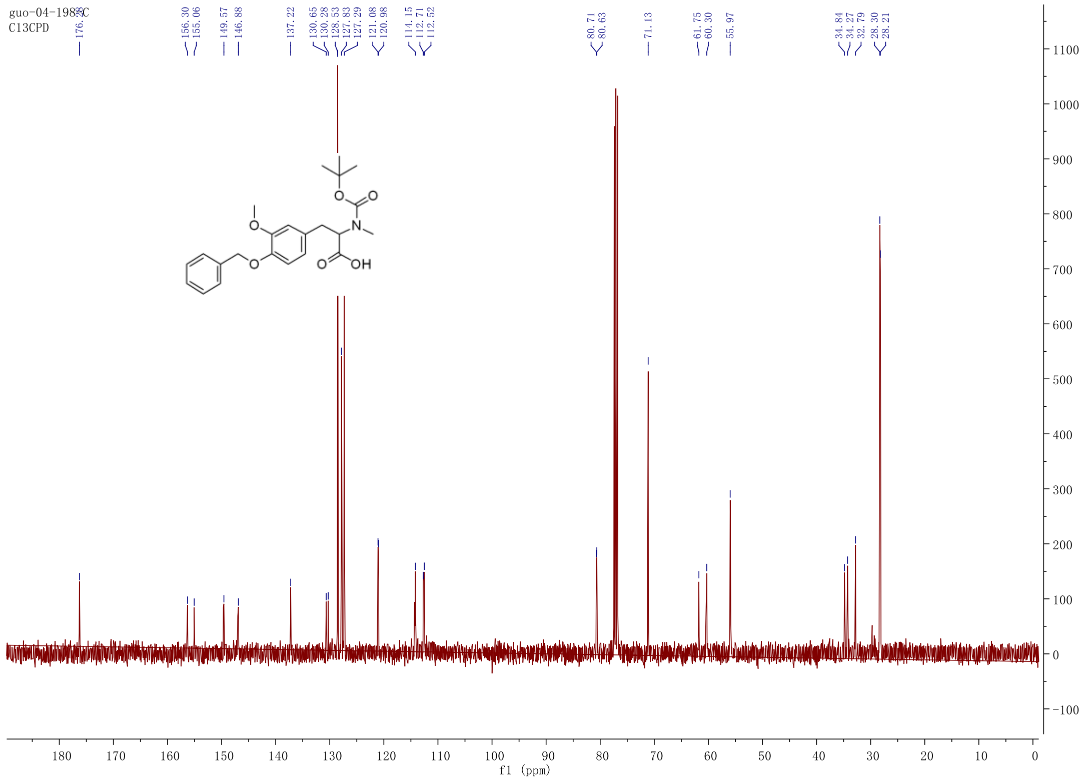

^{13}C NMR spectrum of **11a**

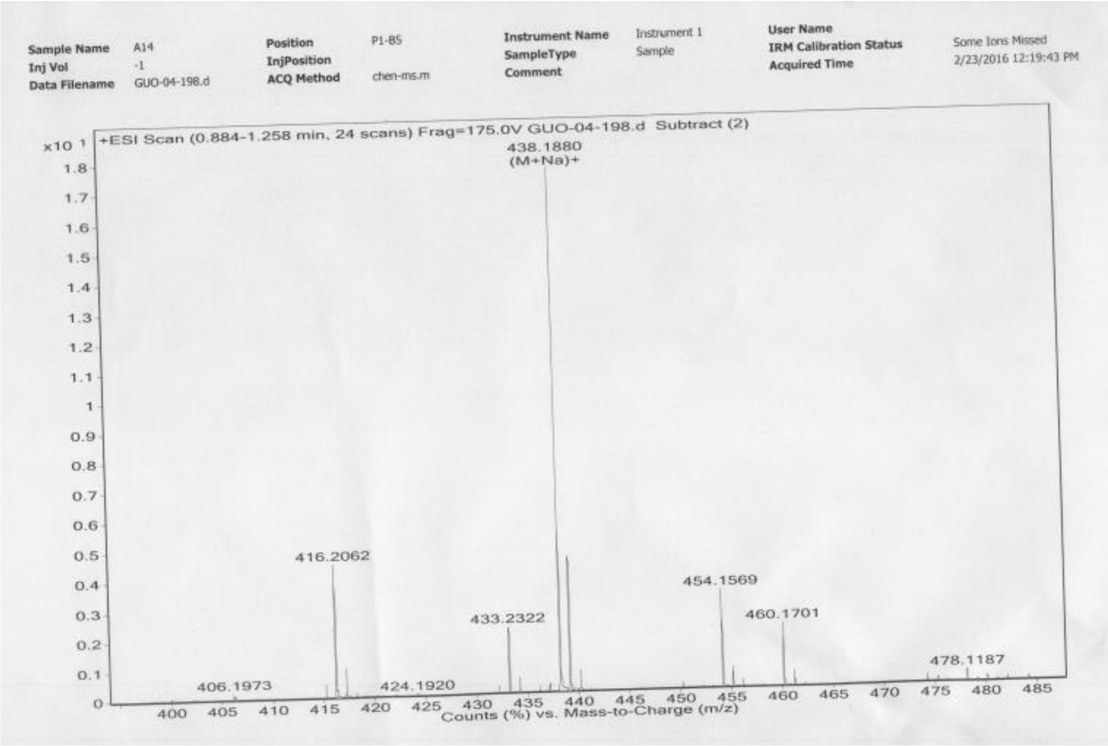

HRMS spectrum of **11a**

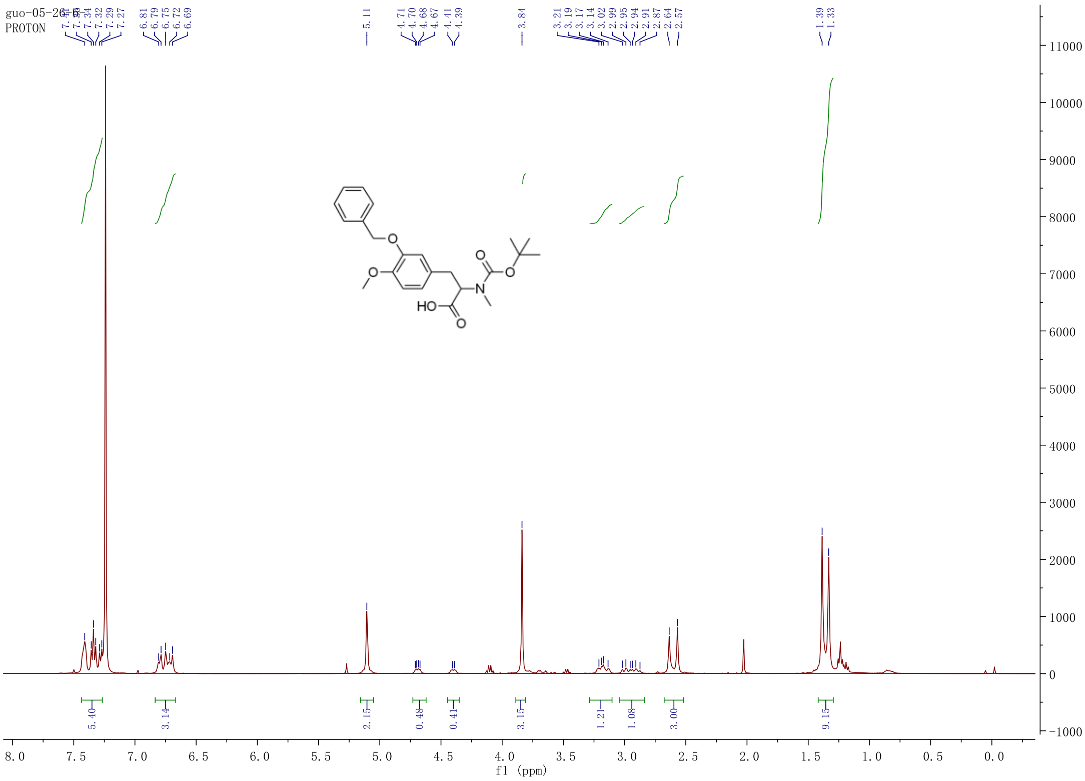

^{1}H NMR spectrum of **11b**

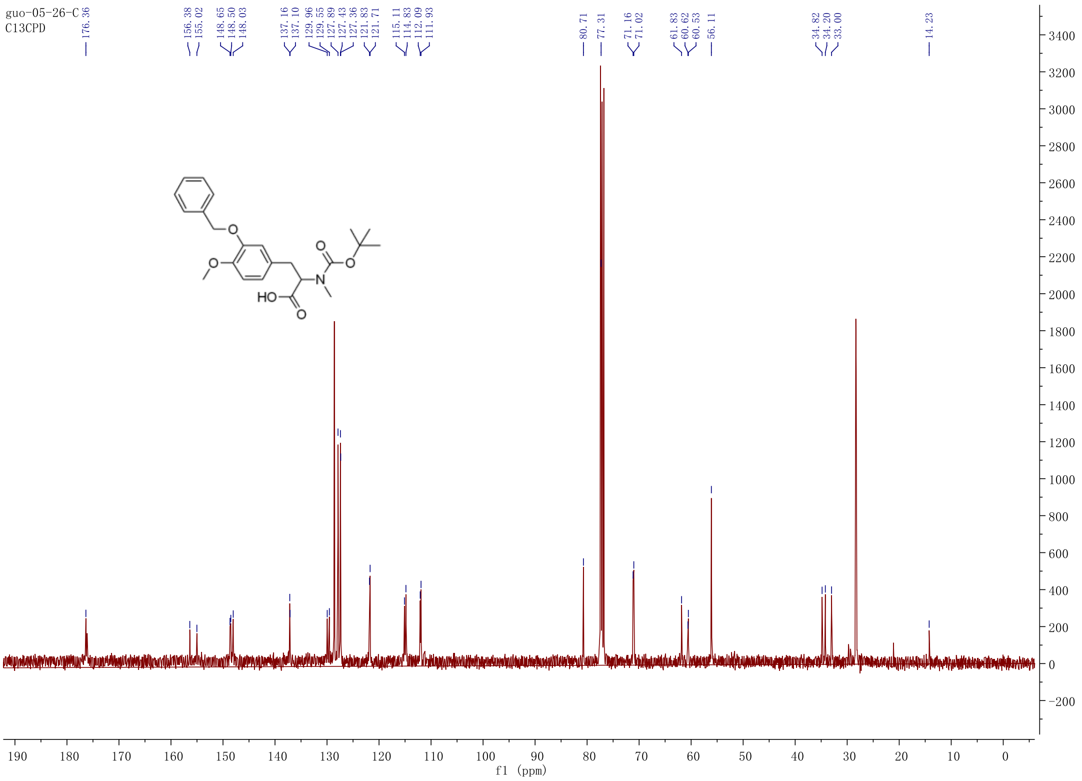

^{13}C NMR spectrum of **11b**

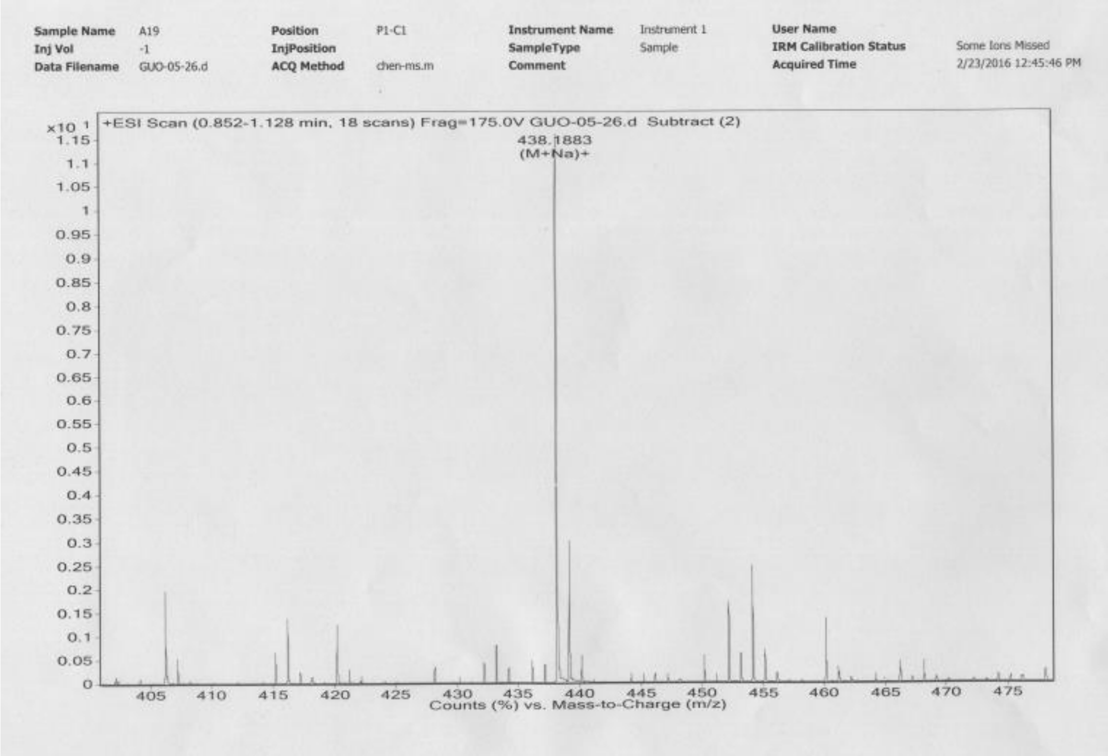

HRMS spectrum of **11b**

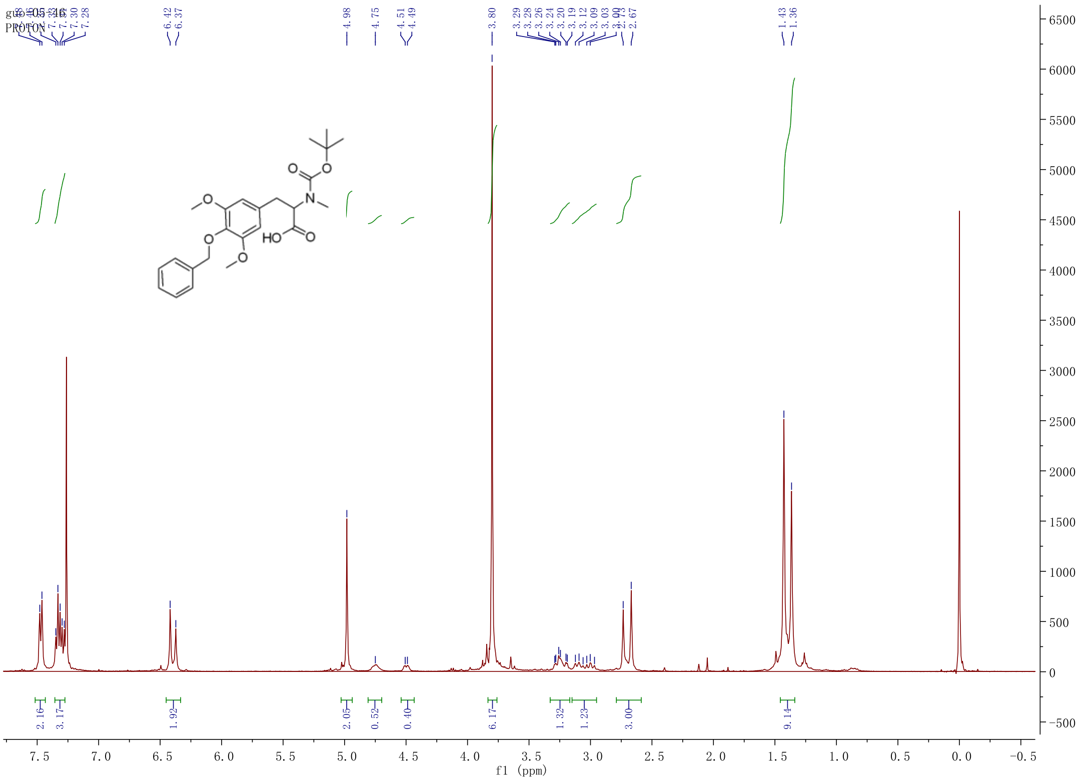

^{1}H NMR spectrum of **11c**

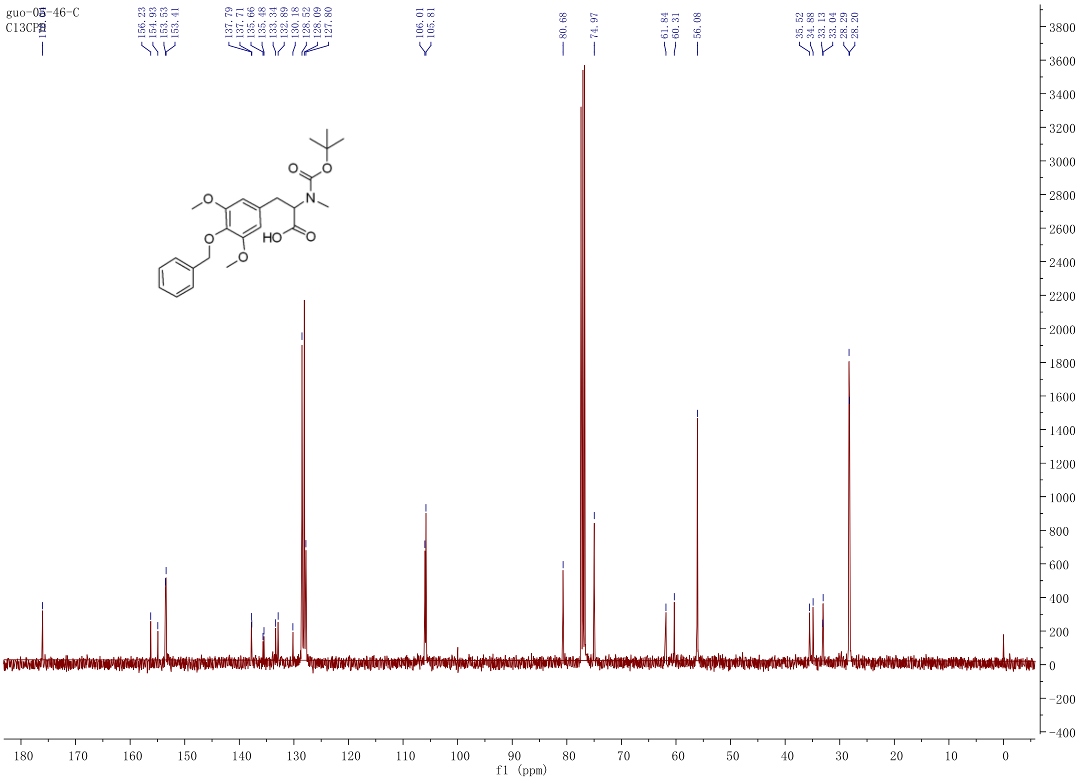

^{13}C NMR spectrum of **11c**

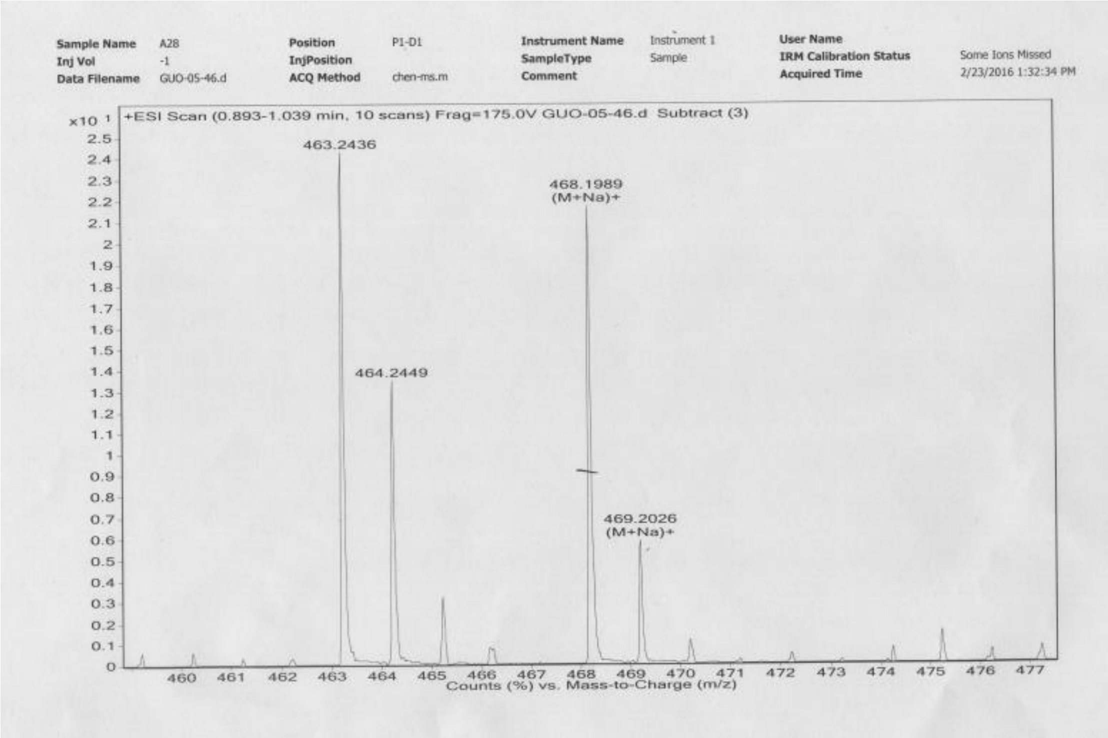

HRMS spectrum of **11c**

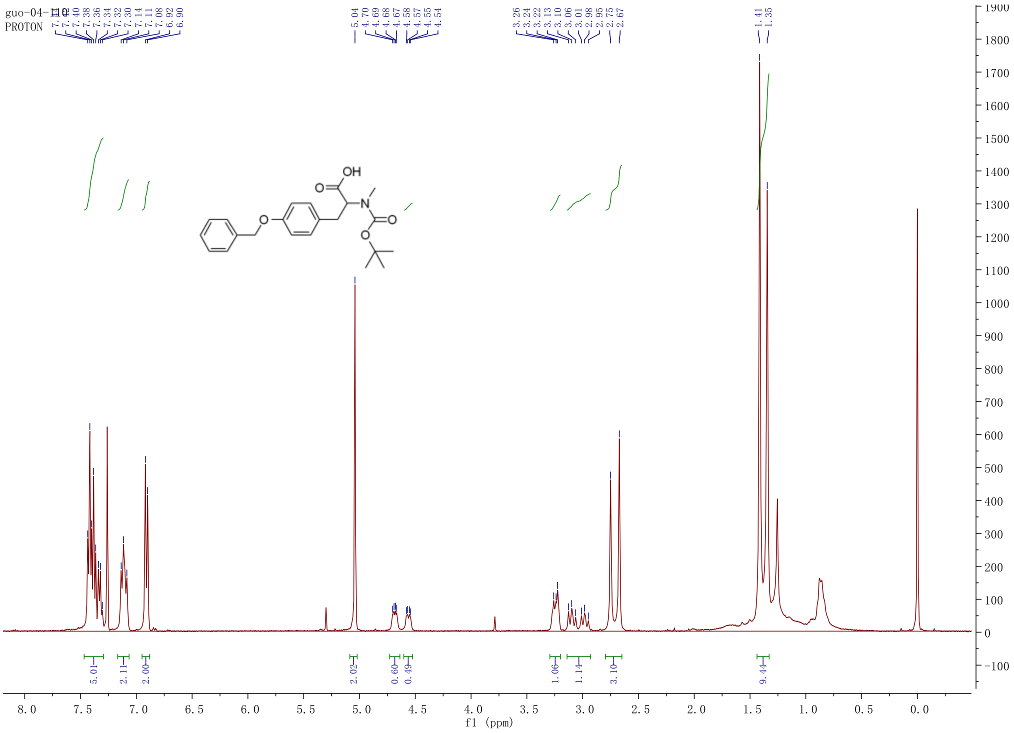

^{1}H NMR spectrum of **11d**

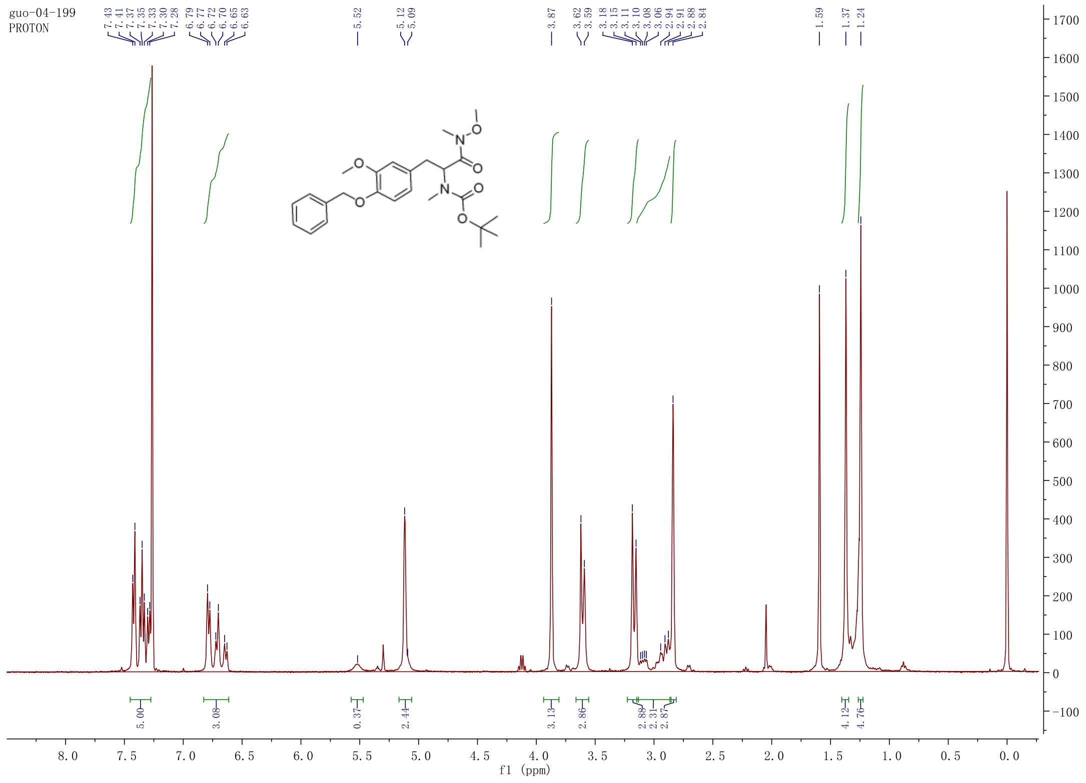

^{1}H NMR spectrum of **12a**

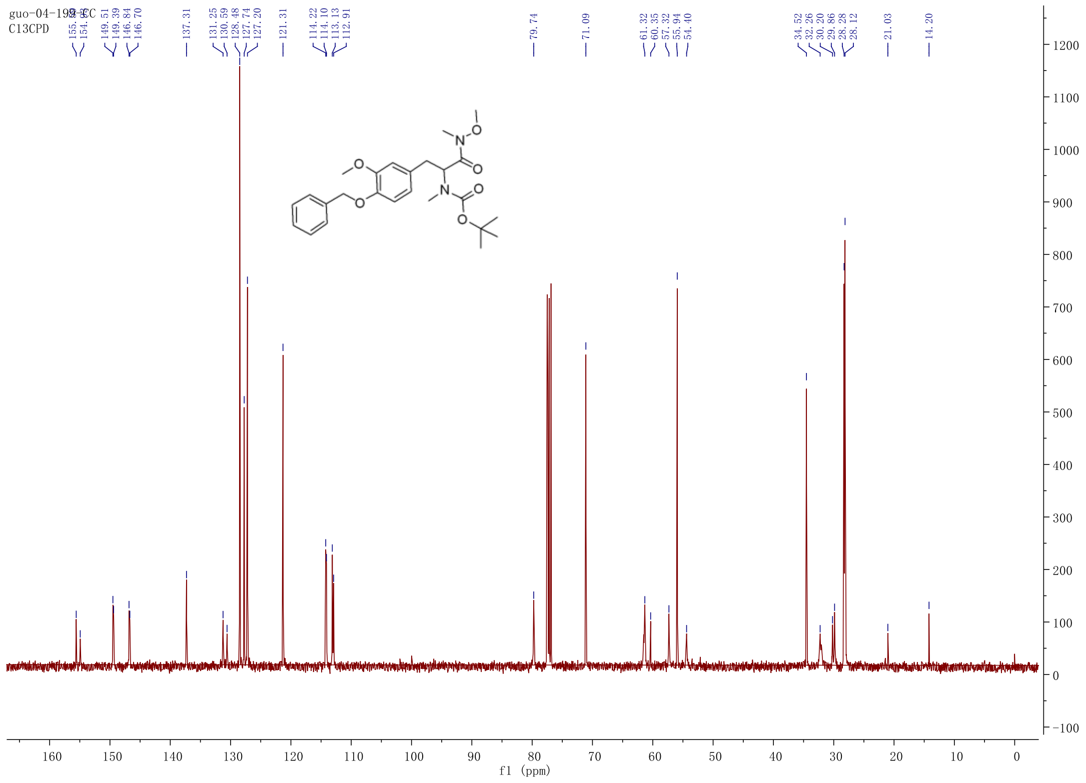

^{13}C NMR spectrum of **12a**

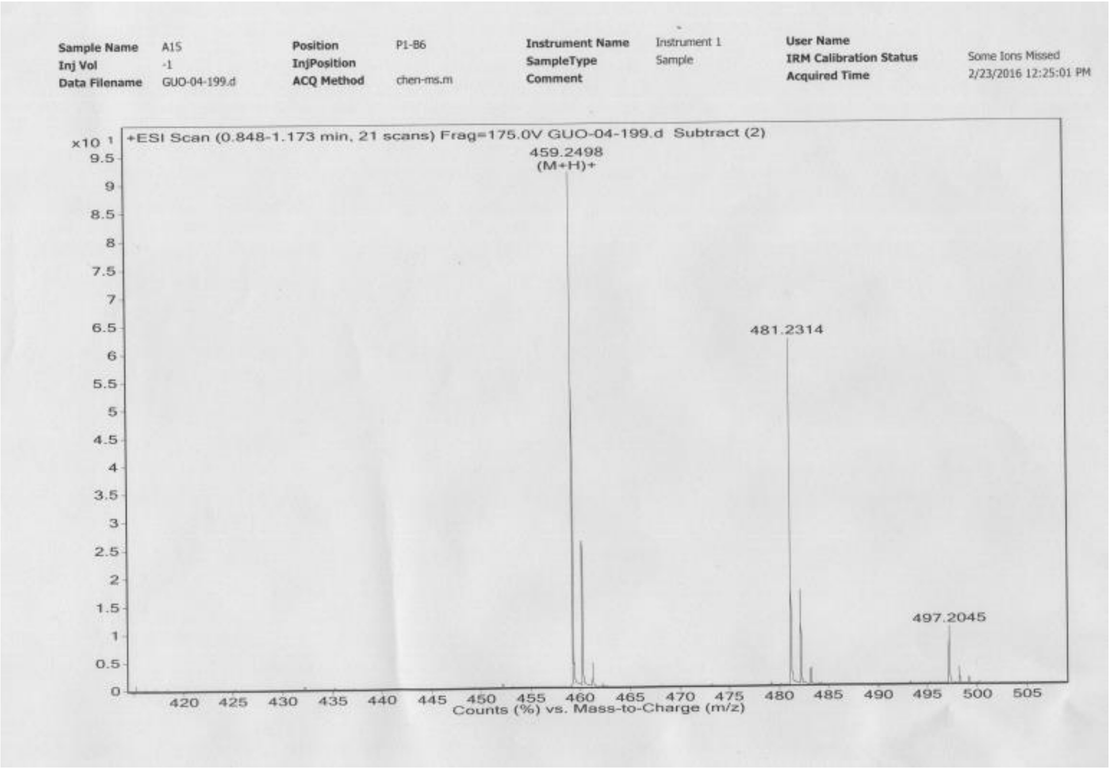

HRMS spectrum of **12a**

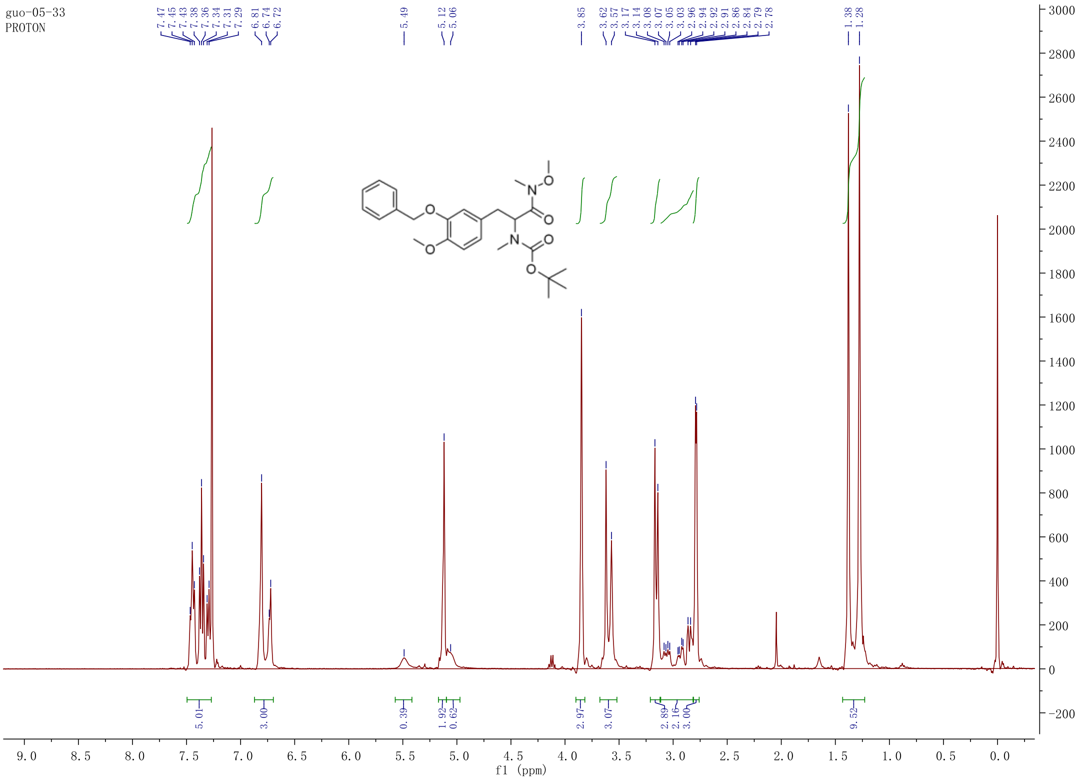

^{1}H NMR spectrum of **12b**

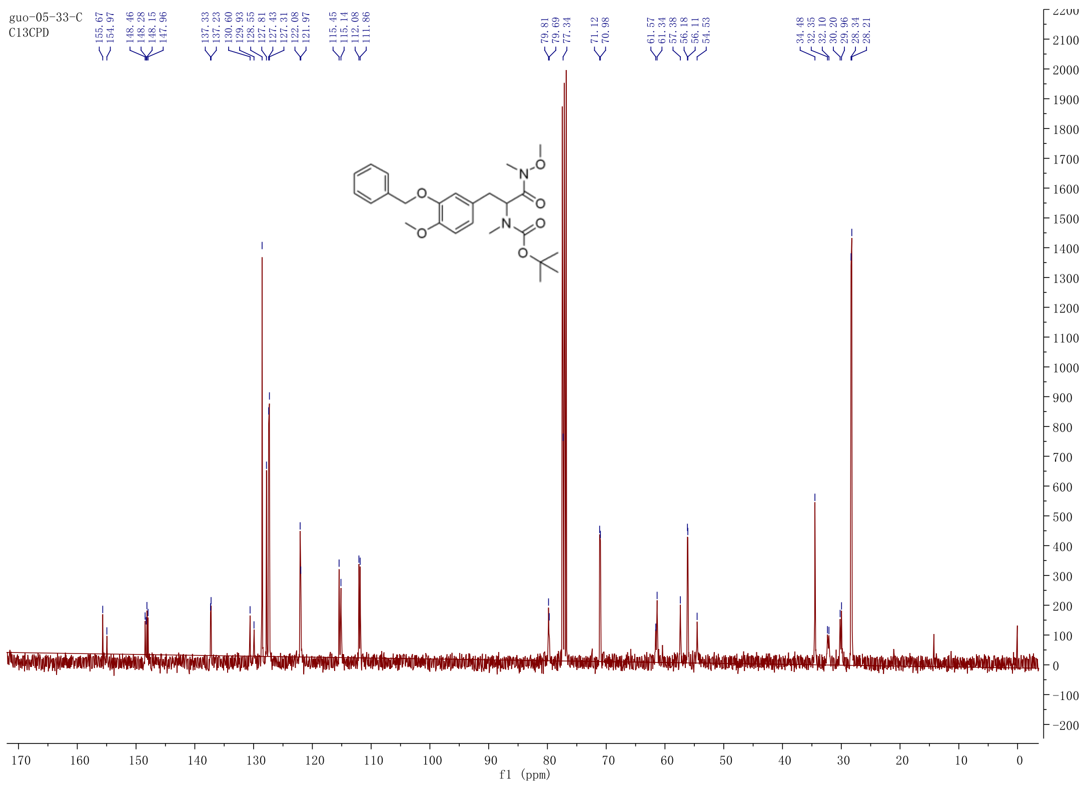

^{13}C NMR spectrum of **12b**

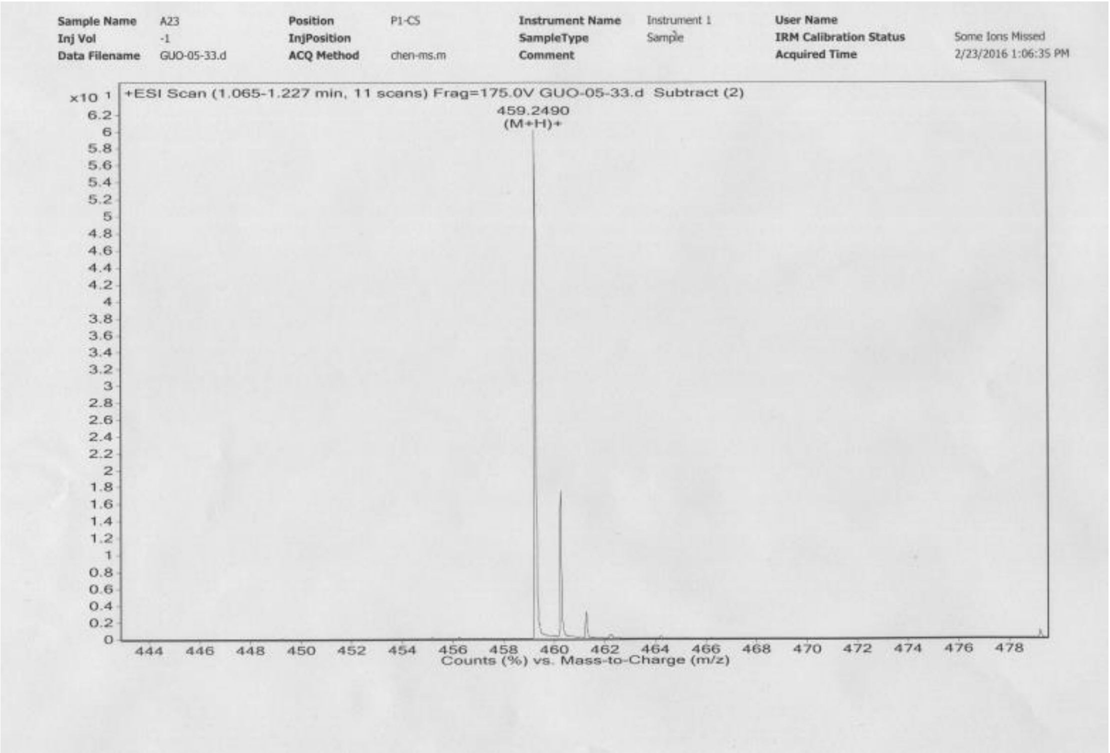

HRMS spectrum of **12b**

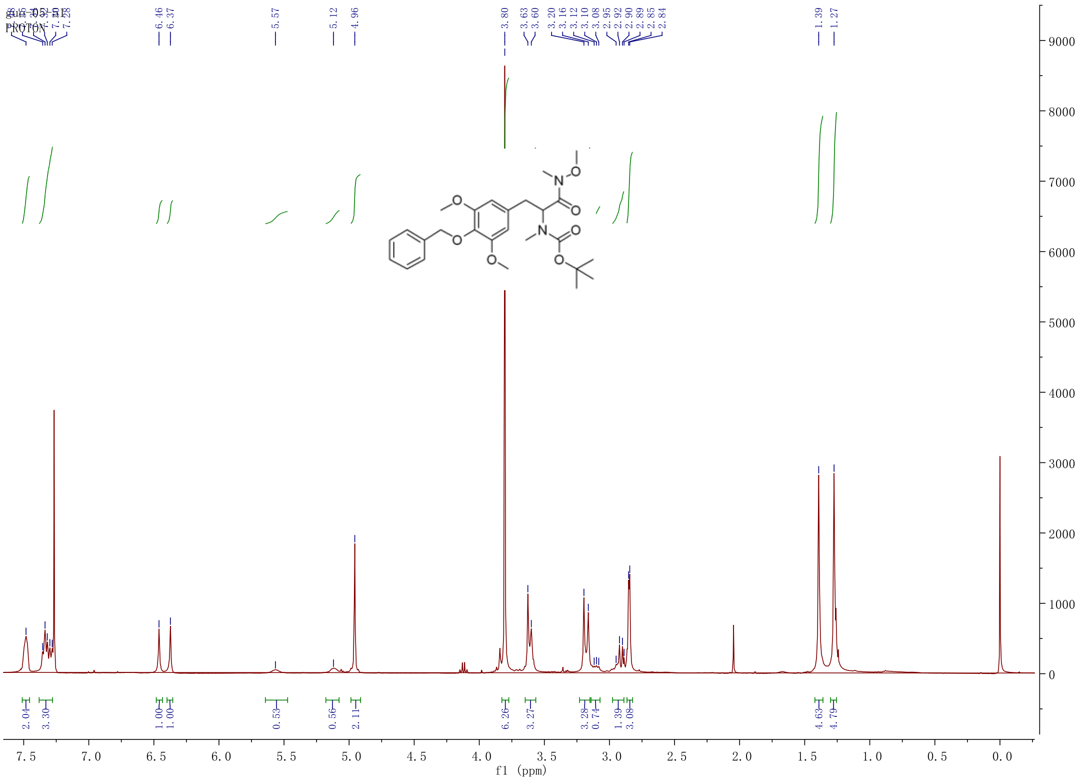

^{1}H NMR spectrum of **12c**

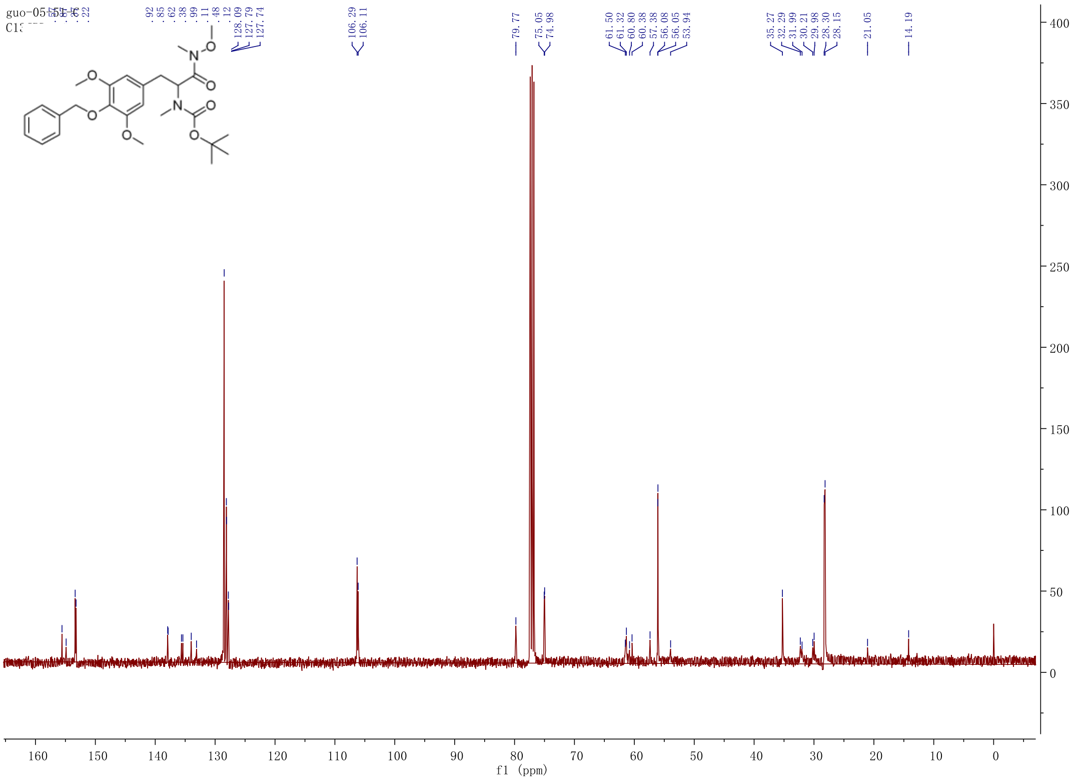

^{13}C NMR spectrum of **12c**

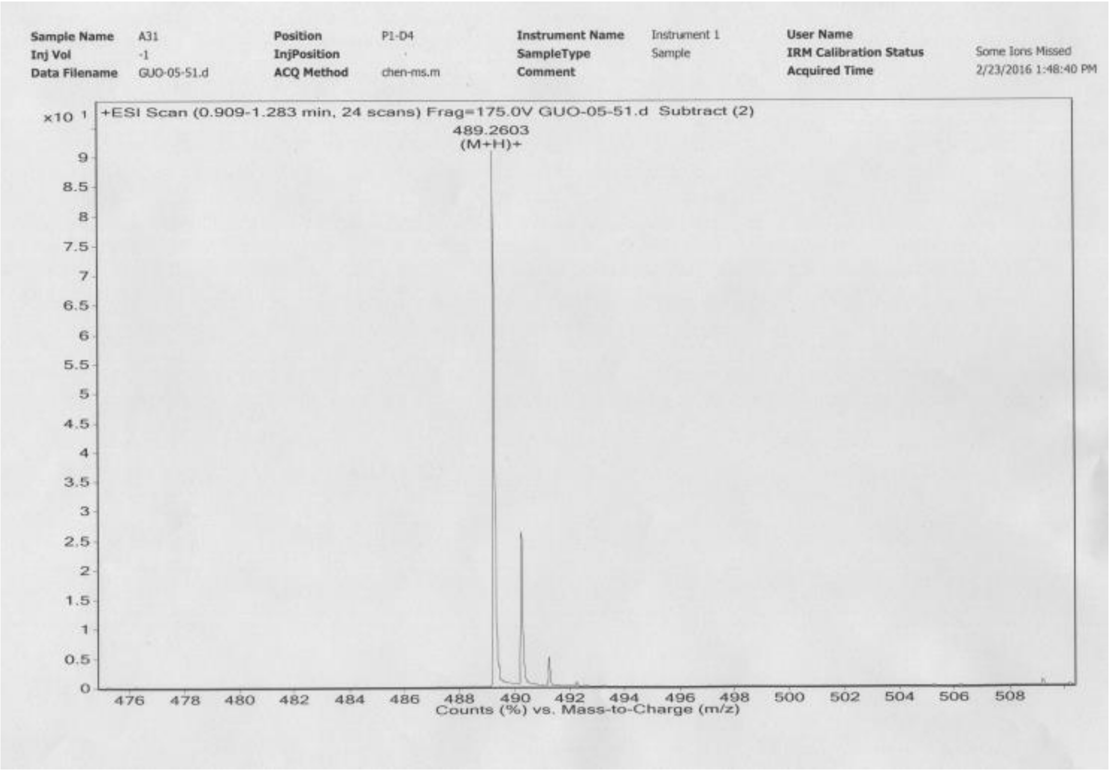

HRMS spectrum of **12c**

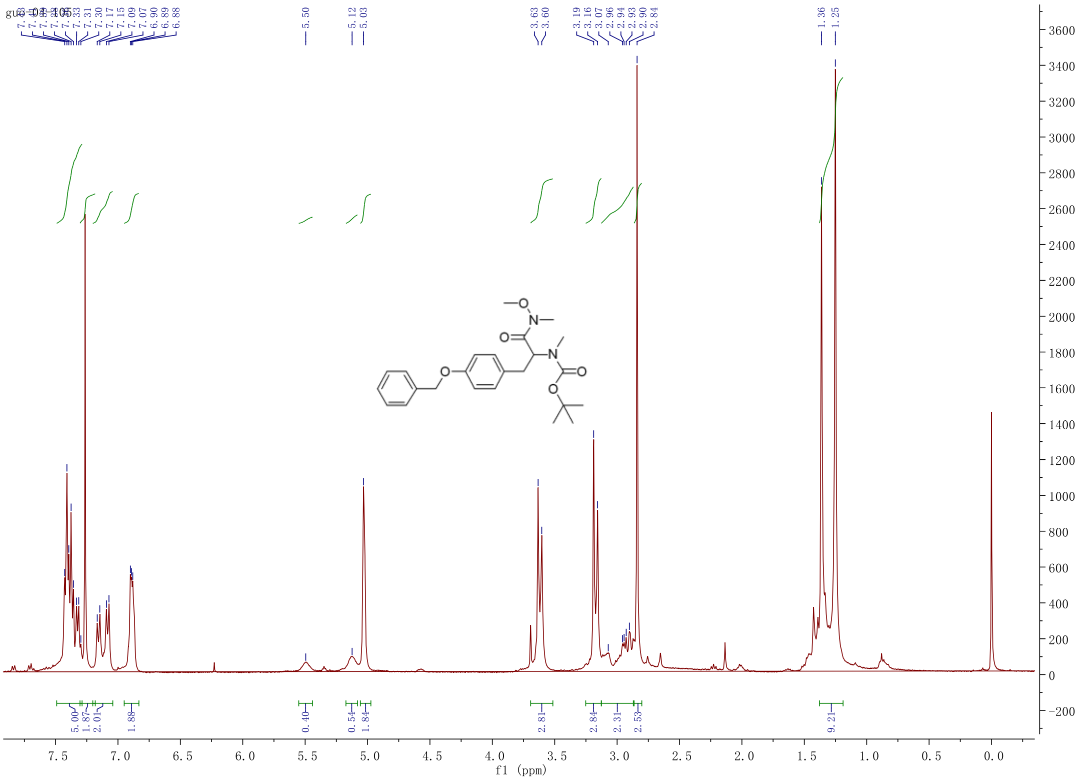

^{1}H NMR spectrum of **12d**

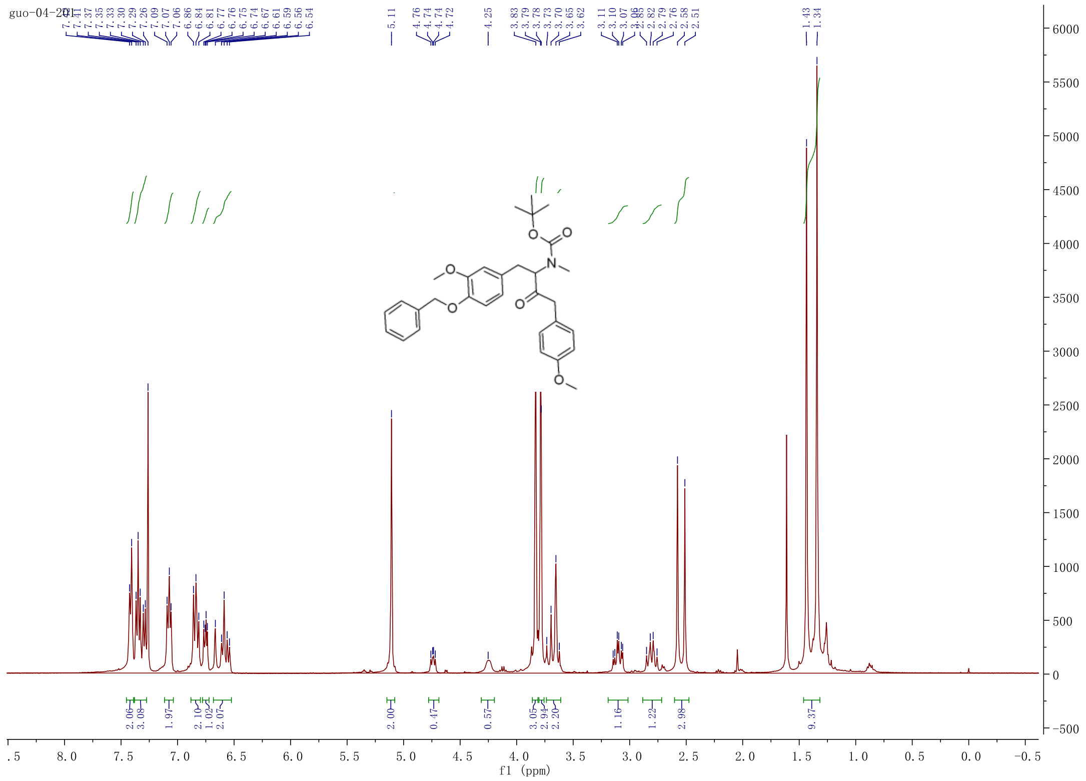

^{1}H NMR spectrum of **13a**

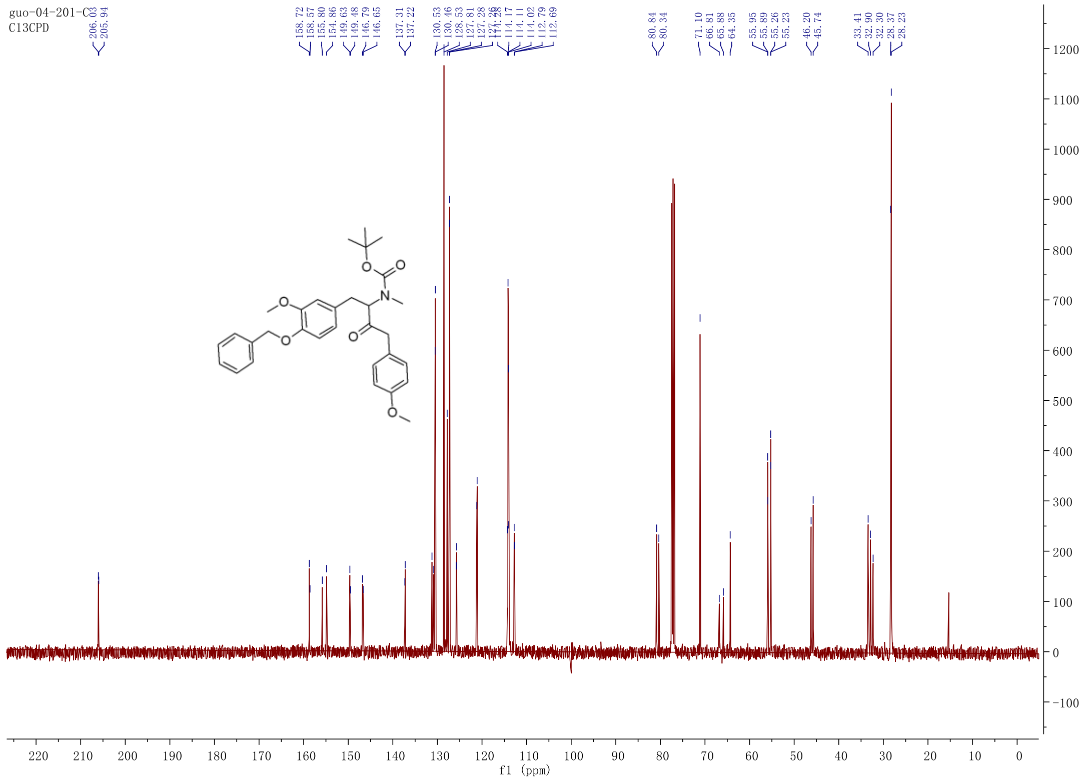

^{13}C NMR spectrum of **13a**

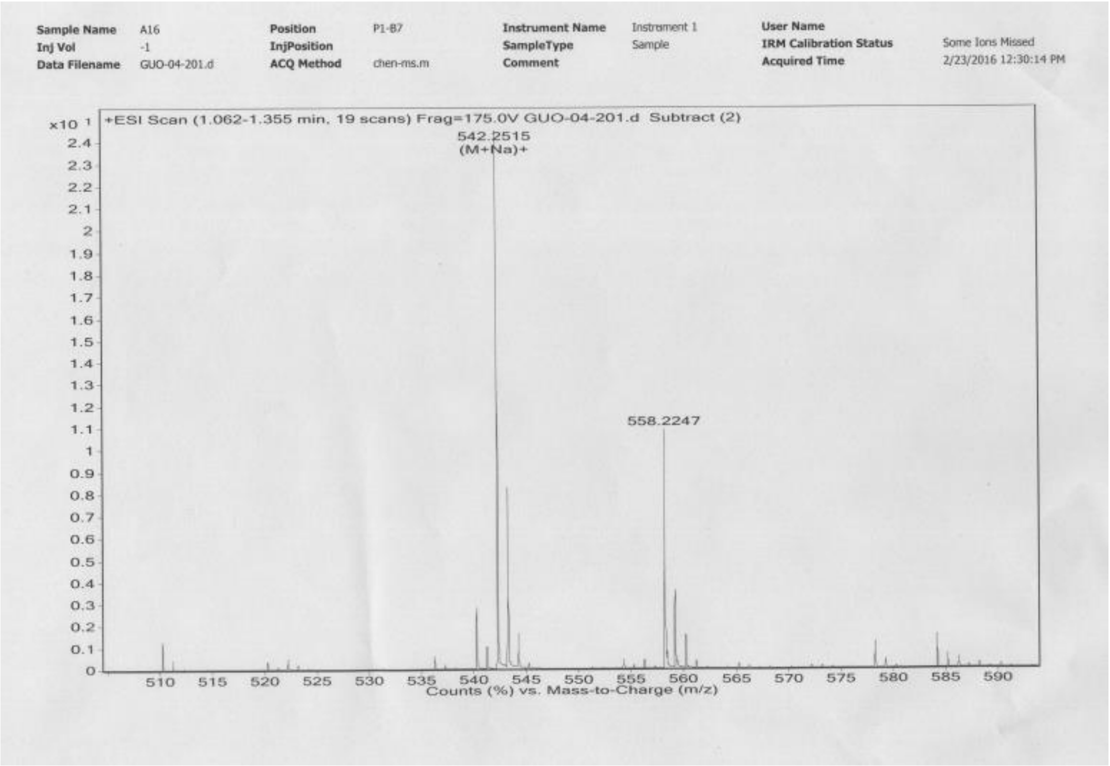

HRMS spectrum of **13a**

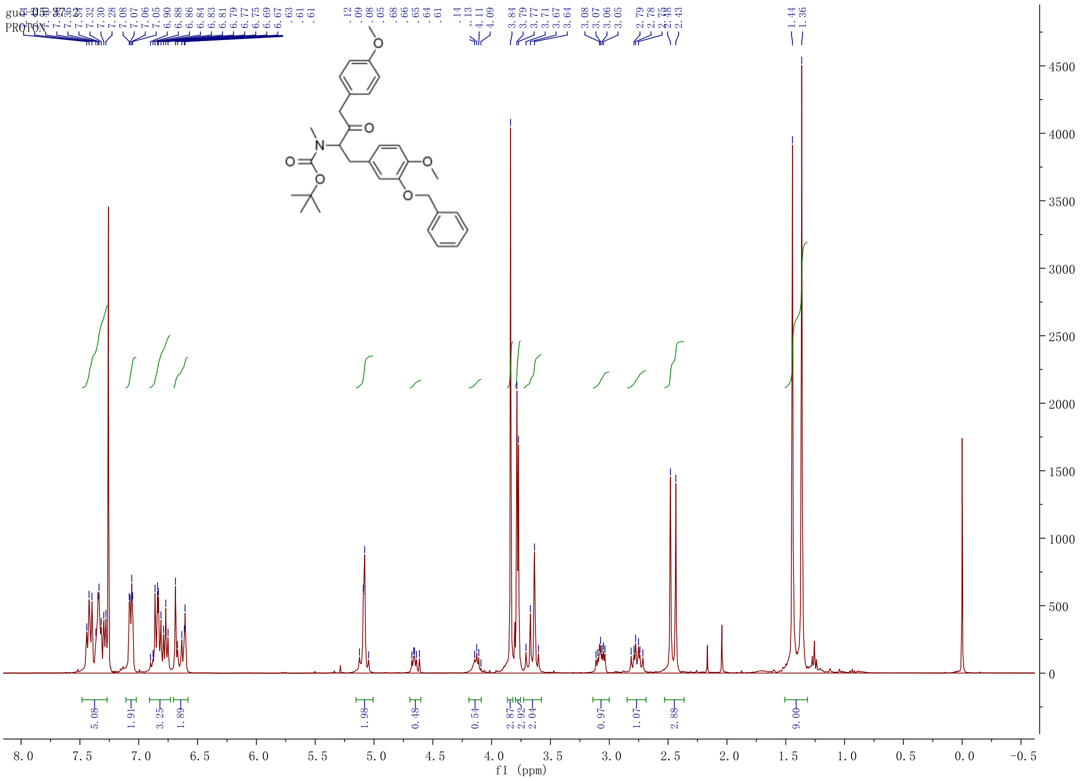

^{1}H NMR spectrum of **13b**

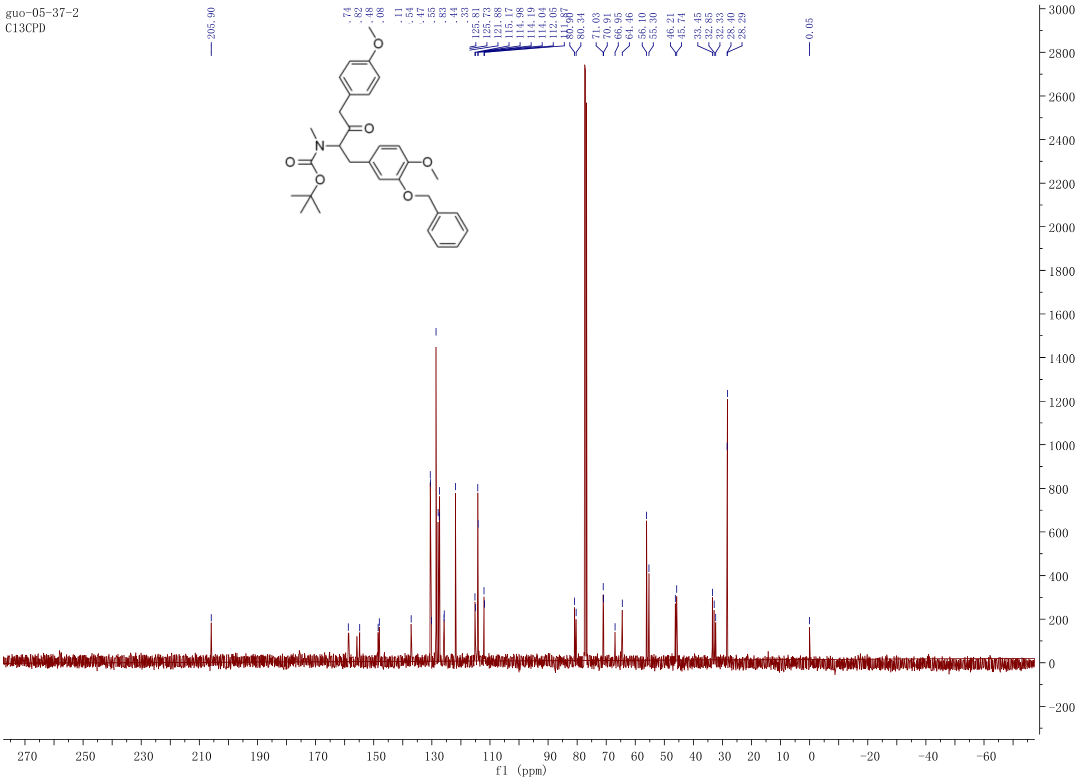

^{13}C NMR spectrum of **13b**

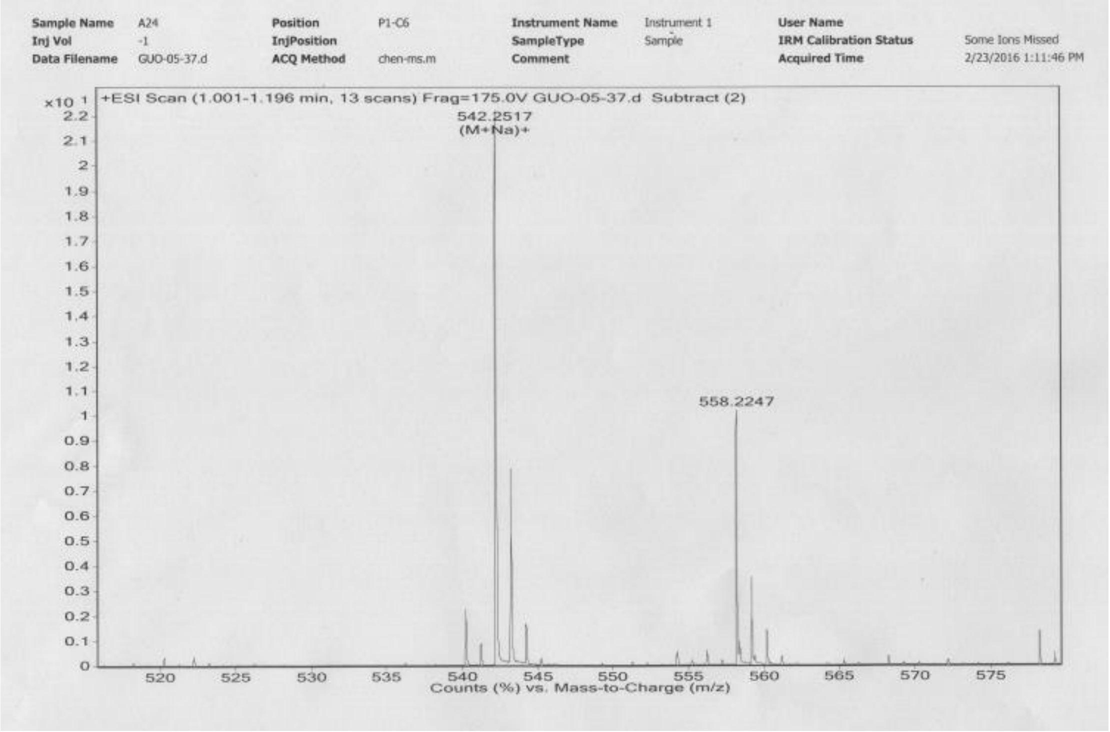

HRMS spectrum of **13b**

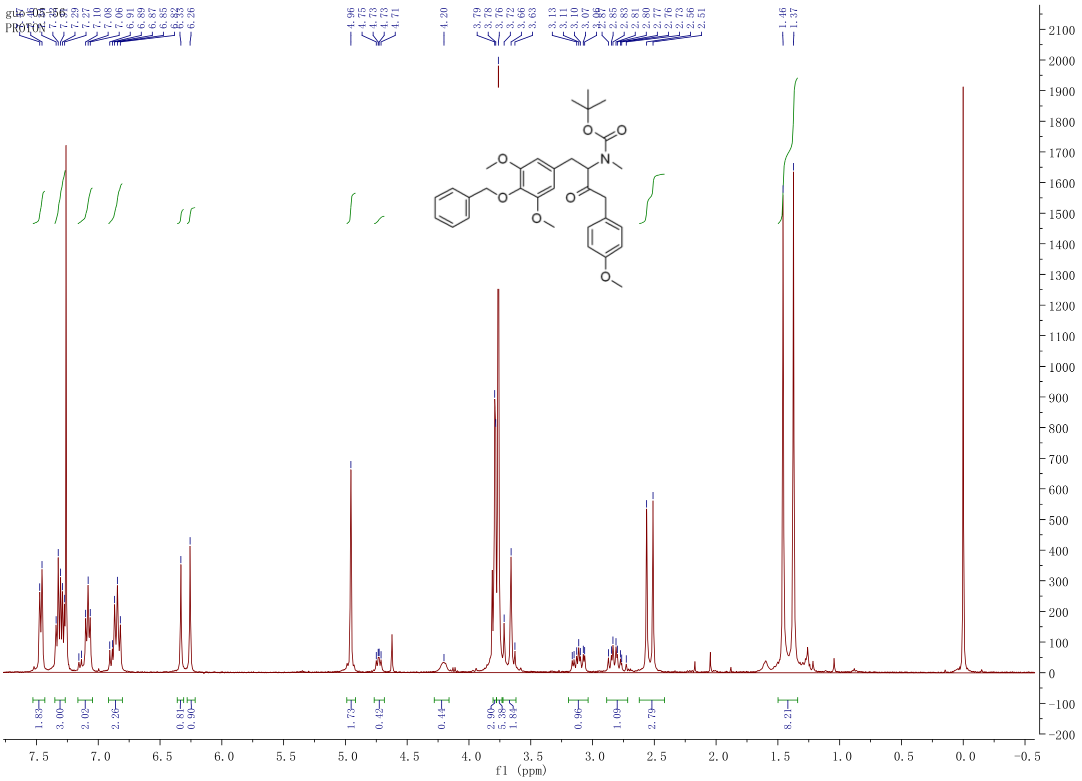

^{1}H NMR spectrum of **13c**

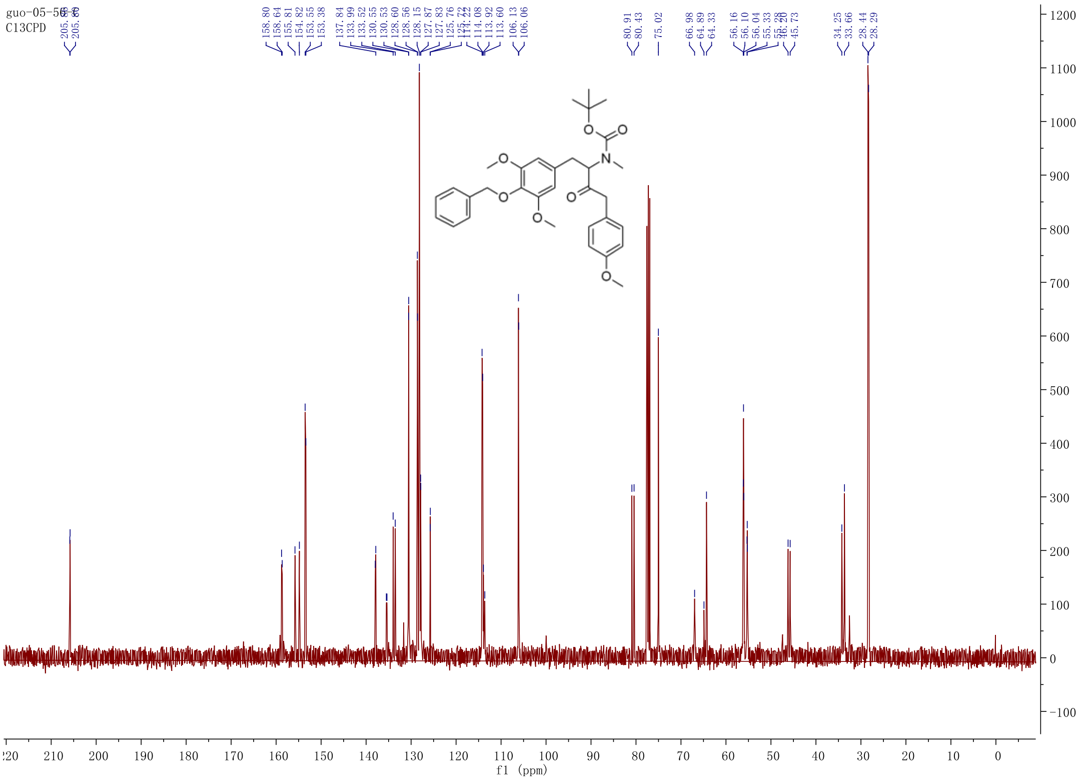

^{13}C NMR spectrum of **13c**

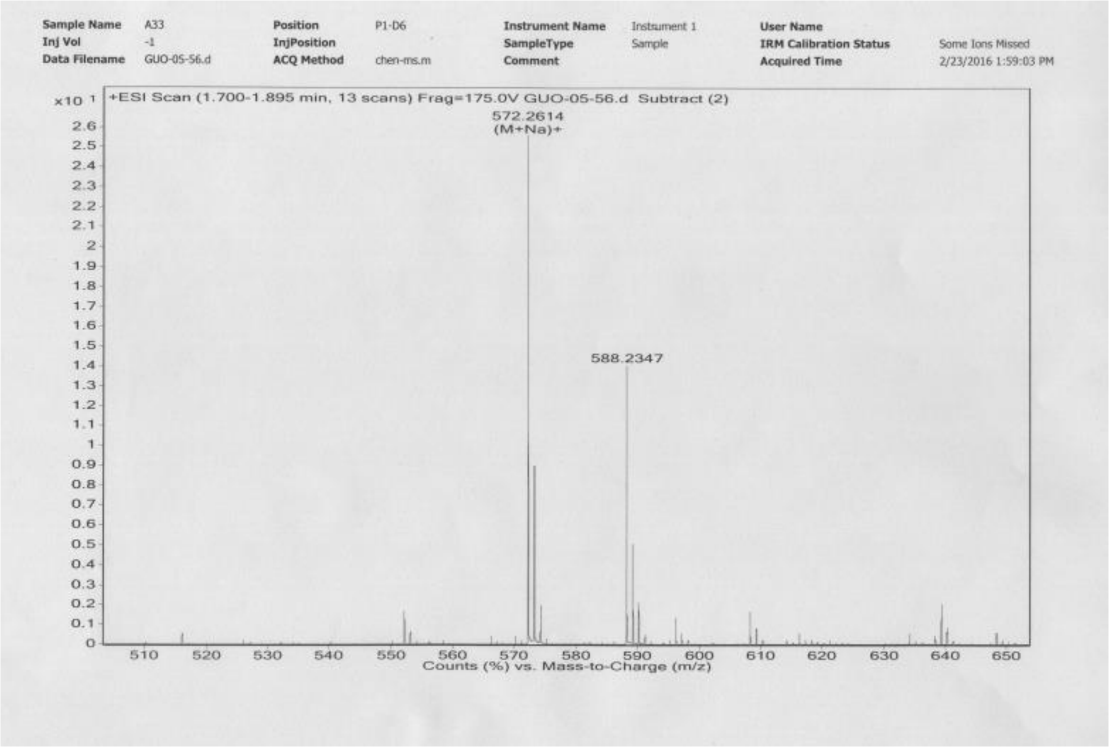

HRMS spectrum of **13c**

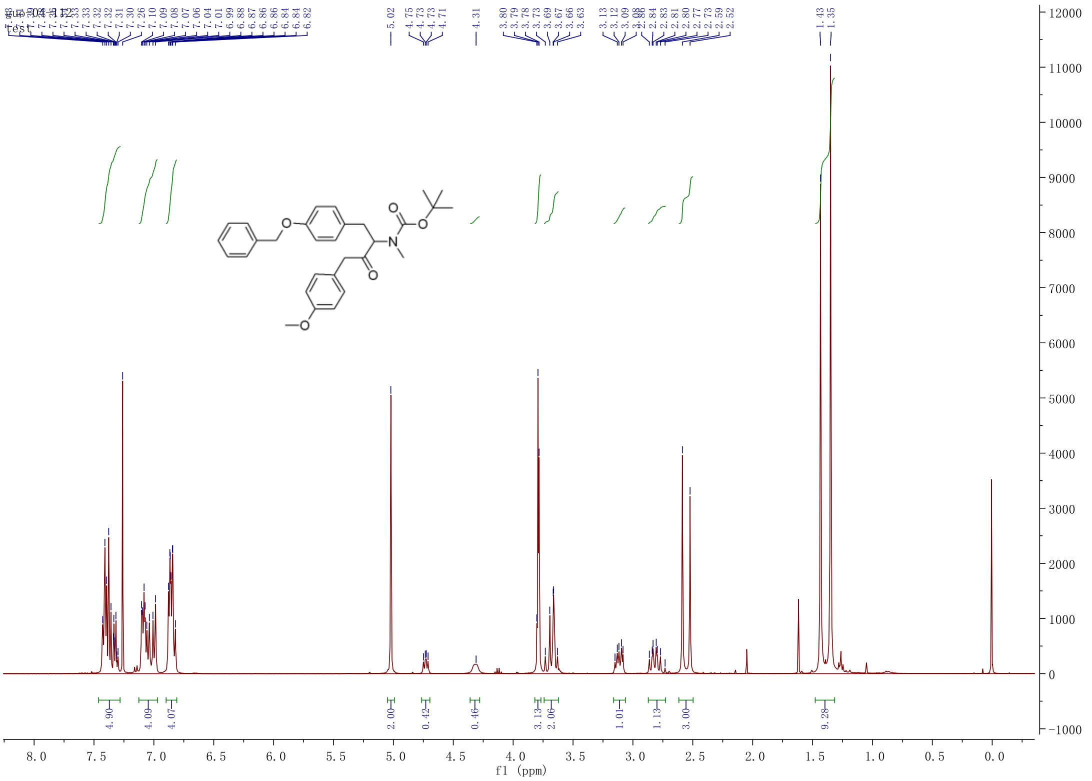

^{1}H NMR spectrum of **13d**

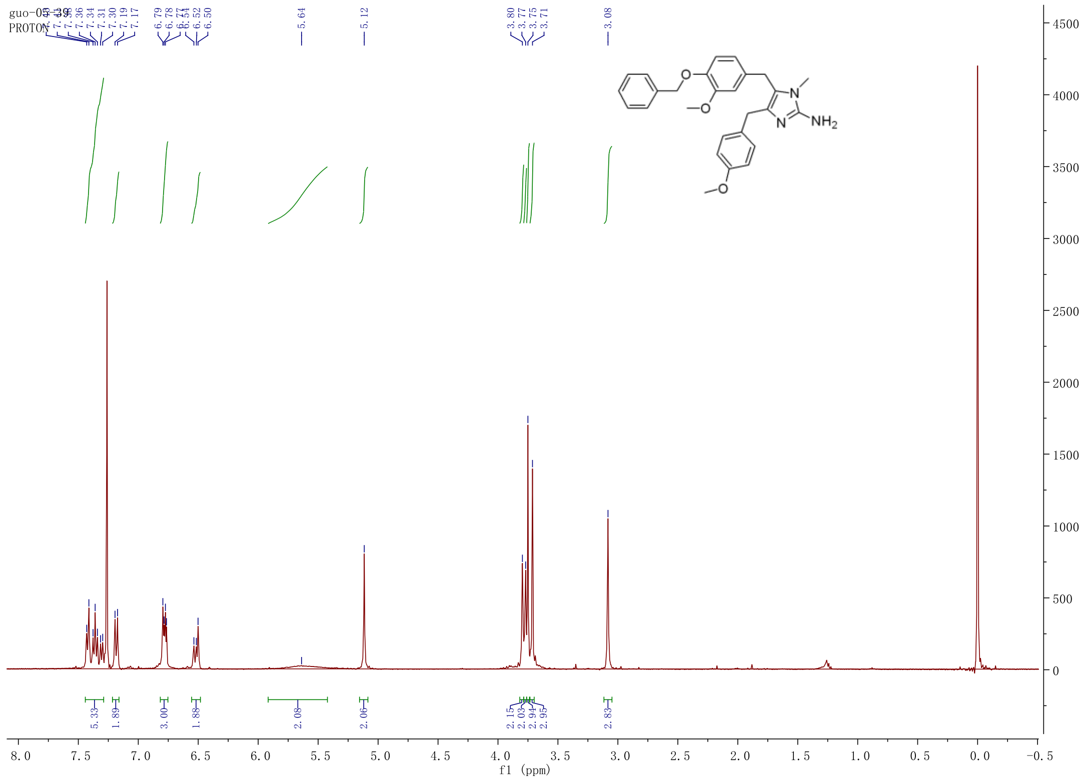

^{1}H NMR spectrum of **15a**

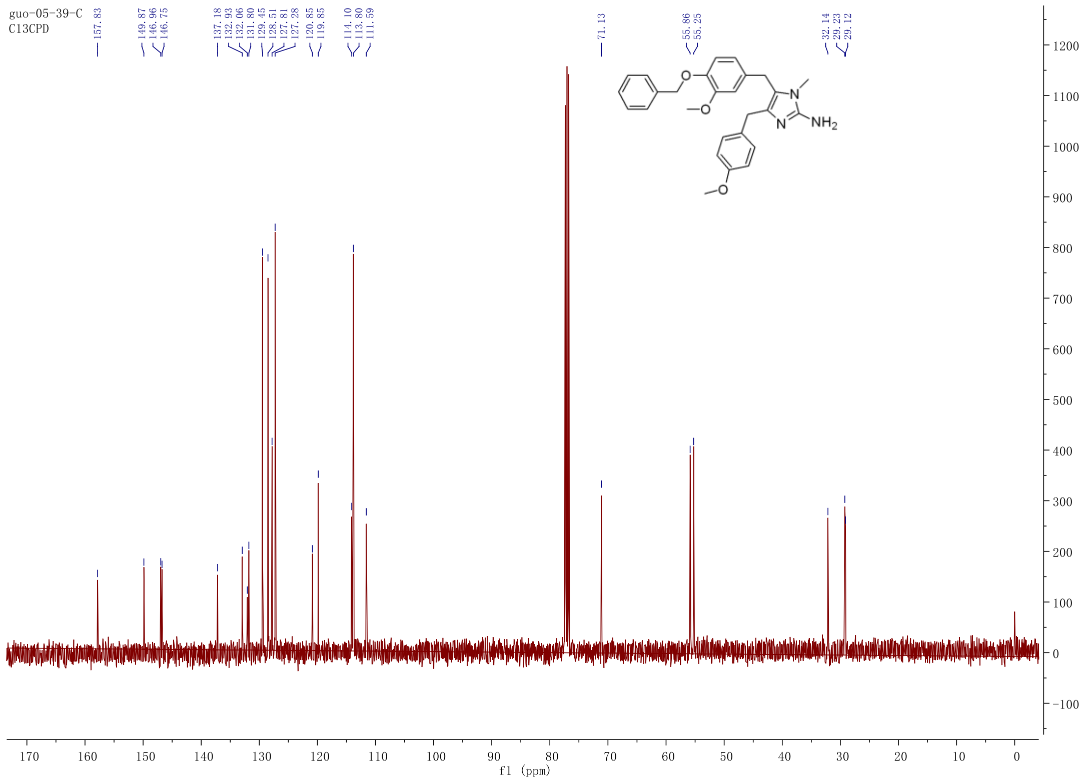

^{13}C NMR spectrum of **15a**

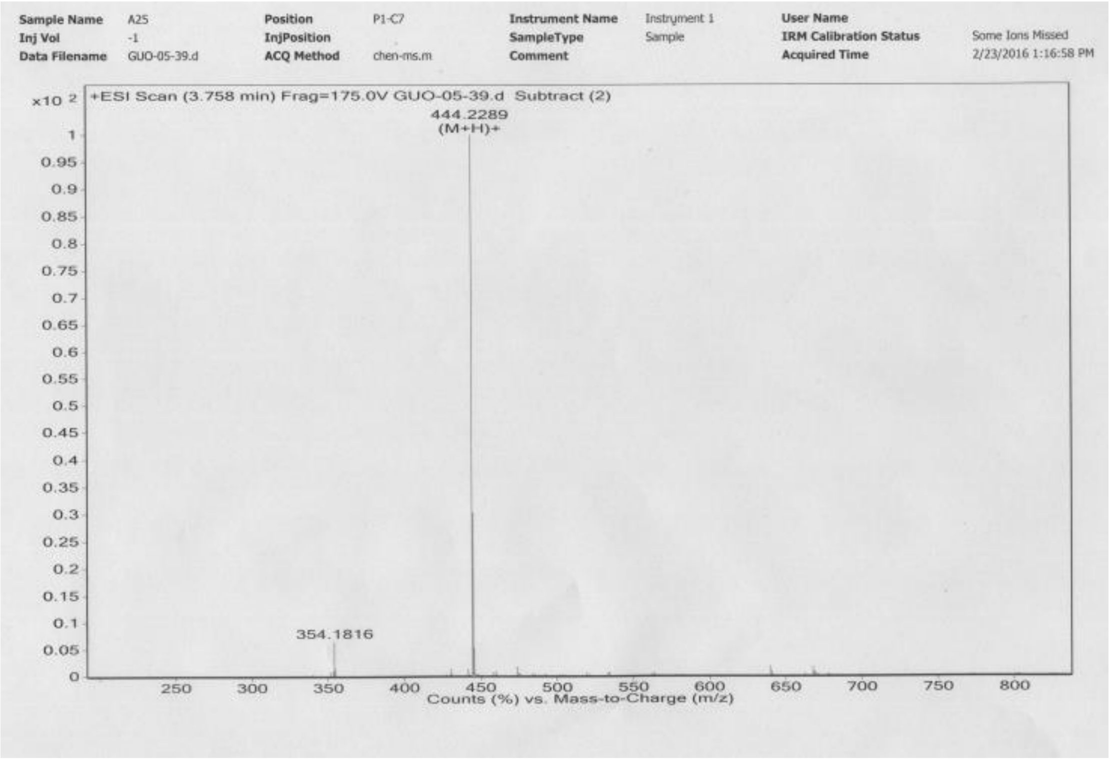

HRMS spectrum of **15a**

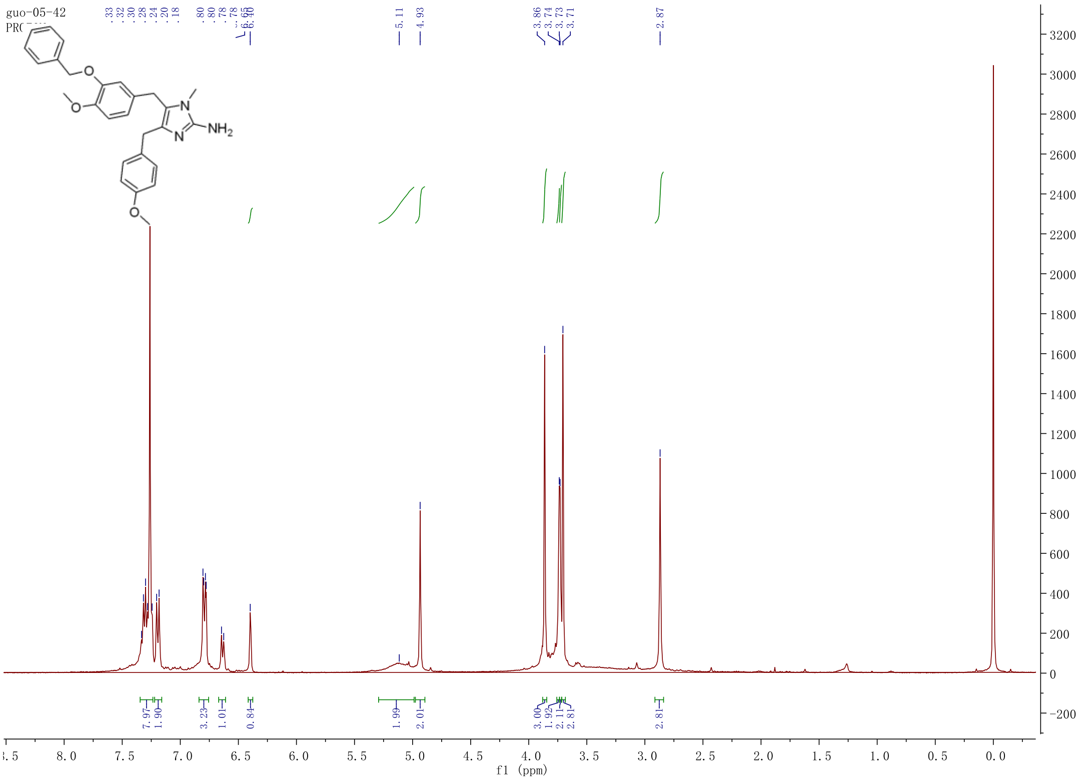

^{1}H NMR spectrum of **15b**

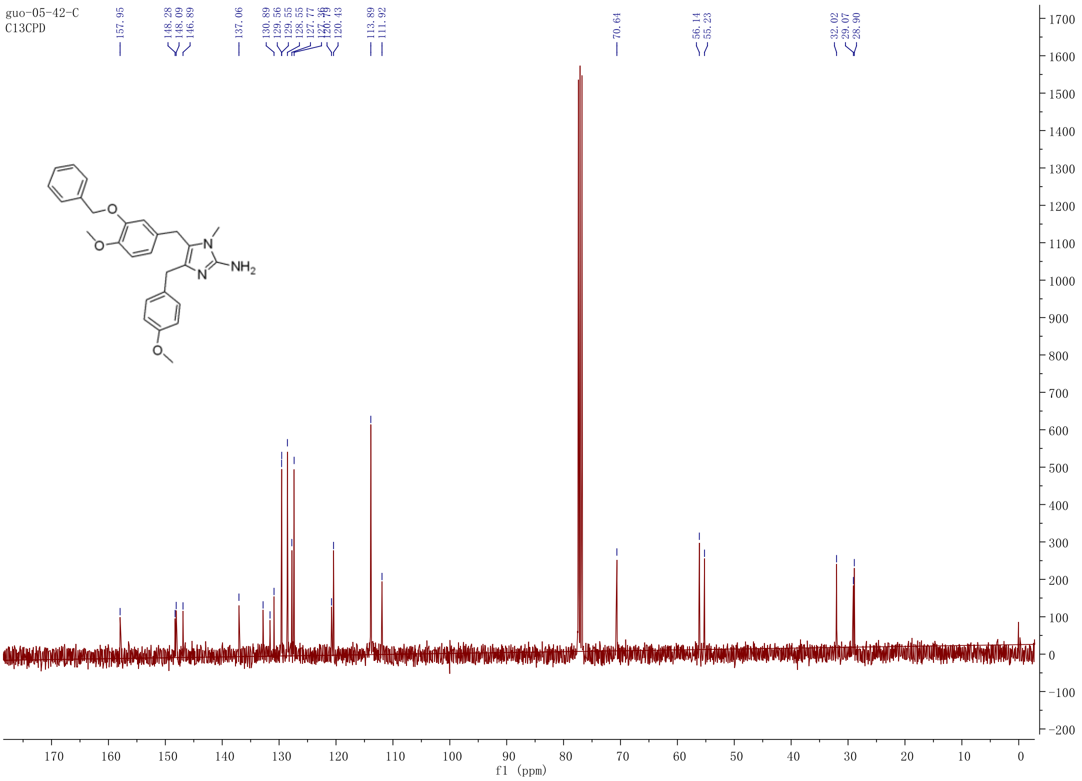

^{13}C NMR spectrum of **15b**

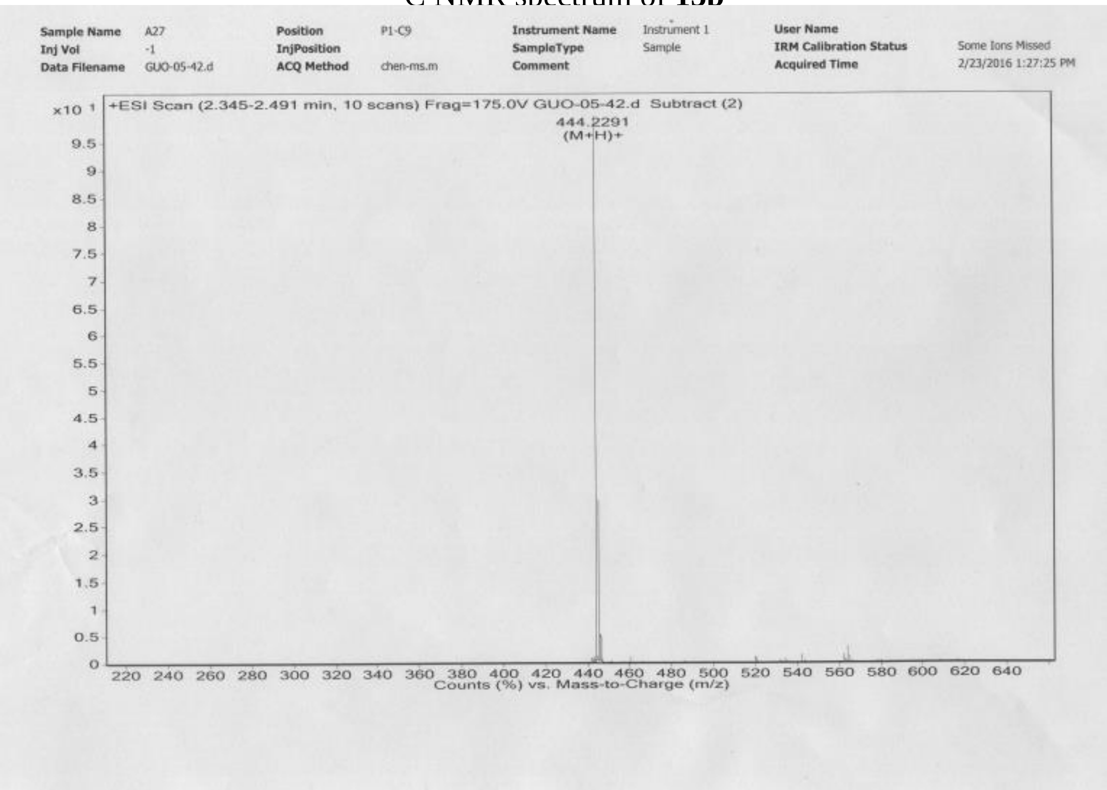

HRMS spectrum of **15b**

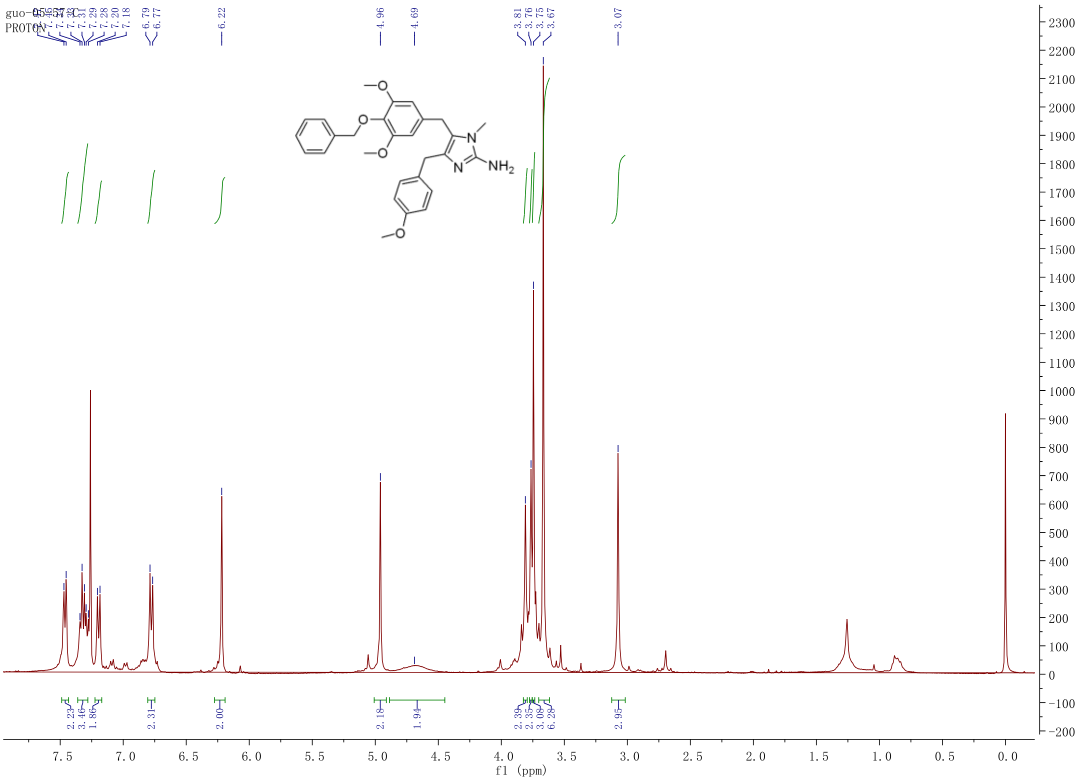

^{1}H NMR spectrum of **15c**

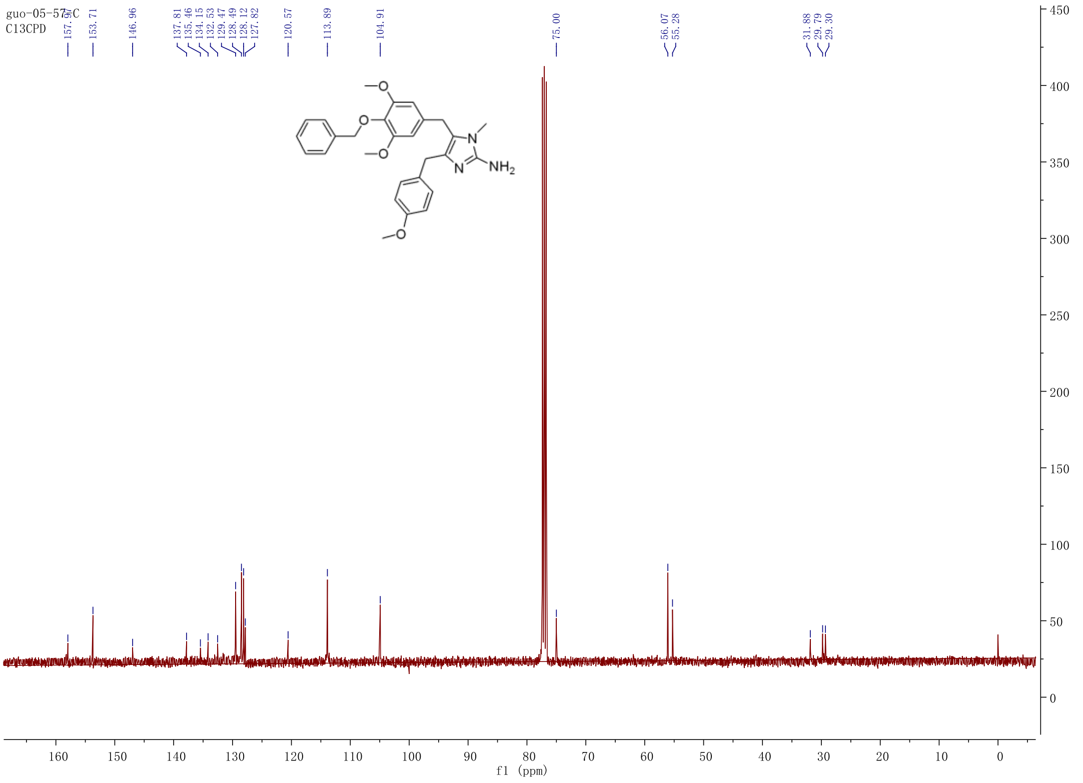

^{13}C NMR spectrum of **15c**

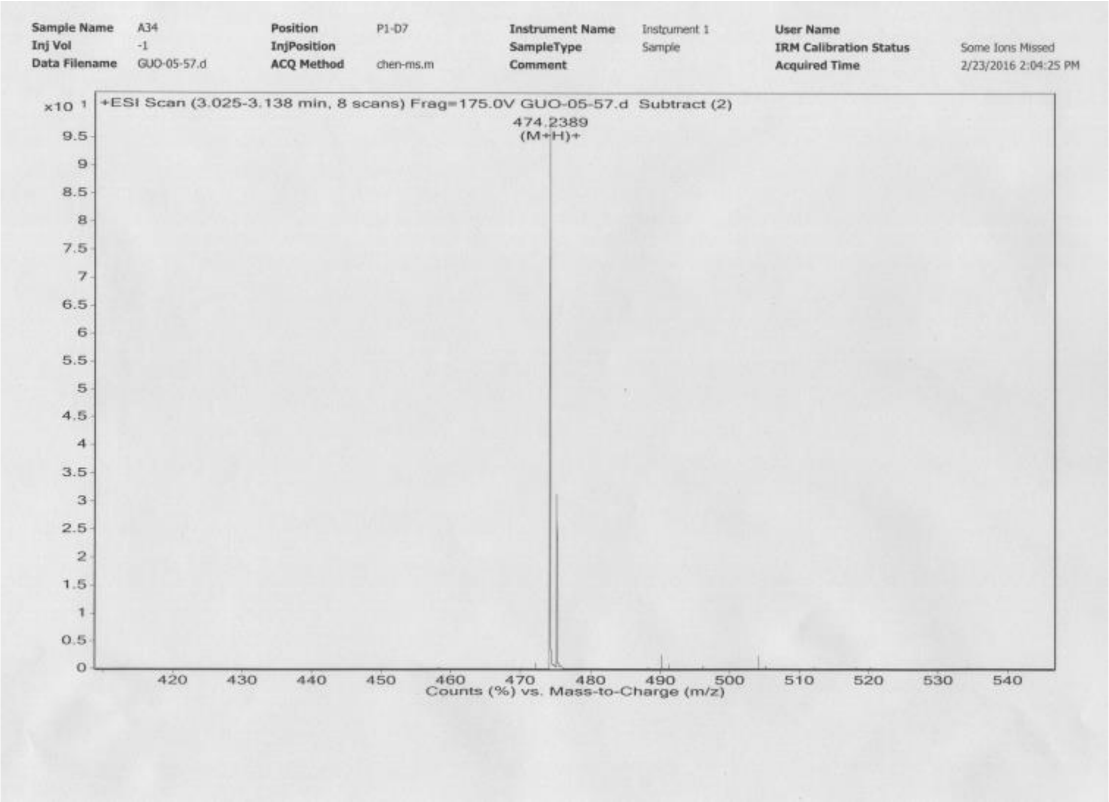

HRMS spectrum of **15c**

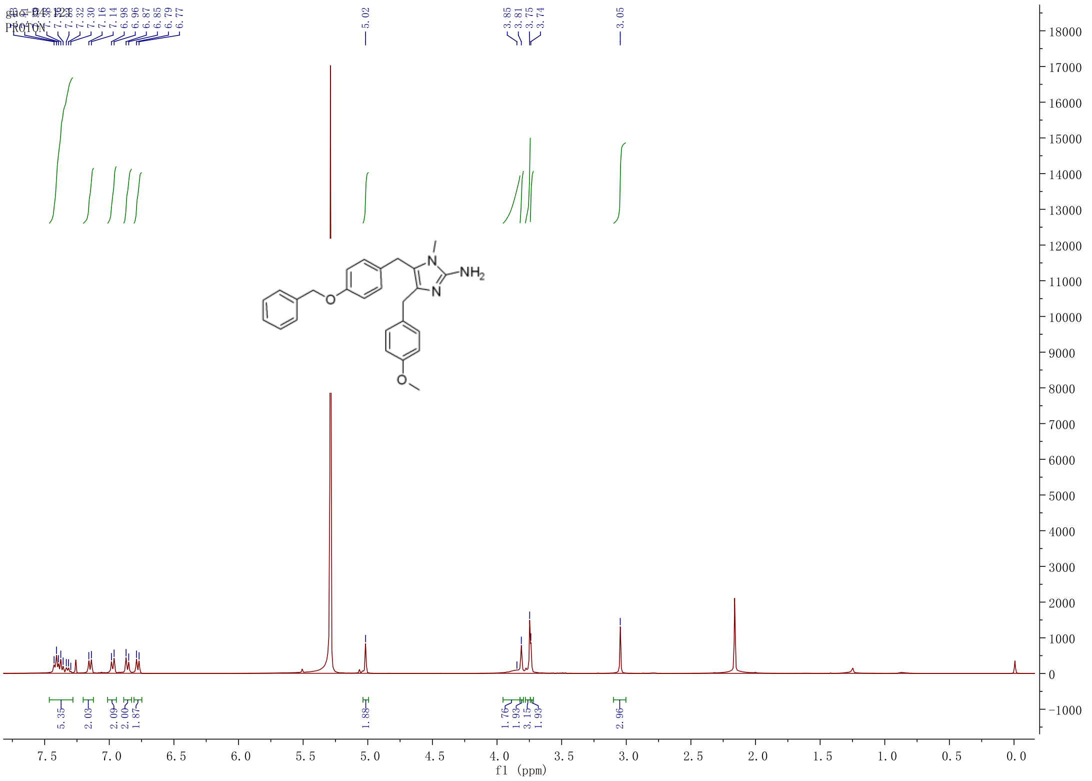

^{1}H NMR spectrum of **15d**

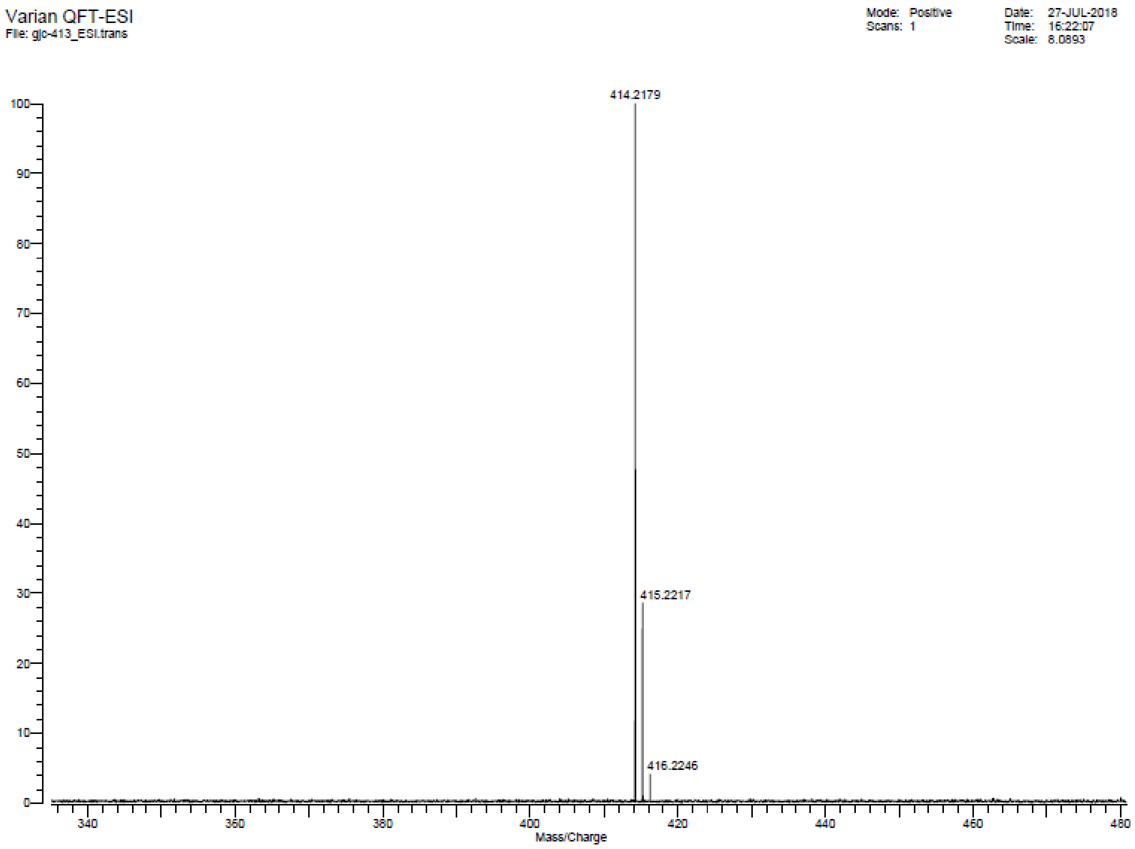

HRMS spectrum of **15d**

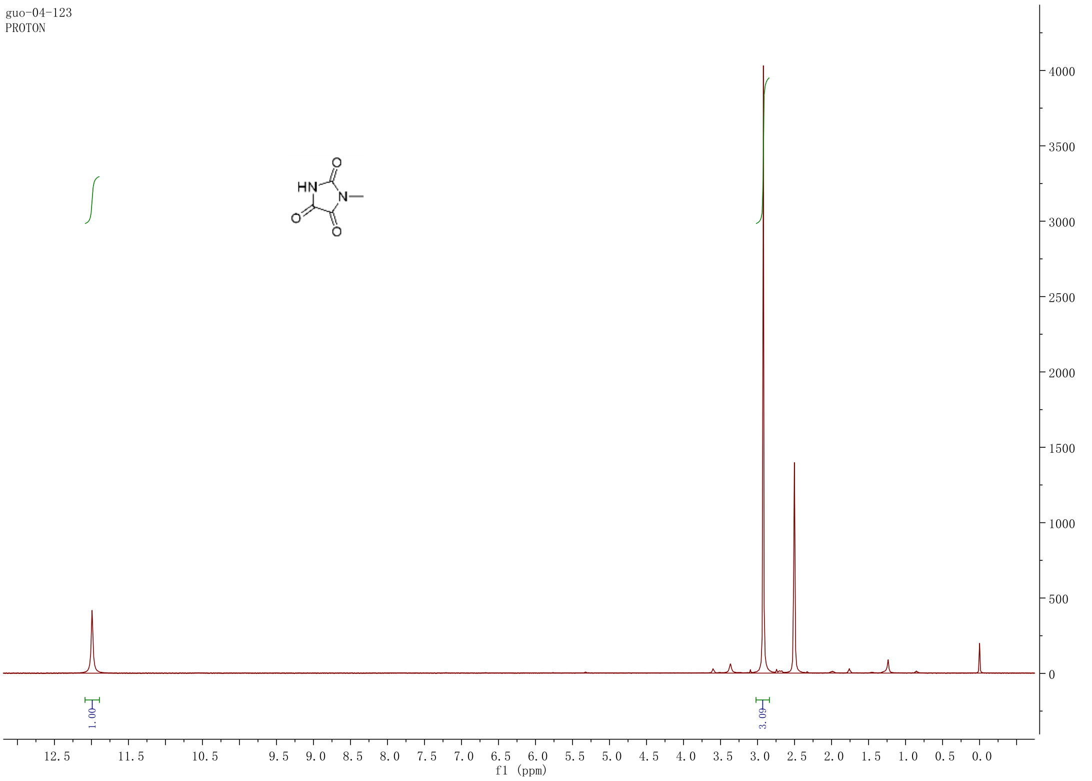

^{1}H NMR spectrum of **16**
